# Transmembrane domain switching controls PINK1 import and fate in mitochondria

**DOI:** 10.1038/s44318-026-00789-x

**Published:** 2026-05-26

**Authors:** James S Lorriman, Rhiannon J Hughes, Adam G Grieve, Robin A Corey, Ian Collinson

**Affiliations:** 1 School of Biochemistry, University of Bristol, Bristol, UK; 2 School of Physiology, Pharmacology and Neuroscience, University of Bristol, Bristol, UK

**Keywords:** Organelles, Translation & Protein Quality

## Abstract

Mitochondrial targeting of the PINK1 kinase results, under normal conditions, in membrane-potential-driven inner membrane penetration and cleavage by the resident protease PARL before retro-translocation and proteasomal degradation. In compromised mitochondria, with reduced membrane potential, inner membrane incorporation is not achieved, which leads to surface activation of the full-length protein, Parkin recruitment and mitophagy. Here, we identify a third pathway in which PINK1 is imported into the mitochondrial matrix. Structural modelling predicts that PINK1’s transmembrane domain (TMD) is conformationally plastic, forming either an α-helix or α/β-hybrid at the interface between Tim17 of the TIM23-complex for engagement of either ROMO1 or PARL. These mutually exclusive assemblies define distinct protein-import channels with differing biological roles. PINK1’s α-helical TMD adopts a pose suggestive of translocation through the ROMO1/Tim17-channel, while the α/β-hybrid engages PARL and is cleaved. We propose that TMD structural plasticity determines whether PINK1 is imported into the matrix or cleaved and retro-translocated. The results expand the role of PINK1 beyond that of a damage sensor and imply a role in healthy mitochondrial function with potential relevance to Parkinson’s disease.

## Synopsis

PINK1 is a sensor of mitochondrial health, being either imported and degraded by healthy mitochondria, or stabilised on the surface when membrane potential is lost. In this article, the authors identify a conformational switch within the PINK1 transmembrane domain directing full-length protein import into the matrix via a distinct Tim17-associated route, suggesting that PINK1 also regulates healthy mitochondria.

- PINK1 partitions into three outcomes: degradation, mitophagy signalling, or matrix import.
- The PINK1 transmembrane domain (TMD) is structurally plastic, switching between α-helical and α/β-hybrid conformations.
- These conformations engage Tim17 in mutually exclusive partnerships with either ROMO1 or PARL.
- TMD switching provides a mechanistic basis for pathway selection and links mitochondrial import to PINK1 function in homeostasis and disease.

## Abstract (web summary)

Matrix import of PINK1 suggests a constitutive role in mitochondrial homeostasis, extending its function beyond damage-induced mitophagy.

## Graphical abstract

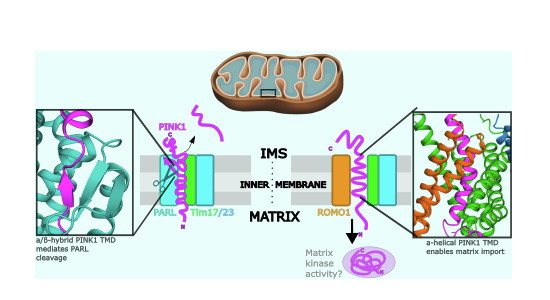

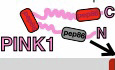

## Introduction

Nearly all mitochondrial proteins are synthesised in the cytosol, save only a few (13 in humans) encoded by the mitochondrial genome. Consequently, there is a high demand for protein import coordinated by a number of protein translocation machineries (Endo and Yamano, 2009; Neupert and Herrmann, 2007; Chacinska et al, 2009; Pfanner et al, 2019; Schatz, 1993). Proteins destined for import contain mitochondrial targeting sequences (MTS) directing them to the mitochondrial outer membrane, intermembrane space (IMS), inner membrane, or matrix (Calvo et al, 2016; Morgenstern et al, 2017). Those heading for the matrix are usually, but not always, directed there as precursor proteins with a cleavable N-terminal MTS (pre-sequence) (Neupert, 1997).

Import begins with targeting to the translocase of the outer membrane (TOM) for entry into the IMS (Pitt and Buchanan, 2021). Translocases of the inner membrane (TIM) are then engaged for transport across and into the inner membrane (Kübrich et al, 1994; Berthold et al, 1995). The major route into the matrix occurs via the TIM23-pathway driven by the membrane potential (Δψ) and ATP. The consensus view of precursor import *via* this route is of a continuous process occurring simultaneously through the TOM and TIM23 complexes in a single step, on the basis that an artificially arrested precursor is associated with both complexes (Rassow et al, 1989; Chacinska et al, 2010). However, recent findings reveal 3 distinct stages of import: 2 rate-limiting steps (1) transport through TOM, and (2) initiation of transport through the TIM23-complex, followed by fast translocation across the inner membrane into the matrix (Ford et al, 2022). This multi-step model has gained support from a structural analysis of the dynamic TOM-TIM23 super-complex (Yang et al, 2025), suggesting the latter rate-limiting step corresponds to formation of a protein channel between Tim17 and Mgr2 (see below).

The core TIM23-channel complex comprises the homologous subunits Tim23 and Tim17, the latter of which humans have two orthologs (TIMM17A and TIMM17B). The protein channel associates with the peripheral PAM-complex on the matrix side, wherein Tim44 connects with mtHsp70 for ATP-driven translocation (Kronidou et al, 1994; Schneider et al, 1994; Martin et al, 1991; Truscott et al, 2001). The structure of the core-channel complex (Sim et al, 2023) shows that the Tim17 subunit forms a hydrophilic slide, similar to that found in the YidC-family (Kumazaki et al, 2014b, 2014a). How the translocon adapts to sort precursors either into or across the inner membrane, as required, is a matter of current debate (Jain et al, 2025).

Presumably, transmembrane helices of membrane proteins can be sorted into the bilayer at the Tim17/lipid interface (slide), while for translocation into the matrix other protein factors are recruited for the formation of a hydrated pore. In the latter case, the requisite protein channel can be formed by Mgr2 in yeast (ROMO1 in humans) (Maruszczak et al, 2024; Sim et al, 2023; Fielden et al, 2023; Yang et al, 2025); although a problem with this proposal is that neither Mgr2 nor ROMO1 are essential (Mirzalieva et al, 2019; Kim et al, 2010). On-demand pore-formation, combined with fast transport across the inner membrane (Ford et al, 2022), could help mitigate against a catastrophic loss of the proton motive force (PMF)—the likely result of persistent leaky protein channels.

**Figure 1:**
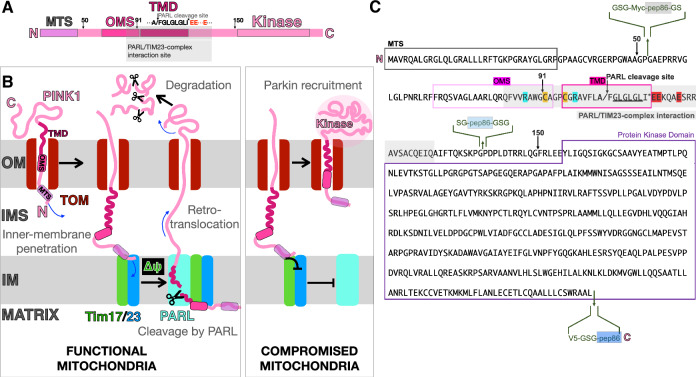
Domain structure and activity of human PINK1, and the construct amino acid sequence. (**A**) Organisation of PINK1 showing: the mitochondrial targeting sequence (MTS), outer mitochondrial membrane localisation signal (OMS), transmembrane domain (TMD) and the kinase domain. Key amino acid sequence shown for the PARL cleavage site (A/F), the GLGLGL-motif, I111 and conserved glutamates (see text). The N-terminal truncation sites at 50, 91 and 150 residues are shown (see Fig. 4F–H) and the region interacting with PARL and the TIM23-complex is shaded grey (see Fig. 4). (**B**) Schematic representation of the PINK1 import system; see text for further details. Key elements of PINK1 are labelled (top left, as in **A**); mitochondrial targeting sequence (MTS), outer membrane targeting sequence (OMS), transmembrane domain (TMD), with consistent colouring throughout; blue arrows indicate the direction of protein translocation. With functional mitochondria (left box), PINK1 is thought to undergo partial import into mitochondria followed by cleavage within the transmembrane domain by the inner mitochondrial membrane-resident, the rhomboid protease PARL. PARL cleavage precedes a process of retro-translocation, whereby PINK1 slides back out into the cytoplasm and is subject to degradation by the proteasome. In compromised mitochondria (right box), PINK1 is stabilised at the outer membrane, whereupon its dimerisation, mediated by the TOM-complex, facilitates autophosphorylation and the subsequent onset of mitophagy. (**C**) Sequence depiction and modification of the full-length human PINK1 protein deployed for *Mitoluc* import studies. The primary sequence of human PINK1 (UniProt: Q9BXM7) is shown annotated with the relevant key domains critical to PINK1 import and function (see text for details). PARL cleavage position and key post-translational modification sites are indicated. Insertion of pep86 and relevant epitope sequences is shown in green, flanked by GSG spacer sequences. Myc and V5 epitope sequences read EQKLISEEDL and IPNPLLGL, respectively, at the N-terminal and C-terminal insertions.

One mitochondrial protein requiring this machinery is the stress-responsive protein PTEN-induced kinase 1 (PINK1)—a serine-threonine kinase known for its role in targeting damaged mitochondria for degradation *via* its interaction with the ubiquitin ligase Parkin (Matsuda et al, 2010; Vives-Bauza et al, 2010; Narendra et al, 2010; Narendra and Youle, 2024). This process—mitophagy—ensures the maintenance of a functional cell-wide mitochondrial network, with errors in the process being linked to Parkinson’s disease. PINK1 comprises an N-terminal MTS, an outer membrane targeting sequence (OMS) followed by a transmembrane domain (TMD), and a C-terminal kinase domain (Fig. 1A). In spite of its naming as such this ‘TMD’ is not particularly hydrophobic (Fig. 1); deep-learning TMD prediction (Hallgren et al, 2022) gives a 0% probability of it being able to span the bilayer on its own.

In normally functioning mitochondria, PINK1 partially imports into mitochondria *via* the TIM23-complex (Akabane et al, 2023), with the N-terminal region (MTS, OMS and TMD) sitting deeply within the inner membrane. This penetration enables cleavage of the TMD (between residues A103 and F104) by the inner membrane resident rhomboid protease PARL (Fig. 1B, left box) (Deas et al, 2011; Narendra and Youle, 2024; Sekine and Youle, 2018). The bisected TMD and kinase domain of PINK1 then undergo retro-translocation out of the mitochondria for degradation by the proteasome, whereby the new N-terminus (F104) acts as a type-2 N-degron, recruiting E3 enzymes UBR1, UBR2 and UBR4 that target the protein for degradation (Tasaki et al, 2012, 2005; Yamano and Youle, 2013). The cleaved MTS, OMS and the other half of the TMD is presumably then released for degradation.

Conditions of mitochondrial stress, i.e., membrane depolarisation (induced artificially by the ionophore CCCP), result in import arrest and, as a result, failure of PINK1 inner membrane insertion and PARL cleavage (Fig. 1B, right box). In these circumstances, PARL is not recruited, and the uncleaved full-length PINK1 becomes trapped and dimerises within a TOM-TIM23 super-complex and, with the aid of the OMS, the kinase domain is stably presented at the mitochondrial surface (Kondapalli et al, 2012; Eldeeb et al, 2024; Okatsu et al, 2013, 2015). The structure of this large assembly highlights the arrangement of the PINK1 dimer at the outer membrane—upon a platform formed of two sets of dimeric TOM-complexes and, unexpectedly, 2 copies of VDAC2 (Callegari et al, 2025).

The dimerisation of PINK1 facilitates autophosphorylation and Parkin recruitment (Okatsu et al, 2013; Gan et al, 2022; Okatsu et al, 2015). Phosphorylation of Parkin by the phospho-activated PINK1 induces a downstream ubiquitination cascade, leading to proteasomal-mediated protein degradation and ultimately mitophagy (Yoshii et al, 2011; Zhuang et al, 2016). To counter excessive accumulation of PINK1 at the mitochondrial surface, in specific conditions, an alternative inner membrane protease OMA1 is induced (Sekine et al, 2019; Akabane et al, 2023)—potentially as a counterbalance to mitophagy.

These differing scenarios explain how PINK1 acts as a reporter of mitochondrial fitness for quality control. However, the current model does not consider the possibility and consequences of fully imported PINK1, where the kinase domain crosses the inner membrane to enter the matrix (Zhou et al, 2008). This prospect has been suggested by the identification of a potential PINK1-kinase substrate within the mitochondrial interior. PINK1 knock-out results in a loss of phosphorylation of NdufA10, a matrix-facing subunit of the respiratory Complex I, required for high rates of electron transfer (Morais et al, 2014; Pogson et al, 2014). In addition, it has been shown that PINK1 activity is regulated through degradation by the Lon protease in the matrix (Thomas et al, 2014). These observations suggest that, in addition to stress-related mitophagy signalling, PINK1 has additional regulatory roles relevant to mitochondrial activity. This could be achieved indirectly through transmembrane signalling cascades from the cytosolic kinase domain of PINK1 to the IMS and matrix. Alternatively, regulation of Complex I, *inter alia*, could be achieved directly by matrix-localised PINK1.

Given uncertainties about these alternative destinations of PINK1, we re-examined PINK1’s mitochondrial trafficking, including its potential for both IMS and matrix import. For this, we had to overcome considerable technical barriers. The first was the production of full-length human PINK1, and the second was the deployment of an accurate and real-time assay for mitochondrial import in human cells (*MitoLuc*) (Needs et al, 2023). Thereby, we were able to construct and purify a series of human PINK1 precursor proteins amenable to the analysis of its import. The import data are supported by structural modelling using AlphaFold2 (Evans et al, 2022; Jumper et al, 2021; Mirdita et al, 2022) and molecular dynamics (MD) simulations that address the mechanistic basis for PINK1 inner membrane insertion for proteolysis and transport.

The combined results reveal that, from the mitochondrial inner membrane, PINK1 can be directed either into the matrix or cleaved and transported back to the outer membrane and suggest a mechanism for this discrimination, based around the structural plasticity of PINK1’s TMD. These data, along with the effect of a Parkinson’s disease-causing residue substitution on the fate of PINK1, have motivated a re-evaluation of PINK1’s role in mitochondrial regulation. The results also raise the prospect of there being multiple bespoke protein channels through the inner membrane for different purposes, formed by the assembly of the TIM23-core-complex with various accessory partners, with Mgr2/ROMO1 being one of many alternatives.

## Results

### Import of PINK1 into the IMS occurs in two rate-limiting steps

**Figure 2:**
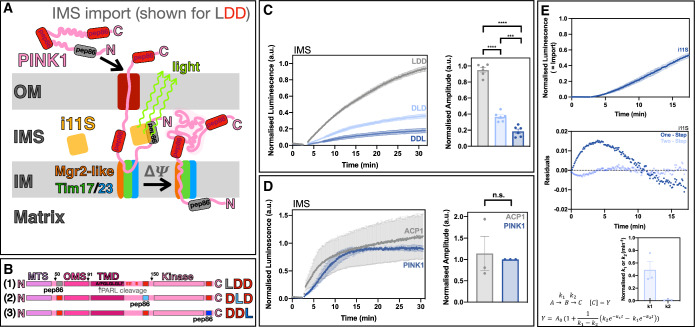
Import of PINK1 into the mitochondrial IMS proceeds in two distinct rate-limiting steps. (**A**) Schematic representation of the *MitoLuc* import assay–monitoring entry into the IMS by pep86 association with IMS-targeted 11S (i11S) for the formation of an active luciferase. PINK1 is depicted with 3 pep86 luciferase fragments. Separate experiments were conducted with the PINK1 LD series of 2 inactive (‘dark’, D) and 1 active (‘light’, L) pep86, in sequence: **L**DD, D**L**D and DD**L**; the former being shown in this schematic (see text for further details). (**B**) The PINK1 LD series (**L**DD, D**L**D and DD**L**) generated for assessing mitochondrial IMS and matrix (Fig. 3) import of PINK1–labelling as shown in Fig. 1A. (**C**) *MitoLuc* traces for import of the PINK1 LD series into the IMS, in each case background luminescence was recorded prior to addition of each of the PINK1 precursors. Normalised amplitudes were calculated from the point at which traces plateaued and plotted for each LD precursor. Error bars represent SEM of *N* = 6 biological repeats. A paired *t* test was used to determine the significance of the normalised amplitudes, *P* values = <0.0001, <0.0001 and <0.001. (**D**) *MitoLuc* trace and associated normalised amplitudes for import of PINK1 LDD and the matrix-targeted precursor ACP1 into the IMS. Error bars represent SEM of *N* = 3 biological repeats. A paired *t* test was used to determine the significance of the normalised amplitudes, *P* value = 0.7605. (**E**) The *MitoLuc* data for the PINK1 LDD variant (upper panel) was fitted to one-step (equation not shown) and two-step (bottom) models for import into the IMS. Residual plots (middle panel) represent the difference between the experimental data and fit. Normalised rate constants k_1_ and k_2_ values (lower panel) obtained from fitting the IMS import data to the two-step model. Data shown represent *N* = 3 biological repeats, error bars show SEM. Source data are available online for this figure.

To better understand the import behaviour of PINK1, we first interrogated its transport across the outer membrane into the IMS. This was achieved by the development of our in-cell import assay—*MitoLuc* (Needs et al, 2023). This involved the use of the Smac/DIABLO transit sequence (Burri et al, 2005) to target the large fragment of a split luciferase (11S) into the mitochondrial IMS (i11S) of intact human cultured cells, while the corresponding small fragment (pep86) is incorporated into non-conserved/non-critical regions of the PINK1 precursor (Figs. 1C and 2A,B).

The IMS distribution of 11S was verified by virtue of its extraction from mitochondria by digitonin, which selectively disrupts the outer membrane, but not the inner membrane (Appendix Fig. S1). Briefly, the analysis showed that the processed Smac/DIABLO-targeted 11S with the MTS removed could be readily extracted from mitochondria at low concentrations of digitonin (similar to the IMS-localised cytochrome c), whereas the inner membrane-localised unprocessed version was less prone to extraction. Therefore, the 11S is either detached in the IMS or attached to the IMS side of the inner membrane (Appendix Fig. S1). Later on, we see IMS detection of the PINK1 variant (PINK1-3EA), which is also consistent with Smac/DIABLO-targeted 11S being correctly localised (see below). Moreover, any residual 11S, mis-localised in the cytosol, will be mopped up by the addition of the inactive pep86 peptide bound to GST (GST-Dark; a standard component of the assay (Needs et al, 2023)).

These cells, with verified IMS-encapsulated 11S (in this case HEKs), are then perforated to allow the entry of the import protein substrate and assay reagents (including GST-Dark). Successful import then results in the association of the small and IMS-localised large fragments for the formation of an active luciferase for real-time and accurate monitoring of transport across the outer membrane (Fig. 2A, i11S); see Methods for details.

To provide a more granular readout of the import process, we engineered the luminescent reporter (pep86) in three different positions along PINK1: (1) at the N-terminal domain between the MTS and OMS, (2) between the TMD and the kinase domain and (3) at the C-terminus (Figs. 1C and 2A,B). The 3 variants all contained one active luminescent ‘light’ (L) and two inactive ‘dark’ (D) pep86 sequences; the latter disabled by pairwise amino acid swapping (x3). Thus, the three constructs, hereafter termed LDD, DLD and DDL, differ by the position of the active pep86 reporter (L), but are otherwise identical. The different locations of the active reporter (within LDD, DLD and DDL) could then be deployed in parallel experiments to monitor the entry of the corresponding sections of PINK1 into the IMS (and matrix—below).

The full-length proteins were successfully purified (Appendix Fig. S2 presents a typical example—variant DDL) and shown to associate with the large fragment of the luciferase (11S) in solution for the formation of an active luciferase and the liberation of bioluminescence (Appendix Fig. S3). Based on our previous analysis and methodological development (Pereira et al, 2019; Ford et al, 2022), we know that these association kinetics are suitable for implementation in the *MitoLuc* assay. This is because the interior mitochondrial volumes are tiny, so the concentration of pep86 and the arriving 11S will be very high (much higher than those shown in Appendix Fig. S3). Therefore, the association rates will be very high (substantially higher than the rate of import) and non-rate-limiting. Therefore, in the *MitoLuc* assay, the luminescence response reports on the rate of mitochondrial import, rather than on the assembly of 11S and pep86. Slight variations in the bioluminescent yield of the various PINK1-pep86-11S complexes (Appendix Fig. S3, B_max_) were used to scale the subsequent import measurements accordingly.

Progression of the three reporting regions of PINK1 into the IMS within HEK293T cells was then monitored over time, one by one, for each of the LDD (shown schematically in Fig. 2A), DLD and DDL variants (Fig. 2B). The luminescence traces chart the progression of the respective N-terminal (Fig. 2A), central and C-terminal regions of PINK1 across the outer membrane (Fig. 2C). Crucially, import of the C-terminus (DDL) corresponds with entry of the full-length protein into the IMS (or matrix—below). The amplitude (maximum luminescence) reflects the total amount of the pep86 reporter entering the IMS, and the rate reflects the time taken to cross the outer membrane. Both of these measures of PINK1 transport were similar to the canonical matrix-bound precursor protein (ACP1) characterised previously (Needs et al, 2023) (Fig. 2D).

Import of ACP1 gives us a measure of precursor build-up in the IMS on its way to the matrix. This is as expected because we have previously shown that initiation of transport across the inner membrane is rate-limiting (Ford et al, 2022). In the case of PINK1, from these data alone, we cannot say if (or how much) of the IMS signal is due to its final destination in the IMS, or as a result of the accumulation of proteins prior to matrix entry.

The PINK1 IMS import data could be fitted to a 2-step reaction mechanism (Fig. 2E) for transport across the outer membrane. The two rates are unaffected by the position of the luciferase fragment within PINK1 (Appendix Fig. S4), demonstrating that passage of the N-terminal, central and C-terminal regions of PINK1 into the IMS occurs by the same mechanism. Presumably, the two steps correspond to: (1) association with the TOM complex and (2) passage through the channel into the IMS.

The amount of material in the IMS decreases progressively from the N-terminal to the central and C-terminal regions (Fig. 2C), which probably reflects the directionality of transport (N → C-terminus) through TOM40, as well as the relatively slow rate (rate-limiting) of this step (Ford et al, 2022). Transport quantities into the IMS were insensitive to respiratory and ATP synthase (inner membrane) inhibitors (Antimycin A (1 μM) and Oligomycin (5 μM); ‘AO’) (Appendix Fig. S5); this was as expected because the outer membrane is not subject to their effects. This insensitivity also supports our assertion that the luminescent signal is a bona fide measure of IMS import; significant rogue Smac/DIABLO-targeted 11S in the matrix would have liberated a luminescent signal sensitive to AO treatment (see below).

### PINK1 is fully imported into the matrix

**Figure 3:**
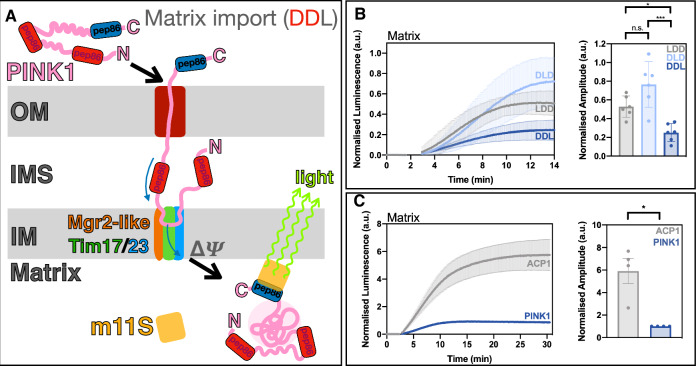
PINK1 is fully imported into the mitochondrial matrix. (**A**) Schematic shown as in Fig. 2 for monitoring import into the matrix, rather than the IMS. In this depiction, the import of the PINK1 variant DD**L** (Fig. 2) is shown. (**B**) *MitoLuc* trace for the import of the PINK1 LD series (Fig. 2B; **L**DD, D**L**D and DD**L**) into the matrix, in each case background luminescence was recorded prior to addition of the specific PINK1 precursor variant. Normalised amplitudes were calculated from the point at which traces plateaued and plotted for each of the LD precursors. Data shown represent *N* = 6 biological repeats, error bars show SEM. A one-way ANOVA with Tukey’s post hoc test was used to determine the significance of the normalised amplitudes, *P* values = 0.0619, 0.0278, 0.0002. (**C**) *MitoLuc* traces and associated normalised amplitudes for the import of PINK1 DDL and the precursor ACP1 into the matrix. Data shown represent *N* = 4 biological repeats, error bars show SEM. A paired *t* test was used to determine the significance of the normalised amplitudes, *P* value = 0.0220. Source data are available online for this figure.

To explore the prospect of partial or complete transport of PINK1 across the mitochondrial inner membrane, we utilised the same LDD, DLD, and DDL variants to monitor precursor entry into the matrix (Fig. 3a—exemplifying DDL). The *MitoLuc* assay for matrix import has been validated previously and shown to be a faithful measure of precursor transport across the inner membrane (Needs et al, 2023); see also below. The data reveal that PINK1 does indeed enter the mitochondrial matrix (Fig. 3B). Furthermore, the luminescence signal from the C-terminal pep86 reporter demonstrates that the entire protein crosses the inner membrane and enters the matrix (Fig. 3B, DDL). Note that some of the signals generated for the import of the LDD variant (Figs. 2A,B and 3B) might have arisen from transport of the N-terminal region of PINK1 after cleavage and release from PARL (Fig. 1B). Therefore, the LDD transport data may reflect both cleavage and import activities of PINK1.

Unlike the IMS data, which fitted to a 2-step model, the matrix import datasets are too complex to fit to a kinetic model. Nevertheless, the data reveal several qualitative features about PINK1’s mitochondrial trafficking. While substantial, the total quantity of PINK1 import is somewhat lower than the canonical matrix-targeting benchmark ACP1 (also carrying the luminescence reporter pep86 at the C-terminus; Fig. 3C), suggesting that not all of the PINK1 enters the matrix, with some being retained in the IMS. Further, in contrast to IMS import, subsequent passage into the matrix is sensitive to Antimycin A and Oligomycin (AO; Appendix Fig. S6), suggesting that, as expected, translocation occurs *via* the TIM23 pathway, which requires both ATP and the membrane potential (Δψ) (Martin et al, 1991; Geissler et al, 2000; Truscott et al, 2001; Ford et al, 2022; Wachter et al, 1994). The effects of AO (respectively, for respiratory chain and ATP synthase inhibition) were far from complete because, in this particular system, they are not very good at reducing the membrane potential (Needs et al, 2023). The more effective means of doing so, with the ionophore CCCP, could not be implemented because of its incompatibility with the luciferase assay.

Critically, deletion of the N-terminal targeting region of PINK1, up to the kinase domain, completely ablates the luminescent signal (see below). So we can be sure that the assay is a genuine measure of matrix import, rather than a result of PINK1 association with residual 11S elsewhere.

### Modelling of PINK1 and its interaction partners reveals structural plasticity in the PINK1 TMD

The results above demonstrate that PINK1 can be fully transported across the inner membrane. Therefore, following entry into the IMS and contact with the inner membrane PINK1 has alternative fates in functional mitochondria: (1) delivery to the rhomboid protease PARL for cleavage and retro-translocation to the outer membrane, as previously described (Deas et al, 2011; Narendra and Youle, 2024; Sekine and Youle, 2018), or (2) transport into the matrix. We presumed both to be mediated through interactions with the TIM23-complex. To better understand these distinct pathways, and how they are discriminated, we first explored the predicted structures of PINK1 in complex with PARL.

Structural models of PINK1 were produced by the deep-learning AlphaFold2 program, see 'Methods' and Table 1 for details. All models and plots of quality metrics are available to download from https://osf.io/xj9ca/, with key plots also included in the Appendix.

**Table 1:** AlphaFold2 models built for this study.

AlphaFold2 model	Details
PINK1/PARL	Residues 62-581 of UniProt ID Q9BXM7 and residues 93-368 of Q9H300
PARL/Tim17/Tim23/Tim44	Residues 93-368 of Q9H300, residues 1-143 of Q99595 (TIMM17A), residues 66-209 of O14925, and residues 37-452 of O43615 (37-260 trimmed post hoc due to low pLDDT – with the amphipathic helix of Tim44 (residues 130-191) included in the figures for illustrative purposes only).
Tim17/Tim23/ROMO1	Residues 1-143 of Q99595, residues 66-209 of O14925, and all residues of P60602.
Yeast Tim17/Tim23/Mgr2	All residues of UniProt ID P39515, P32897, and Q02889
PINK1_TMD/ Tim17/Tim23/ROMO1	Residues 1-130 of UniProt ID Q9BXM7 joined by a GSGSGSGS linker to residues 1-143 of Q99595, residues 66-209 of O14925, residues 298-452 of O43615, and all residues of P60602

All models are of the human proteins unless otherwise specified.

**Figure 4:**
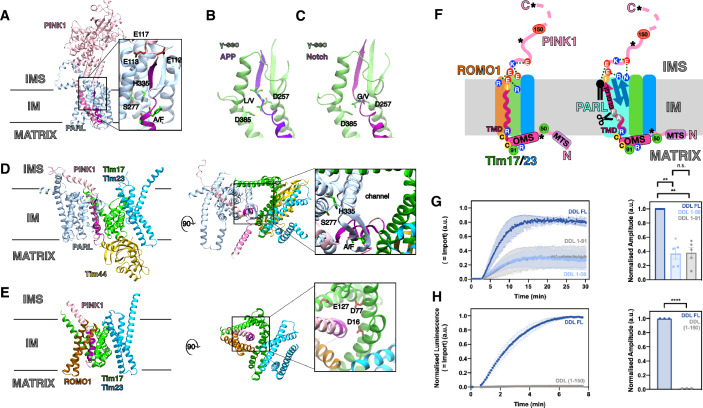
AlphaFold2 modelling of PINK1 and its interactions with PARL and the core TIM23-complex. (**A**) Top-ranked (of five) AlphaFold models of the PINK1-PARL complex. Inset: Close up of the PINK1-PARL β-sheet, showing the proximity to the PINK1 cleavage site between A103 and F104 (A/F) and conserved 3 glutamates (E112, E113 and E117) residues, as well as the active site catalytic residues of PARL (S277 and H335). The PINK1 transmembrane domain (TMD) is shown in purple, and the outer membrane targeting sequence (OMS) in darker pink. The lines indicate the position of the bilayer. (**B**, **C**) Respective views of γ-secretase bound to amyloid precursor protein (APP; PDB 6IYC) and Notch (PDB 6IDF). The active site aspartate residues are shown (D257 and D385), as well as the cleavage site, between leucine and valine (L/V) for APP and glycine and valine (G/V) for Notch. (**D**) View of the hybrid AlphaFold model of the PINK1-PARL-Tim17-Tim23-Tim44 complex, see methods for modelling details. PINK1 has been truncated after residue 150 for clarity, TMD shown in purple and OMS in darker pink. The complex is shown side on from the membrane (left) and from the IMS (right). Inset: zoom of the putative protein-conducting pore, formed between Tim17 and PARL, showing also the adjacent PARL active site (S277 and H335) and the cleavage site of PINK1 (A/F). The lines indicate the position of the bilayer. (**E**) Same views as in panel (D) of the AlphaFold model of Tim17-Tim23-ROMO1 and the PINK1 TMD. Inset: a zoom of the PINK1 TMD, with the functionally important Tim17 acidic residues highlighted (human residue numbering: Tim17 D16, D77 and E127 (Fielden et al, 2023)). (**F**) Schematic of the N-terminal regions of PINK1 associated with the core TIM23-complex, including either ROMO1 (left) or PARL (right). Key features include the MTS, OMS and TMD of PINK1 (as in Fig. 1), highlighting: the GLGLGL-motif, the 3 conserved glutamates (E), the PARL cleavage site (A/F), the N-terminal truncation sites 50, 91 and 150 (see below (**G**, **H**)), conserved cysteines and arginines (2Cs and 2Rs) described later (Fig. 5) and I111 (see later Fig. 7). In the PARL-associated complex the rhomboid’s two β-strands are shown augmented by a third (GLGLGL of PINK1). Key interactions of the 3Es are shown with ROMO1 and Tim17 (left): including arginines (R) of ROMO1 and Tim17, as well as a glutamate (E) of Tim17, via an intra-molecular contact with a PINK1 lysine (K) (see also (**E**), and later Fig. 6E). In the complex associated with PARL (right) the 3Es are shown interacting with arginine (R) and asparagine (N) residues of PARL, as well an intra-molecular interaction with lysine (K), and with the head group of a phospholipid (see also later Fig. 5E). The three asterisks mark the locations of the 3 pep86 insertion sites, respectively of **L**DD, D**L**D and DD**L** (Figs. 1C and 2B). (**G**) *MitoLuc* trace for the import of the PINK1$\triangle$(1–50) and PINK1$\triangle$(1–91) truncated precursors into the matrix compared to the full-length (FL) protein. Normalised amplitudes were calculated at the point of plateau and plotted. Data shown represent *N* = 5 biological repeats, error bars show SEM. A one-way ANOVA with Tukey’s post hoc test was used to determine the significance of the normalised amplitudes (*P* values = 0.0029, 0.0027 and 0.9780). (**H**) *MitoLuc* trace for import of PINK1$\triangle$(1–150) into the matrix relative to the FL precursor. Normalised amplitudes are plotted. Data shown represent *N* = 3 biological repeats, error bars show SEM. A paired *t* test was used to determine significance of the normalised amplitudes (*P* value < 0.0001). Source data are available online for this figure.

Initially, an AlphaFold model of the PINK1-PARL complex was built, with the highest-scoring pose shown in Fig. 4A. Each chain of the model is in good agreement with experimentally determined structures. The kinase domain of the PINK1 model aligns well with the resolvable region of *Pediculus humanus corporis* PINK1 (residues 147–574) (Schubert et al, 2017), with an RMSD of 0.11 nm (Appendix Fig. S7a). Though note that the PINK1 structure shown here is unlikely to be its physiological state in this location (in association with PARL). In the IMS it is likely be in an unfolded conformation during import or cleavage/retro-translocation. The PARL chain of the model compares well with the *E. coli* rhomboid GlpG (Vinothkumar et al, 2010) (Appendix Fig. S7a), with slightly higher structural variability than the PINK1 chain (RMSD 0.64 nm), expected due to the evolutionary distance between human PARL and *E. coli* GlpG. Accordingly, the predicted Local Distance Difference Test (pLDDT) for the AlphaFold model is generally favourable, at ca. 80–90 for most of each chain Appendix Fig. S7b. The pTM score across the complex is respectable at 0.63.

In general, the predicted aligned error (PAE) score between the PINK1 and PARL chains in the complex is low. Accordingly, the ipTM is moderately low at 0.5. However, a notable high scoring PAE region can be seen in the graph (Appendix Fig. S7c, arrow), indicating a more confident region of the model. Analysis with ipSAE (Dunbrack, 2025) using PAE and distance cutoffs of 10, gives a higher confidence score for this lower PAE region, with ipSAE_d0chn = 0.62 (Appendix Fig. S7d). Thus, although the overall interface is predicted with low confidence, there is a region determined with comparatively high certainty. At the centre of this region is a small 3-stranded β-sheet formed between PINK1 and PARL (Fig. 4A). One strand of the β-sheet is formed from a section of the TMD region of PINK1 (sequence GLGLGL) between the cleavage site and three highly conserved acidic residues (Figs. 1C and 4A), while the other two strands are contributed by PARL.

Thus, in the presence of PARL, the PINK1 TMD spans the membrane as an α/β-hybrid. Interestingly, in the AlphaFold model of PINK1 alone from the EBI database (Varadi et al, 2021) (Appendix Fig. S7e) and also the structure of PINK1 bound to the TOM40-complex/VDAC at the outer membrane (Callegari et al, 2025), the TMD including the GLGLGL-motif is entirely α-helical, suggesting a localised unwinding induced by PARL. Notably, the α/β-hybrid arrangement brings the PINK1 cleavage site into proximity of the PARL protease active site (Fig. 4A: S277/H335; see also below). This configuration shows strong similarities with other intramembrane protease:substrate complexes; notably the structure of γ-secretase bound to amyloid precursor protein (APP) and to Notch (Yang et al, 2019; Zhou et al, 2019), including the proximity of the active site to the substrate, and the formation of the 3-stranded β-sheet (Fig. 4B,C). This supports the designation of this model as a plausible pre-cleavage PINK1/PARL interaction.

### Structural modelling identifies potential alternative protein channels at the interfaces between the TIM23-core-complex and either ROMO1 or PARL

To investigate the structure of PINK1 in association with the mitochondrial import machinery, we augmented the AlphaFold PINK1-PARL model to also incorporate the core TIM23-complex (Tim17-23-44) (Fig. 4D). This was achieved by first creating an AlphaFold-model of Tim17-23-44-PARL. This model had solid AlphaFold scores of pTM=0.66 and ipTM=0.65. The pLDDT was high for the whole complex (Appendix Fig. S8a), except for the N-terminal region of Tim44, which we excluded from downstream analyses. There was a reasonable confidence between PARL with its neighbour in the complex, Tim17, with much of the interface having a moderate PAE score of ~10 Å, and with a d0chn ipSAE of 0.43 (Appendix Fig. S8a). However, we note that the scores are lower than those observed between Tim17 and Tim23, potentially because Tim17 and PARL are more loosely associated. Therefore, caution is warranted when interpreting the precise interface between these proteins. The Tim17-23-44 arrangement closely resembles the published cryo-EM structure of the yeast complex (Appendix Fig. S8b (Sim et al, 2023)), with an RMSD of 0.15 nm between them.

The PINK1-PARL and Tim17-23-44-PARL models were then combined by overlaying the PARL subunits of the two complexes to create a predicted structure of Tim17-23-44-PARL-PINK1 (with no steric clashes). The resulting model reveals formation of a putative protein channel between Tim17 and PARL situated immediately adjacent to the rhomboid’s proteolytic active site (Fig. 4D: S277 and H335). This channel closely resembles the proposed protein pathway formed between Tim17 and Mgr2 (Appendix Fig. S8c). Here, the TMD of PINK1 is incorporated into the PARL active site, adjacent to, but not within, the described Tim17 protein channel. This channel is also evident in the model of the Tim17-23-44-PARL complex, produced without PINK1 (Appendix Fig. S8d). Note that since the larger complexes with PINK1, the TIM23-core and PARL (Fig. 4D) or ROMO1 (Fig. 4E, below) were each composites of 2 AlphaFold models, the PAE scores were not available for them as a whole.

In the yeast Tim17-23-44 cryo-EM study, the authors used AlphaFold2 to predict a protein channel between Tim17 and Mgr2 (Sim et al, 2023), which we were able to reconstruct in AlphaFold2 (Appendix Fig. S8c; pTM=0.69, ipTM=0.74). For the human counterparts, it was also possible to model the Tim17-23-44 complex with ROMO1, the homologue of Mgr2 (Richter et al, 2019; Ieva et al, 2014) (Appendix Fig. S8c; pTM=0.79, ipTM=0.76). This model is in agreement with a recent study, which also showed that the ROMO1 interaction can be formed by both orthologs of Tim17 (TIMM17A and TIMM17B) (Maruszczak et al, 2024).

The position of Mgr2 and ROMO1, respectively, in the yeast and human complexes, overlay with our predicted PARL binding site within the complex (Appendix Fig. S8c,d), suggesting there is a mutual exclusivity between Mgr2/ROMO1 and PARL. Based on the corresponding placement of ROMO1 and PARL in the TIM23-complex, we modelled the N-terminal domain of PINK1 into the Tim17-23-ROMO1 complex (Fig. 4E). Here, the PINK1’s TMD region is incorporated into the protein channel at the interface between Tim17 and ROMO1, positioned directly next to 3 acidic residues, known to be important for protein import (Fig. 4E) (Fielden et al, 2023). Interestingly, the PINK1 TMD is modelled as an α-helix rather than the α/β-hybrid seen in the complex with PARL.

Taken together, the different models suggest that ROMO1 promotes α-helical formation of the TMD suitable for matrix import, while PARL favours the α/β-hybrid conformation as a precursor to proteolysis (Fig. 4D–F). In the former situation, ROMO1 restrains the TMD within the protein channel for translocation across the inner membrane (Fig. 4E). And in the latter (Fig. 4D), PARL allows the lateral passage of the TMD away from the channel and into the proteolytic active site.

Note that, as we intended, the structural models above confirm that the pep86 insertion sites (Fig. 1C) are far away from the critical N-terminal regions and therefore unlikely to affect cleavage or matrix-import (Fig. 4F, asterisks).

### Mitochondrial matrix import requires the TMD of PINK1

The AlphaFold models highlight the potential importance of the N-terminal region of PINK1 for inner membrane association with both PARL and the TIM23-complex. The latter interaction predicts this region to be critical for matrix import. To examine this further, we constructed a series of successive N-terminal truncations of the PINK1 DDL variant for use in the *MitoLuc* assay: Δ1–50 (removal of the canonical MTS), Δ1–91 (removal of the MTS and OMS) and Δ1–150 (removal of the entire N-terminal region (MTS, OMS and TMD) retaining only the kinase domain) (Figs. 1A,C and 4F). Again, minor variations in the modified proteins’ interaction with 11S and their luminescent yield were monitored (Appendix Fig. S9) and used to scale the import analysis accordingly.

The results show that import yield into the matrix is impaired (by ~60%) in the Δ1–50 and Δ1–91 constructs (Fig. 4g), but only fully abolished in the Δ1–150 construct (Fig. 4H). Therefore, only the region between 91 and 150, including the TMD (containing PARL cleavage site) and an N-terminal α-helical extension (NTE) up to the kinase domain (Kakade et al, 2022), but not the OMS, is necessary and sufficient for association with the TIM23-complex and import. This is in excellent agreement with the AlphaFold models, which identify this region as being the most intimately associated with the Tim17-23-44 model complexes, bound by either ROMO1 or PARL. These results demonstrate a non-essential role for the MTS in the refinement of the targeting and translocation process. This is not particularly surprising as lots of mitochondrial proteins do not possess a cleavable N-terminal pre-sequence (Bykov et al, 2022). In those cases, the targeting information needs to be incorporated into the mature regions of the precursor, such as here in the TMD of PINK1.

### Molecular dynamics simulations identify stable interactions of PINK1’s TMD and a hydrated protein channel, potentially critical for inner membrane entry and cleavage

**Figure 5:**
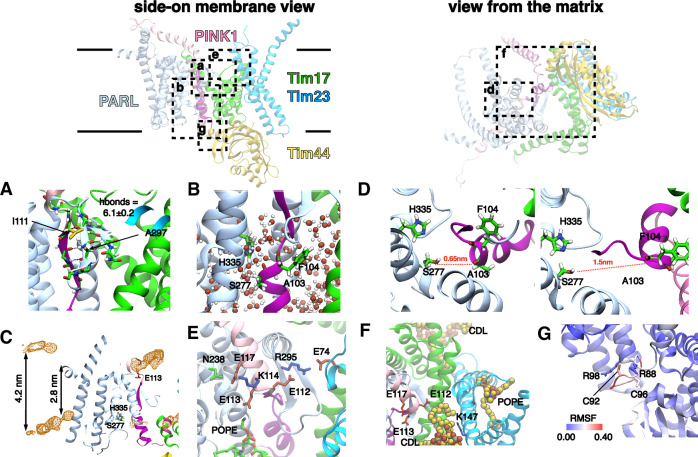
Molecular dynamics (MD) analysis of the PINK1–PARL–Tim17–Tim23–Tim44 complex. Upper panels: views of the PINK1-PARL-Tim17-Tim23-Tim44 complex from the side (left; lines indicating the position of the bilayer) and view from the matrix (right) with PINK1 truncated after residue 150 for clarity. Boxes are drawn for the orientation of (**A**–**G**). (**A**) View of the β-sheet formed by one β-strand of the PINK1 TMD and two from PARL post-MD simulation. The backbone atoms of residues that contribute to the β-sheet are shown as sticks and coloured by atom type. Hydrogen bonds, as identified by VMD, are shown. The number of hydrogen bonds between the highlighted residues is denoted (mean and standard deviation), as computed using Gromacs h-bond over the final 200 ns of each simulation. The residues PINK1-I111 and PARL-A297, which interact with one another, are shown as yellow sticks. (**B**) View of the PARL active site, with catalytic residues (S277 and H335), and PINK1 cleavage site (A103/F104) post-simulation, showing that the site is highly solvated. (**C**) Showing the membrane thinning about the PARL protein. The density of all lipid phosphate atoms has been computed for a simulation fitted on PARL using the VMD VolMap tool and shown as an orange mesh. For clarity, the density map has been clipped to only show a slice through the membrane around PARL. The PARL active site catalytic residues and E113 (one of the 3 conserved Es) of PINK1 are shown. Approximate membrane thickness, taken from phosphate-phosphate distances are shown. (**D**) Snapshot of the PARL active site and PINK1 cleavage site showing a typical (left, 0.65 nm) or maximum (right, 1.5 nm) distance from the hydroxyl of the catalytic residue S277 to the A/F cleavage site backbone. (**E**) Highlighting the 3 conserved glutamates (E112, E113 and E117) of PINK1 and the interactions it makes with PARL (R295), Tim23 (E74), and a surrounding phospholipid (POPE). (**F**) Matrix view of the complex showing the most tightly bound lipids. The CDL at the top and POPE on the right side were previously seen in the yeast Tim17/Tim23 structure (Sim et al, 2023). The CDL at the bottom is unique to the PARL-containing complex, and bridges PINK1, PARL, and Tim23. The interaction of K147 with CDL is of particularly high affinity, being occupied in 94% of all MD frames (as assessed using the PyLipID package (Song et al, 2022)). (**G**) Root-mean-squared fluctuation (RMSF) analysis reveals the matrix loop containing Cys92 and Cys96 to be highly dynamic (units = nm).

To substantiate the AlphaFold modelling and explore the dynamics and interactions required for PINK1 cleavage, we carried out all-atom molecular dynamics (MD) simulations of the PINK1-Tim17-23-44-PARL complex within a model mitochondrial membrane. During the simulations, the complex was very stable, with an RMSD for the transmembrane complex (excluding the PINK1 kinase domain) that plateaus at 0.38 ± 0.02 nm across the five repeats (Appendix Fig. S10a). Crucially, the PINK1-PARL β-sheet was completely stable throughout the simulation; quantification of the hydrogen bonding revealed the average incorporation of approximately six backbone hydrogen bonds inter-connecting the three strands (Fig. 5A). Adjacent to this, the catalytic site of PARL and cleavage site of PINK1 are well solvated, with water molecules freely able to access and vacate the region (Fig. 5B). This is achieved in part through a constriction in the membrane around PARL (Fig. 5C).

To further validate the model of the TIM23-PARL-PINK1-cleavage complex (see also below), we used the simulation to predict the location of the proteolysis site of the TMD. From the molecular dynamics trajectories, we measured the distances between the backbone carbonyls of the polypeptide, along the entirety of the TMD, to the catalytic residues of PARL. The average distances at equilibrium were then plotted for the respective residues across the α/β-hybrid (Appendix Fig. S10b); wherein the minimum is centred on amino acids 103 and 104—*bingo!* (Deas et al, 2011). Repeated simulations showed that the distance between the catalytic site of PARL and the PINK1 cleavage site (~0.65 nm) was reproducible and stable; although interestingly, in one instance, it withdraws to about 1.5 nm (Fig. 5D; Appendix Fig. S10c).

Immediately following PINK1’s GLGLGL-motif of the PINK1/PARL β-sheet, there are 3 conserved glutamates known to be essential for normal PINK1 cleavage (Figs. 1C, 2B and 4F). In our model and simulations, one of them, E112, interacts with R295 of PARL, which in turn interacts with E74 of Tim23 (Fig. 5E), helping to stabilise the complex. The next glutamate, E113, consistently binds to a POPE lipid, forming a crucial part of a high-affinity lipid binding site (occupied for the entire simulation), and which contributes to the membrane constriction around PARL (Fig. 5C,E). Additional lipid binding sites were also noted in this vicinity, including cardiolipin (CDL), POPC and POPE, located exactly as those visualised in the yeast cryo-EM structure (PDB 8SCX (Sim et al, 2023)); while we identify an additional CDL binding site between K147 of Tim23 and E112 of the PINK1/PARL β-sheet (Fig. 5F). This suggests the CDL might be important for stabilising the PINK1/PARL/TIM23-complex. The third glutamate, E117, forms interactions with K114 of PINK1 and N238 of PARL (Fig. 5E), likely also to contribute to inter-subunit stability.

Of further interest in this region is I111 of PINK1, found between the GLGLGL-motif and the 3 glutamates. This forms a stable hydrophobic interaction with A297 of PARL in the middle strand of the β-sheet throughout the simulation (Fig. 5A). This interaction would be likely to stabilise the PINK1/PARL β-sheet. Of note, substitution of this residue to serine (I111S) is implicated in early-onset Parkinson’s disease (Sekine and Youle, 2018), suggesting a direct link of this region to a disease phenotype.

The simulated structure identifies an interesting consequence of the orientation of PINK1 within the PARL-Tim17-23-44 complex. A short loop between the TMD and OMS contains two cysteines (C92 and C96; the latter being more conserved), which poke into the matrix (Figs. 1C, 4F and 5G). Both are adjacent to arginine residues, which promote the conversion of thiols (–SH) to thiolates (–S^−^), and thereby enhance their sensitivity to oxidation (Xiao et al, 2020). Therefore, they might be there for ROS sensing to monitor redox stress. In our simulations, these cysteines are very dynamic, which would fit this role (Fig. 5G; Appendix Fig. S10d).

Critically, the simulations show that the interface between Tim17 and PARL is predicted to be full of water molecules (Appendix Fig. S11), and therefore ideally suited for protein translocation. In this instance, for the entry of PINK1 into the membrane for proteolysis, as well as for the retro-translocation of the cleavage product.

### Molecular dynamics simulations identify a hydrated protein channel at the Tim17/ROMO1 interface stably occupied by the TMD of PINK1 for matrix-entry

**Figure 6:**
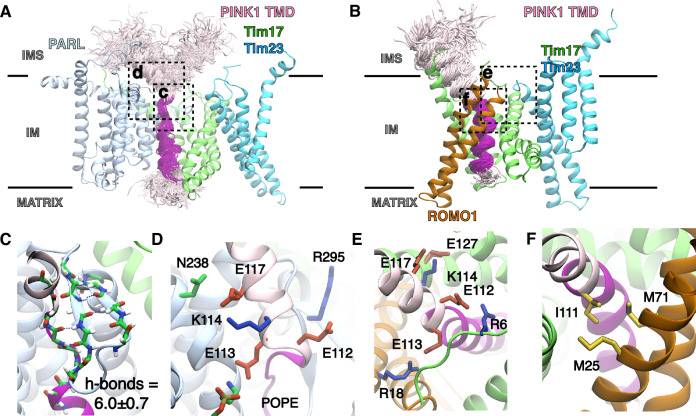
Molecular dynamics (MD) simulations of the TMD of PINK1 in the PARL and ROMO1 containing TIM23-core-complexes. Upper panels: views of the MD simulations of the PARL-Tim17-Tim23 (**A**) and ROMO1-Tim17-Tim23 (**B**) complexes, with the PINK1 TMD (purple), respectively, in the proteolytic cleavage site (**A**), or the protein channel (**B**), as positioned by AlphaFold2. The lines indicate the position of the bilayer. Multiple frames of the PINK1 TMD are overlaid to reveal the restricted dynamics of this polypeptide across the simulations. Orientation boxes are drawn for (**C**–**F**). (**C**) view of the β-sheet formed by the PINK1 TMD and PARL post MD simulation. As in Fig. 5A (simulated with full-length PINK1), the backbone of the β-sheet is shown as sticks with hydrogen bonds shown as dashed lines and the computed average hydrogen bond number is shown. (**D**) Key interactions between the three conserved glutamates of PINK1 and PARL are preserved in the truncated TMD simulations (compared to Fig. 5E). (**E**) View of the ROMO1-Tim17-Tim23-complex highlighting the interactions of the three conserved glutamates (see also Fig. 4F). (**F**) Highlighted interactions between I111 of PINK1 and ROMO1 preserved during the MD simulations.

We next assessed the nature of the PINK1 TMD within the ROMO1-bound import complex. To allow longer simulations, we modelled only the TMD of PINK1 into the Tim17-23-ROMO1 complex (Table 1). To verify that a short region of PINK1 is stable, we also truncated PINK1 in the pre-cleavage complex (as above with the Tim17-23-44-PARL complex and with Tim44 removed) and ran simulations in parallel (Fig. 6A,B). The data show that the truncated PINK1 TMD is stable within each complex: higher dynamics are seen at the PINK1 termini (expected due to their artificial trimming), but the central TMD-region is very stable.

In the PARL-containing complex, the PINK1/PARL β-sheet remains as stable as in the simulations with full-length PINK1 (Fig. 5A), again with ~6 hydrogen bonds consistently formed between the three strands (Fig. 6C). The RMSD for this central region of PINK1 in relation to the whole complex is 0.37 ± 0.09 nm. PINK1 is even more stable in the ROMO1-containing complex, with a RMSD of 0.20 ± 0.02 nm. These simulations highlight the very plausible existence of alternative protein-conducting channels formed between the core TIM23 complex, specifically Tim17, and either PARL or ROMO1 (Fig. 6A,B).

The simulations also highlight the stability of PINK1’s TMD, as either an α-helix or α/β-hybrid, within a hydrated channel or proteolytic active site; a consequence of the TMD not being particularly hydrophobic (Fig. 1C). It seems likely that this relatively hydrophilic TMD cannot span the bilayer alone. Rather, its transmembrane status is dependent on the association of other membrane proteins, which bring about membrane constriction and the infiltration of water.

The simulations of the respective complexes bound to PARL or ROMO1 illustrate compelling alternative structural views concerning the fate of PINK1: respectively, intermediates prior to either cleavage or matrix import. In the former, precisely consistent with the simulation containing full-length PINK1 (Fig. 5E), the three conserved glutamates form stabilising interactions at the PINK1-PARL interface: E112, salt-bridges to R295 of PARL, E113 binds to a POPE lipid, and E117 interacts with K114 of PINK1 and N238 of PARL (Fig. 6D). In the ROMO1 import complex, these PINK1 residues are predicted to also form important interactions at the entrance of the protein channel: E112 with R6 of Tim17, E113 with R18 of ROMO1, and E117 with E127 of Tim17 via K114 of PINK1 (Fig. 6E). Note that the latter network involves a residue known to be important in protein import (Tim17-E127) (Fielden et al, 2023). Moreover, the yeast homologue of ROMO1 (Mgr2) has been shown to associate with negatively charged residues flanking TMDs undergoing sorting (Ieva et al, 2014), consistent with the arrangement predicted here for PINK1.

Another residue in this region of the TMD, I111; immediately prior to the three key glutamates and relevant to Parkinson’s disease, seems also to form important contacts in the model of the matrix-import complex (Fig. 6F), as noted for the cleavage complex (Fig. 5A). In this case, stable hydrophobic interactions with a pair of methionine residues in ROMO1 at the entrance to the protein channel. Potentially, then I111 (like the 3 glutamates) contributes to the formation and stability of the protein channel for matrix import as well as PINK1’s interaction with PARL.

Taken all together, it is clear that the PINK1 TMD and the residues that immediately follow it, including I111 and the 3 conserved glutamates, are critical for the stabilisation of both complexes (Figs. 1C, 4F, 5A,E,F and 6D–F). Their contrasting interactions dictated by the presence of PARL or ROMO1 could potentially determine the structure of the TMD, and thereby direct PINK1 for cleavage or matrix import as required.

### Validation of the alternative structural models designated for PINK1 cleavage and matrix import

The modelled structures and simulations predict that the TIM23 core complex undergoes mutually exclusive associations with different accessory proteins for the conferral of different functions. In respect of PINK1, either a channel into the inner membrane for TMD cleavage (by PARL), or a channel into the matrix (ROMO1). The models appear credible because they recapitulate known structures of their constituents. A notable exception is the structural prediction of the PINK1 TMD bound to PARL, which rejects all of the experimental structures of PINK1, which are α-helical, in favour of a previously unseen configuration (α/β-hybrid). This structural transition is known to be compatible with intra-TMD proteolysis (Fig. 4B,C).

Further assurances of the credentials of our models arise, as noted above, from simulations which correctly predict the location of known phospholipid binding sites on the TIM23-core-complex (Sim et al, 2023). Moreover, the models also generate anticipated structural features; the constricted membrane and a water-filled channel leading to the protease active site (TIM23-PARL complex) as well as across the inner membrane compatible for polypeptide translocation to the matrix (TIM23-ROMO1 complex).

The identification of stabilising interactions involving key residues at the C-terminal/ IMS end of PINK1’s TMD (the 3 glutamates and I111, mentioned above), with both PARL (Figs. 5A,E and 6D) and ROMO1 (Fig. 6E,F), predicts that this region is important for both PINK1-cleavage and matrix-import. Indeed, the variant with all 3 glutamates substituted with alanines (3EA) and the variant associated with Parkinson’s disease (I111S) are both less prone to proteolysis by PARL (Sekine et al, 2019; Meissner et al, 2015). To test the prediction that this region of the TMD is also critical for matrix import, we implemented the *MitoLuc* assay to see how the same variants were affected.

**Figure 7:**
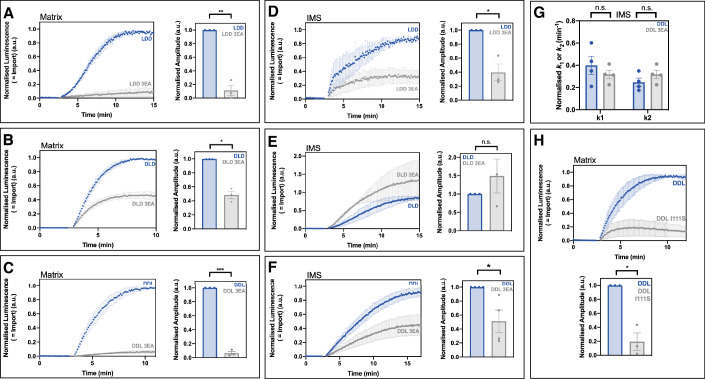
The three conserved glutamates and I111S of PINK1 are critical for matrix import. Luminescence traces monitoring import of PINK1 versus a variant wherein the three conserved glutamates are substituted with alanine (3EA). Data shown for entry of PINK1 into the matrix for the constructs LDD (*P* value = 0.0077) (**A**), DLD (*P* value = 0.0108) (**B**) and DDL (*P* value = 0.0007) (**C**); repeated for entry into the IMS (**D**–**F**) (*P* values = 0.0374, 0.4011, and 0.0223. (**G**) Comparison of the rate constants determined by fitting the *MitoLuc* data for IMS import (PINK1 DDL and PINK1(3EA) DDL) to a two-step kinetic model (as in Fig. 2E). A one-way ANOVA with Tukey’s post hoc test was used to determine the significance of the differences of k_1_ and k_2_ (*P* values = 0.1736 and 0.9036). (**H**) *MitoLuc* matrix import trace and associated amplitude data for the PD-linked PINK1 variant I111S. Error bars show SEM of the data (*N* = 3 biological repeats), with each biological repeat calculated from three technical repeats. A paired *t* test was used to compare the normalised amplitude data (*P *value = 0.0230). Source data are available online for this figure.

Once again, the 11S activation properties of the two variants were measured (Appendix Figs. S12 and S13) and used to scale the luminescent readout corresponding to import. The analysis of the 3EA variant highlights their expected loss of import into the matrix. Only tiny amounts of the N- (LDD) and C-terminal (DDL) sections of PINK1 entered the matrix, while the central section (DLD), just prior to the kinase domain, was affected to a lesser degree, being reduced by about half (Fig. 7A–C). In respect of IMS import, the three key glutamates were not essential for full entry of PINK1. Levels of PINK1-3EA in the IMS were reduced, but to a much lesser degree, mirroring the pattern for matrix entry; the N- and C-terminal reporters were reduced by ~50%, while the central section was unaffected (Fig. 7D–F). Note that the positive luminescent response of PINK1-3EA with IMS-targeted 11S is consistent with its intended localisation (incorrect matrix targeting would not have elicited a signal).

Further analysis of IMS import showed that the two rate-determining steps of transport across the outer membrane were unaffected by the 3EA amino acid substitutions (Fig. 7G). This shows that reduced matrix import is a not a consequence of altered transport across the TOM-complex, but must be a result of diminished transport through the TIM23-complex. The lower yield of IMS entry is presumably a result of restricted transport further downstream, through the inner membrane. Thus, both cleavage (Sekine et al, 2019) and transport across the inner membrane (but not the outer membrane) of the 3EA variant are reduced.

Based on the AlphaFold model, the Parkinson’s-associated amino acid substitution I111S should also affect import (Fig. 6F), which turned out to be the case. Complete matrix entry, measured by import of the C-terminus (PINK1-DDL), is reduced (Fig. 7H).

Therefore, the experiments performed here, and elsewhere (Meissner et al, 2015; Sekine et al, 2019), support our hypothesis that the AlphaFold models relate to intermediates of PINK1 formed prior to proteolysis (with the TIM23-complex and PARL) and transport into the matrix (with TIM23-complex and ROMO1).

### PINK1’s fate for cleavage or matrix import is regulated by the structural plasticity of its TMD

The structural modelling predicts that the different TIM23-complexes established prior to either PINK1 cleavage or matrix import are associated with the TMD configured respectively as an α/β-hybrid or an α-helix. To substantiate these alternative destinies of PINK1, and their reliance on TMD-conformation, additional experiments were established to monitor the interplay and dependencies of PINK1 cleavage and matrix import.

Initially, we simply measured the cleavage of endogenous PINK1 in HEK293T cells. If PINK1 is subject to a responsive dynamic equilibrium between cleavage and import, then inhibition of the canonical import pathway should enhance cleavage. The inhibitor MB20 has been previously shown to destabilise the interaction of the TIM23 protein channel with the PAM-motor complex and thereby inhibit precursor entry into the matrix (Cheung, 2017). We surmised that blocking this specific import route would bring about the redirection of PINK1 to the TIM23-PARL complex.

**Figure 8:**
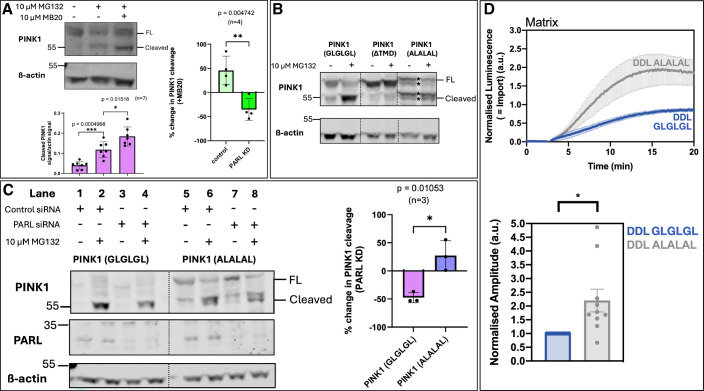
Propensity for PINK1 cleavage *versus* import and dependence on the structure of its transmembrane domain (TMD). (**A**) Western blot visualising endogenous full-length and cleaved PINK1 within HEK293T cell lysates treated with MG132 and MB20. PINK1 cleavage with the addition of MG132, or MG132 + MB20 quantified below. Error bars show SEM of the data (*N* = 7 biological repeats). This experiment was repeated following PARL knockdown in cells, and a change in PINK1 cleavage upon MB20 addition was quantified for *N* = 4 biological repeats (right). A two-tailed unpaired *t* test was used to confirm significance in both cases. (**B**) Western blot detecting full-length and cleaved PINK1 within lysates of cells exogenously producing the indicated PINK1V5 construct. Asterisks indicate PARL-independent (see (**C**)) cleavage products. (**C**) Full-length and cleaved PINK1 visualised by Western blotting of lysates of cells producing either PINK1(GLGLGL) or PINK1(ALALAL) in the presence and absence of MG132, in addition to the presence of PARL siRNA (or an siRNA control). Change in PINK1 cleavage with PARL knockdown quantified for each construct (right). Error bars show SEM of the data (*N* = 3 biological repeats). A two-tailed unpaired *t* test was used to confirm significance. All blots were produced using the Novus Biologicals PINK1 antibody. (**B**, **C**) Dotted lines indicate where Western blot images have been cropped and spliced together, in order to compare lanes directly. (**D**) *MitoLuc* analysis of mitochondrial import into the matrix of PINK1 DDL, compared to the equivalent construct with GLGLGL substituted for ALALAL. Normalised amplitudes were calculated and plotted. Error bars represent SEM and data an *N* = 10 biological repeats where three technical repeats were performed for each biological repeat. A paired *t* test was used to confirm the significance of the amplitude data (*P* value = 0.0175). Source data are available online for this figure.

The product of PINK1 cleavage by PARL as expected is subject to digestion by the proteasome. This is demonstrated by its accumulation in the presence of the proteasome inhibitor MG132 (Fig. 8A, cleaved); consistent with its destination for retro-translocation and cytosolic degradation (Fig. 1B). The results show that this cleavage product is indeed enhanced upon addition of MB20 (Fig. 8A, left panel). A further analysis of this MB20 enhanced cleavage product, following depletion of PARL by siRNA (Appendix Fig. S14), confirmed the requirement of the rhomboid protease (Fig. 8A, right panel; Appendix Fig. S15). Therefore, as predicted, blocking matrix import diverts PINK1 towards PARL-dependent cleavage, retro-translocation and proteasomal degradation.

Next, we decided to examine the prediction that PINK1’s fate depends on the conformation of its TMD. We reasoned that destabilisation of the β-strand of the α/β-hybrid-TMD (stabilisation of the α-helix) should diminish the interaction with PARL and thereby interfere with cleavage. To do this, we created a PINK1 variant wherein the helix-breaking glycine residues of the GLGLGL-motif (Figs. 1A,C and 4F) were substituted with alanines—residues that have the highest propensity for promoting α-helix formation, without changing side chains dramatically (Allen et al, 2022; Kyte and Doolittle, 1982; Deléage and Roux, 1987; Zimmerman et al, 1968).

The resulting PINK1(ALALAL) variant was then subject to cleavage assays alongside native PINK1(GLGLGL) and a version wherein the entire TMD had been removed PINK1(ΔTMD)—all being exogenously produced—(Fig. 8B). In this case, the variants were compared, as above, in the presence and absence of the proteasome inhibitor MG132. As expected, the cleavage product of PINK1(GLGLGL) was subject to proteasomal degradation (protection by MG132), and removal of the TMD prevented proteolysis (Fig. 8B). However, contrary to our expectations, PINK1(ALALAL) was still subject to proteolysis. Although its apparent sensitivity to the proteasome differed (Fig. 8B, ±MG132) and the cleavage pattern was somewhat different to the native version (Fig. 8B, asterisks). The absence of these products from the PINK1(ΔTMD) control (Fig. 8B) suggests they are a result of cleavage of the TMD.

To explore the cleavage of the α-helical TMD further, we looked at its dependence on PARL. The results showed that while cleavage of the native TMD-containing GLGLGL was dependent on the rhomboid (Fig. 8C, lane 4 versus 2; see quantification right panel), cleavage of the version with the stabilised α-helix PINK1(ALALAL) was not (Fig. 8C, lane 8 versus 6; quantification right panel). Evidently then, the α-helical form of PINK1’s TMD shies away from PARL to become the subject of another protease, perhaps one that is a matrix resident.

Given that the stabilisation of PINK1’s TMD as an α-helix prohibits its interaction with PARL, it follows that it should enhance matrix import. As anticipated, the PINK1(ALALAL) variant demonstrates higher import compared to the native PINK1(GLGLGL) (Fig. 8D; Appendix Fig. S16). This is consistent with the idea that the configuration of the TMD as an α-helix or an α/β-hybrid regulates PINK1’s respective propensity for matrix import or cleavage and retro-translocation.

## Discussion

The successful production of full-length human PINK1 combined with our in-cell *MitoLuc* assay and molecular modelling and simulations have inspired a re-evaluation of PINK1’s activity.

### An extended model for the mitochondrial import behaviour of PINK1 (Fig. 9) builds on the canonical version (Fig. 1B)

**Figure 9:**
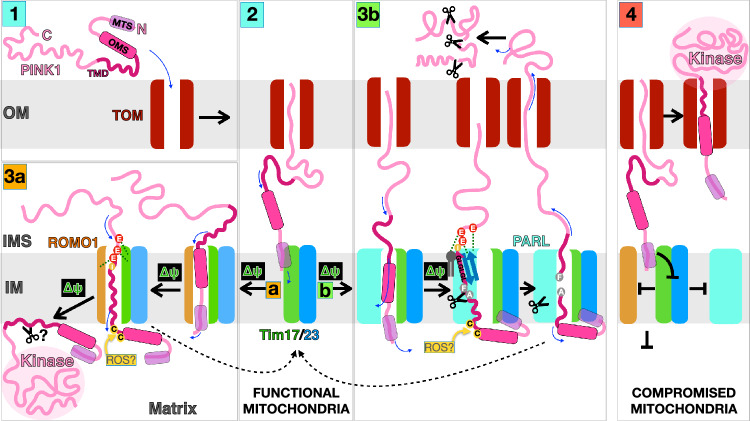
A revised model for mitochondrial import of PINK1. Schematic overview of the different destinations of PINK1; see Discussion for further details. Key elements of PINK1 are labelled in Box 1 (as in Fig. 1B); mitochondrial targeting sequence (MTS), outer-membrane targeting sequence (OMS), transmembrane domain (TMD), with consistent colouring throughout; blue arrows indicate the direction of protein translocation. According to the current model, PINK1 is transported through the TOM-complex into the IMS to make contact with the TIM23-complex (Box 1 → 2). In functional mitochondria, generating a membrane potential (Δψ), PINK1-Tim17-23 (as part of the larger TIM23-complex) recruits either ROMO1 (Box 3a) or PARL (Box 3b)—both completing a channel for translocation of the N-terminus across the membrane, driven by Δψ. In the former case, PINK1’s TMD forms an α-helix which can readily be transported (due to its weak hydrophobicity) into the matrix along with the rest of the protein, again driven by Δψ (Box 3a); whilst ROMO1 prevents the TMD from lateral release to thereby ensure transport across the membrane and into the matrix. In contrast, the interaction with PARL promotes transport towards the matrix and lateral entry of the TMD into the inner membrane about PARL (Box 3b). Formation of the α/β-hybrid promotes retraction of the TMD from the channel into the proteolytically active site for cleavage between phenylalanine (F) and alanine (A). The C-terminal cleavage product is then transported back towards the outer membrane, and the N-terminal fragment presumably released for degradation (though its destination has not been addressed). Retro-translocation of the C-terminal fragment back through the TOM-complex results in degradation by the proteosome. Following import or cleavage the respective dissociation of ROMO1 or PARL dismantles the vacant protein channels and returns the components for future rounds of precursor entry (dashed arrows). In the former, the entry of full-length PINK1 into the matrix could become exposed to resident proteases, as indicated. When mitochondria become compromised, the loss of Δψ means that PINK1’s N-terminus is not incorporated into the inner membrane and the full-length protein is then trapped at the outer membrane (Box 4), leading to dimerisation, kinase activation and Parkin recruitment. The three conserved glutamates (E) are shown making important, but distinct, interactions with both ROMO1 and PARL-associated complexes. Between the 3Es and the TMD is an isoleucine (I), which when substituted with serine (I111S) brings about early-onset Parkinson’s disease. Reactive oxygen species (ROS) may influence the fate of PINK1 for matrix import (Box 3a), cleavage (Box 3b) or outer-membrane activation (Box 4) by the oxidation of activated thiols at the N-terminal end of the TMD in the matrix.

It highlights the importance of the N-terminal region of PINK1 (including the TMD) for import, and our determination that precursor passage into the IMS occurs in 2 distinct steps, most likely: (1) association with the TOM-complex (Fig. 9, Box 1), and (2) transport across the outer membrane (Fig. 9, Box 2), the latter being largely complete before engagement with the TIM23-complex (Ford et al, 2022). In functional mitochondria, the incursion of PINK1 into the inner membrane, requiring the membrane potential (Jin et al, 2010), can proceed at the interface between Tim17 and either the Mgr2 homologue ROMO1 (Fig. 9, Box 2 → 3a) or PARL (Fig. 9, Box 2 → 3b).

The former of these eventualities brings about matrix import of full-length PINK1 by the classical TIM23 pathway; though we recognise that alternatives may take the place of ROMO1, given that it is dispensable (see below). Either way, once in the matrix the α-helical TMD might be subject to cleavage, potentially by the mystery protease alluded to in the results (Fig. 8B, C). In the case of the latter pathway, active engagement of PINK1 with PARL is promoted by inter-protein β-augmentation, and further stabilised by salt bridges of the conserved 3 glutamates of PINK1. The ensuing cleavage brings about retro-translocation of the truncated C-terminal fragment, followed by proteasomal degradation (Fig. 9, Box 3b).

The incorporation of PINK1 into a proteinaceous environment, within either the pre-matrix import or pre-cleavage complex, allows the weakly hydrophobic TMD to span the inner membrane. Furthermore, the TMD’s amphipathic characteristic is presumably important to promote its dynamic behaviour; enabling, as required, either transport through the Tim17-ROMO1 channel into the matrix (Fig. 9, Box 3a), or lateral entry into the active site of PARL (Fig. 9, Box 3b). In the former, the lack of TMD hydrophobicity, along with channel encapsulation aided by ROMO1, would favour passage across the inner membrane, rather than sideways partitioning into the bilayer.

In compromised mitochondria, when Δψ is diminished (e.g. artificially with CCCP) PINK1 fails to fully penetrate the inner membrane, and therefore is neither transported to the matrix, nor cleaved by PARL; the latter as previously noted (Jin et al, 2010). Instead, the full-length protein becomes stabilised at the outer membrane, which leads to mitophagy (Fig. 9, Box 4). This eventuality is also promoted by the replacement of the 3 key negative charges (3EA) and by the I111S variant linked to Parkinson’s disease, due to the destabilisation of the interaction between PINK1 and PARL. Similarly, these variants affect the interaction with Tim17/ROMO1 (Fig. 9, Box 3a), and thereby also diminish PINK1 import.

This extended model relies on the reversible association of the TIM23-complex with either PARL or ROMO1 to enable alternative channel compositions for the respective different activities. Thus, at the end of the import/cleavage process, the accessory components dissociate (Fig. 9, dashed black arrows) so as not to leave a depolarising vacant channel in the inner membrane, and for the initiation of another round of precursor entry. It follows that recruitment of various accessories, including as yet unknown participants, could feature to assemble alternative channels to confer similar and alternative activities and precursor fates (see below). A recent structural analysis of the TIM23-complex suggests that the reversible association of Mgr2 (the yeast ROMO1 homologue) is indeed a feature of the canonical precursor import process (Yang et al, 2025).

### An alternative protein channel?

A picture is emerging of a dynamic translocon capable of reversible and mutually exclusive associations with accessory factors for the formation of bespoke protein channels across the inner membrane. Mgr2/ROMO1 is the canonical example which, as previously described, complements the TIM23-complex to form the protein channel for precursor entry into the matrix (Yang et al, 2025; Maruszczak et al, 2024; Sim et al, 2023; Fielden et al, 2023). Given that both yeast and human Mgr2 homologues are dispensable, it seems likely that other factors can fulfil the same function.

This prediction that alternatives to Mgr2/ROMO1 can also complement Tim17 to form a protein channel seems to be true, at least as demonstrated by the model and simulation of the TIM23-PARL complex. The channel is superficially similar to the one formed for translocation into the matrix, though in this case it is unoccupied. It seems likely that the channel between Tim17 and PARL is required for the entry of PINK1 into the rhomboid active site and movement back towards the outer membrane, rather than for transport into the matrix. The formation of a protein channel at the interface with a rhomboid ‘half-channel’ has been noted before. The retro-translocation of proteins from the endoplasmic reticulum (ER) lumen back to the cytosol for ER-associated degradation (ERAD) is thought to occur between Hrd1 and the rhomboid-like Der1 (Wu et al, 2020). The similarity to what we propose here is striking.

Perhaps, there are a number of other membrane proteins (like PARL, but non-proteolytic) that are also able to encapsulate a protein channel, and thereby act, when required, as an alternative to Mgr2/ROMO1.

### Discrimination between alternative matrix and outer-membrane destinations of PINK1 and their consequences

Previous studies have shown that in healthy mitochondria PINK1 is degraded, while when compromised the kinase is activated at the outer membrane to bring about mitophagy (Fig. 1B). Though the documented ‘compromised’ state is very extreme (the addition of 10 μM of a potent ionophore CCCP). The results presented here suggest a more nuanced role, whereby in energised mitochondria PINK1 is either degraded or transported to the matrix (Fig. 9). As described, we propose these alternative fates are regulated by the interactions with either PARL or ROMO1, and the respective adoption of the GLGLGL-motif as a β-strand or α-helix. In the former, the TMD α/β-hybrid is characteristic of substrates of intramembrane proteases. Indeed, it is known that rhomboid specificity is dependent on helix-breaking residues within the TMD (Urban and Freeman, 2003). The similarity of the α/β-hybrid structure, its β-augmentation and the location of the cleavage site within another intramembrane proteases (APP/Notch with γ-secretase) is striking. However, the unique feature in PINK1 is its ability to also adopt an α-helical conformation.

If PINK1’s TMD is an inter-converting conformational switch, then what are the deciding factors for these alternatives, and their respective interactions with PARL and ROMO1? PINK1’s response to mitochondrial stress is unlikely to be black or white: distinguishing between fully functional mitochondria and membrane potential wipeout (similar to the effects of CCCP). Certainly, neurons of the brain will need to respond to only minor changes in bioenergetic fitness. Perhaps, PINK1’s redox and membrane potential sensing capabilities are more refined. Whilst catastrophic loss of membrane potential would lead to PINK1’s surface activation (Fig. 9, Box 4), a more subtle reduction in mitochondrial activity could affect PINK1’s fate for cleavage versus import to elicit an appropriate response. This could be brought about by a diminished (but not total loss of) membrane potential, and/or redox-stress and ROS. In the latter scenario, oxidation of the putative redox-sensor in PINK1 could help discriminate one pathway over the other (Fig. 9, ROS?). It has previously been shown that ROS can indeed influence PINK1’s trafficking behaviour (Xiao et al, 2017).

### Implications for the role of PINK1 in the regulation of mitochondrial function and quality control

The alternative destinies of PINK1, in response to various stimuli, suggest its involvement in mitophagy-independent regulation. This regulation will depend on control of the proportion of PINK1’s matrix delivery versus cleavage/retro-translocation/degradation, and ultimately the consequences of matrix kinase activity on mitochondrial structure and activity.

Though not widely recognised, matrix import of PINK1 has been reported previously. These observations include entry of the kinase domain for activation of Complex I (Pogson et al, 2014; Morais et al, 2014). This target alone suggests that PINK1’s presence in the matrix has a fundamental influence over mitochondrial activity. Complex I serves as the entry point for NADH into the electron transport chain, central to mitochondrial bioenergetics, metabolism and the generation of ROS. Therefore, with this lever alone, it seems that PINK1 could have a profound effect on mitochondrial activity. It will be interesting to find out what other targets and mitochondrial activities are at the behest of PINK1.

### Impact of PINK1 trafficking on health

Several mutations of *pink1* are implicated in early-onset Parkinson’s disease (Sekine and Youle, 2018). In many cases, presumably, through changes to its kinase activity at the outer membrane and improper regulation of mitophagy. The results presented here suggest that the explanation for some of the associated phenotypes may need to be reconsidered. Amino acid substitutions in the N-terminal region, particularly in and around the TMD, could also affect PINK1 matrix import as well as cleavage—as we show here for I111S. Other variants could affect the preference for interactions with ROMO1 or PARL, and thereby the balance of matrix import versus cleavage. Alternatively, substitutions could also affect the sensitivity to specific cues for import/cleavage (e.g. Δψ, ROS). Interestingly, variants affecting this putative redox-sensor of PINK1 (C92F and R98W) are also associated with early-onset Parkinson’s disease (Sekine and Youle, 2018; Truban et al, 2016).

Crucially, mutations affecting kinase activity will also have implications for the regulation of matrix activities. Therefore, there is a distinct possibility that the causes of some forms of familial PINK1-associated Parkinsonism are a consequence of unregulated mitochondrial function, rather than of quality control. One case in point, failure to activate Complex I when needed, would be problematic. Interestingly, Complex I defects are also associated with Parkinson’s disease (González-Rodríguez et al, 2021; Schapira et al, 1990), so the failure of its activation by PINK1 could be a contributory factor to its progression.

More work needs to be done to better understand the cues for PINK1 distribution and degradation, and their consequences, and how they are affected by the various *pink1* mutants. With this new framework, and further analysis, it is conceivable that new strategies could be developed for the treatment of specific familial forms of Parkinson’s disease. For instance, by controlling and rebalancing the alternative destinations of PINK1 to correct the mis-regulation of mitochondrial structure, function and quality control. One way to achieve this could be by small-molecule interventions that shift the conformational dynamics of PINK1’s TMD, and its interaction with either Tim17-ROMO1 (promoting matrix import) or Tim17-PARL (for degradation) as required.

## Methods

**Table:** Reagents and tools table

Reagent/resource	Reference or source	Identifier or catalogue number
**Experimental models**		
HEK293T cells	Abcam	
HEK293T PINK1 KO cells	Abcam	ab266393
HeLa cells	ThermoFisher Scientific	
BL21 (DE3) *E. coli*	Agilent	200131
XL-1 Blue *E. coli*	Agilent	200236
**Recombinant DNA**		
pCA528-His-SUMO	In-house	
pLVX	Takara Bio	632164
pXLG3-PX	In-house	
pBAD-Su9-EGFP-pep86-6xHis	In-house	
pLenti6-DEST	In-house	
pXLG3-eqPF670-P2A-Cox8a-11S	In-house	
pXLG3-eqPF670-P2A-Smac/DIABLO	In-house	
pTwistCMV-PINK1V5(GLGLGL)	Twist BioScience	
pTwistCMV-PINK1V5(∆TMD)	Twist BioScience	
pTwistCMV-PINK1V5(ALALAL)	Twist BioScience	
**Antibodies**		
ß-actin	Sigma-Aldrich	A2228
PINK1	Cell Signalling Technology	6946
V5	GeneTex	GTX42525
GFP	Sigma-Aldrich	G1544
HSP60	ThermoFisher Scientific	AB_2121466
Nanoluc	Promega	Gift
PINK1	Novus Biologicals	BC100-494
PARL	Abcam	Ab118554
Anti-mouse-HRP	Invitrogen	SA5-10276
Anti-mouse-DyLight 800	Invitrogen	SA5-10172
Anti-rabbit-HRP	Invitrogen	31462
Anti-rabbit-DyLight 680	Invitrogen	35569
**Oligonucleotides and other sequence-based reagents**		
Primers for cloning	Eurofins Genomics	
Dharmacon ON-TARGETplus^TM^ PARL siRNA pool	Horizon Discovery	L-021387-00-0020
Dharmacon ON-TARGETplus^TM^ Non-targeting control siRNA pool	Horizon Discovery	D-001810-10-20
**Chemicals, enzymes and other reagents**		
Q5 High Fidelity DNA Polymerase	New England Biolabs	M0491L
NEBuilder HiFi DNA Assembly Master Mix	New England Biolabs	E2621L
QIAquick PCR purification kit	Quiagen	28106×4
QIAprep Spin Miniprep Kit	Qiagen	27106×4
PureYield Plasmid Maxiprep System	Promega	A2393
T4 DNA ligase	New England Biolabs	M0202L
Mitochondrial Isolation Kit for Cultured Cells	Abcam	ab110170
Nano-Glo® Luciferase Assay System	Promega	N1120
4–12% BOLT gel	ThermoFisher Scientific	NW04125BOX
NuPAGE™ Bis-Tris Mini Protein Gels, 4–12%	ThermoFisher Scientific	NP0336PK2
PierceTM BCA Protein Assay Kit	ThermoFisher Scientific	A55864
MG132	Sigma-Aldrich	M7449
MB20	Custom synthesised	NewChem Technologies (now Sterling Pharma Solutions)
DharmaFECT1 transfection reagent	Horizon Discovery	T-2001-01
Lipofectamine 3000	ThermoFisher Scientific	L3000008
Lenti-X^TM^ concentrator	Takara Bio	631231
Seahorse XF Calibrant	Agilent	100840-000
IPTG	Sigma-Aldrich	I6758
Ni2+ Sepharose fast flow column	Cytiva	17057502
Tris/Glycine buffer	BioRad	610771
Imidazole	Sigma-Aldrich	I5513
HiTrap SP HP column	Cytiva	17115201
HisTrap FF column	Cytiva	17525501
HiTrap Q HP anion exchange column	Cytiva	17115401
DMSO	Sigma-Aldrich	472301
Urea	Sigma-Aldrich	56180
Guanidine-HCl	Sigma-Aldrich	G3272
RIPA Buffer	Sigma-Aldrich	R0278
cOmplete^TM^ EDTA-free Protease inhibitor cocktail	Roche	04693132001
PMSF	Sigma-Aldrich	93482
Proteinase K	Sigma-Aldrich	1.24568
Digitonin	Sigma-Aldrich	D141
CCCP	Sigma-Aldrich	C2759
Antimycin A	Sigma-Aldrich	A8674
Oligomycin	Sigma-Aldrich	O4876
FCCP	Sigma-Aldrich	C2920
Rotenone	Sigma-Aldrich	557368
High glucose DMEM	Gibco	11965-092
FBS	Gibco	10437-028
HBSS	Gibco	14185-052
Trypsin-EDTA	Gibco	25300-062
Sodium pyruvate	Gibco	11360070
**Software**		
SnapGene	GSL Biotech	
GraphPad Prism 7	GraphPad	
ProFit 7	QuantumSoft	
FIJI/ImageJ	NIH	
AlphaFold2 (ColabFold)	DeepMind/Google	
VMD 1.9.3	Humphrey et al, 1996	
Gromacs 2020.1	Abraham et al, 2015	
CHARMM-GUI	Lee et al, 2016	
PyLipID	Song et al, 2022	
PyMOL	Schrodinger	
Proteome Discoverer v2.4	ThermoFisher Scientific	
**Other**		
Seahorse XF Analyzer	Agilent	
Odyssey Fc Imaging System	LI-COR	
CLARIOstar Plus Plate Reader	BMG LabTech	
Olympus IX83 SpinSR Microscope	Olympus	

### Cloning

See Appendix Table S1 detailing all of the constructs assembled and deployed in this study. In silico cloning including plasmid, gene strand and primer development/modification was carried out on Snapgene software. The vector used for bacterial expression of PINK1 proteins was pCA528-His-SUMO. The mammalian constructs pLenti6-DEST PINK1V5 WT and pLenti6-DEST PINK1 V5-KD were gifts from Mark Cookson (Addgene plasmids #13319 and #13320; http://n2t.net/addgene:13319/; https://www.addgene.org/13320/; RRID:Addgene_13319/13320) (Beilina et al, 2005). WT (GLGLGL), ∆TMD and ALALAL PINK1V5 constructs used in cleavage assays were purchased in the pCMV vector from Twist BioScience. PINK1 cDNA was followed by a GSG linker prior to the V5 tag, encoding the amino acid sequence ‘GKPIPNPLLGLDST’. The region encoding the transmembrane domain of PINK1 (G94 to L110) was removed to create the ∆TMD construct. Codons ‘ggg’, ’ggg’ and ’ggc’ encoding amino acids G105, G107 and G109 respectively, were changed to ’gcg’, ’gcg’, ’gcc’ to produce the ALALAL construct. Gibson cloning was performed using NEBuilder HiFi DNA Assembly Master Mix (NEB) as per the manufacturer’s instructions. PCR reactions were performed using the Q5 High Fidelity DNA Polymerase (NEB), starting with 1 ng template DNA and using all other concentrations as indicated by the manufacturer. Subsequent PCR products were purified using the QIAquick PCR purification kit (Qiagen). Commercial NEB restriction enzymes were used for restriction digests, in which the reaction was typically carried out at 37 °C for 1 h. Subsequent ligations were performed at 16 °C overnight using the NEB T4 DNA ligase. The QIAprep Spin Miniprep Kit (Qiagen) and PureYield Plasmid Maxiprep System (Promega) were used for plasmid preparation. Sequencing of plasmids was performed by Eurofins Genomics. Transformations were carried out as indicated below for a variety of competent cells (BL21 (DE3), XL-1 blue and $\alpha$-select).

### BL21 (DE3) expression strain and transformation

In-house BL21 (DE3) chemically competent *E. coli* were originally sourced from NEB, prior to generation of in-house lab stocks and were used for expression of PINK1 LD variants. For transformation, 50 µL BL21 (DE3) bacteria were incubated with 10–50 ng DNA for 30 min on ice. Bacteria were then heat-shocked at 42 °C for 45 s followed by 3 min incubation on ice. In total, 750 µL SOC media was added to the bacteria that were subsequently recovered at 37 °C for 1 h. The transformation mixture was then plated on LB agar supplemented with either 50 µg/mL kanamycin or 100 µg/mL ampicillin.

### HEK 293T cell culture

HEK 293T cells were maintained in ventilated T-75 flasks in CO_2_ incubators supplying 5% CO_2_ with a maintenance temperature of 37 °C. Cells were kept in glucose media (High glucose DMEM (Gibco 11965-092), 10% FBS (Gibco, 10437-028) 1× penicillin streptomycin (P/S, Sigma)) and passaged at approximately 70% confluency. Passaging was carried out by first washing cells with 1× HBSS followed by detachment in 0.05% Trypsin-EDTA (1×) (Gibco, 25300-062). Glucose media was then added to deactivate trypsin after ~1–5 min and the cells were seeded at the appropriate density for the downstream experiment. For cleavage assays, cells were grown in 6-well plates to ~70% confluency before the addition of relevant drugs and harvesting. In all, 10 µM MG132 and 10 µM MB20 were added to the required wells and cells were incubated for 4 h before harvesting, as described.

### Transfection

Cells were grown to approximately 70% confluency and transfected with 1 µg DNA using Lipofectamine 3000 (ThermoFisher Scientific). Cells were subsequently incubated at 37 °C for the desired expression window, typically 48–72 h. For PARL knockdown experiments, Dharmacon ON-TARGETPlus^TM^ SMARTpool siRNAs targeting human PARL (55486) were used alongside a control, non-targeting siRNA pool. siRNA and DharmaFECT transfection reagent were added to cells at the time of seeding, such that cells were reverse-transfected with a final concentration of 100 nM siRNA. Cells were harvested 72 h after transfection. For experiments involving PARL knockdown and PINK1V5 overexpression, cells were transfected with 1 µg plasmid DNA 24 h after siRNA addition, and incubated for a further 48 h prior to harvesting.

### PINK1 LD series and variants purification

A 100 mL PINK1 LD variant-transformed BL21 (DE3) bacterial pre-culture supplemented with 50ug/mL kanamycin was grown overnight at 37 °C, shaking at 200 rpm. The following morning pre-culture was used to inoculate 4 L kanamycin-supplemented LB culture. Cells were allowed to grow at 37 °C for ~2 h until O.D. had reached ~0.6–0.8. Expression was then induced following the addition of 0.1 mM isopropyl ß-D-1-thiogalactopyranoside (IPTG) and subsequent growth at 37 °C for 3 h. Cells were harvested by centrifugation at 5000×*g* for 15 min (Sorvall LYNX 6000). Cell pellets were subsequently resuspended in TG buffer (50 mM Tris pH 8.0, 8 M guanidine-HCl, pH 8.0), left shaking on ice for 20 min and then sonicated at 100% amplitude for a cycle of 5 × 40 s on, 40 s off (Fisherbrand Model 120 Sonic Dismembrator). Cell lysates were clarified by centrifugation at 38,000 rpm for 45 min at 4 °C (Beckman Optima XPN-80) before the soluble fraction was loaded onto a chelating Ni^2+^ Sepharose fast flow column (Cytiva, 17057502) equilibrated in TG buffer. The column was then washed with TG + 30 mM imidazole and then TNU buffer (50 mM Tris, 500 mM NaCl, 6 M urea, pH 8.0) + 30 mM imidazole. Protein was eluted in TNU buffer + imidazole over a gradient of 1CV from 0 to 500 mM imidazole. Pooled elution fractions were diluted stepwise to 2 M urea in TN buffer (50 mM Tris, 500 mM NaCl, pH 8.0), BME added to 1 mM and then cleavage of the SUMO tag performed by the addition of in-house purified Ulp1 for 1 h at room temperature. The cleaved sample was then diluted in TNU to dilute the imidazole concentration to 30 mM prior to reloading onto the same in-house packed Ni^2+^ column pre-equilibrated in TNU + 30 mM imidazole. The flowthrough containing the cleaved PINK1 precursor was collected and dialysed overnight in TU buffer (50 mM Tris, 6 M urea, pH 8.0) at 4 °C. The following day, the dialysed sample was loaded onto a 5 mL HiTrap SP HP column (GE Healthcare) equilibrated in TN(50)U (50 mM Tris, 50 mM NaCl, 6 M urea, pH 8.0). The column was washed in TN(50)U buffer prior to elution over a gradient of 0–500 mM NaCl in TNU buffer. Pooled elution fractions were concentrated in a 30 K MWCO centrifugal filter (Millipore) at 4000×*g* before being aliquoted, snap frozen in liquid nitrogen and stored at −80 °C.

### Purification of ACP1-pep86

BL21 (DE3) cells expressing the ACP1-pep86 precursor were cultured in LB media overnight prior to being sub-cultured into 2xYT media. On reaching OD_600_ = 0.6 cells were induced by the addition of IPTG. Cells were harvested 2–3 h post-expression and lysed via a cell disruptor (Constant Systems Ltd.). Inclusion bodies containing ACP1-pep86 were progressively solubilised into 1xTK buffer (20 mM Tris pH 8.0, 50 mM KCl, pH 8.0) + 6 M urea and then loaded onto a 5 mL HisTrap FF column (Cytiva). The column was washed in 1xTK + 6 M urea + 50 mM imidazole, and protein was eluted at 300 mM imidazole. Pooled elution fractions were loaded onto a 5 mL HiTrap Q HP anion exchange column (Cytiva), and a salt gradient up to 1 M KCl was applied to elute the bound protein. Desalting was carried out by centrifugation in a 10,000 MWCO spin concentrator and subsequent dilution in 1× TK buffer + 6 M urea. An extinction coefficient of 14,440 M^−1^ cm^−1^ was used to determine protein concentration.

### Purification of *MitoLuc* components

GST-tagged recombinant perfringolysin (rPFO), GST-Dark peptide and His-tagged Su9-EGFP-pep86 were purified as in (Needs et al, 2023).

### Permeabilised cell *MitoLuc* import assay

HEK cells were seeded at 600,000 cells/well in six-well plates and transfected with pXLG3-eqPF670-P2A-Cox8a-11S (matrix 11S) and incubated at 37 °C for 48 h. Alternatively, cells were transfected with pXLG3-eqPF670-P2A-Smac/DIABLO(1-57)-11S (IMS 11S) and incubated at 37 °C for 24 h. Cells were subsequently trypsinised, diluted in normal glucose growth medium and seeded on 96-well plates at a density of 20,000 cells/well. Cells were cultured overnight at 37 °C prior to commencement of the *MitoLuc* import assay. Cells were washed 2× with 1× mannitol respiration buffer pH 7.3 (225 mM mannitol, 10 mM HEPES, 2.5 mM MgCl_2_, 40 mM KCl, 2.5 mM KH_2_PO_4_, 0.5 mM EGTA) and then 100 uL *MitoLuc* import buffer pH 7.3 (1× MR Buffer, 10 µM GST-Dark, 0.1% Prionex, 0.1 mg/mL creatine kinase, 5 mM phosphocreatine, 1 mM ATP, 3 nM rPFO, 1:400 Nano-Glo luciferase assay substrate (Promega), 5 mM succinate, 1 µM rotenone) added to each well. A baseline luminescence was read before proteins were injected at 0.9 µM final concentration. Subsequent luminescence was monitored on a CLARIOstar Plus plate reader (BMG LabTech). IMS *MitoLuc* assay data were fit to the same two-step model as in (Ford et al, 2022).

Note that results contained within this paper give further confidence (beyond (Needs et al, 2023, 2024)) of the intended location of targeted 11S. The apparent failure of the C-terminus of PINK1-3EA (DDL) to enter the matrix (in contrast to the IMS) demonstrates that the matrix targeting of 11S is effective; if there was significant residual 11S in the IMS, then this result (Fig. 7C) would not have been negative. Likewise, the luminescent signal liberated from PINK1-3EA (DDL) demonstrates that the 11S is IMS-localised. If it were matrix-localised, then there would have been no signal, as we know this variant fails to get there.

Also note that we have no evidence from multiple studies that the incorporation of the pep86 fragment internally or at the C-terminus of the import protein has an impact on transport activity (Pereira et al, 2019; Needs et al, 2024, 2023; Ford et al, 2022; Ford and Collinson, 2024). Therefore, given that pep86 was strategically located in non-essential regions of PINK1, we are very confident that the inclusion of these small stretches of sequence (11 amino acids) will have no impact on its import activity. Though we cannot rule out that they would affect PINK1’s ability to accumulate at the outer membrane (which in any case was not being analysed here).

### Western blotting

Protein samples to be analysed by western blotting were prepared first for SDS-PAGE by boiling gel samples at 95 °C in 1× LDS + 25 mM DTT prior to running on a 4–12% BOLT gel (Thermo Fisher Scientific), run at 200 V for 25 min. Gels were transferred onto 0.45-μm nitrocellulose blotting membrane (Cytiva) in 1× transfer buffer (0.34 M Tris, 0.26 M glycine, 0.14 M tricine, 2.5 mM EDTA) via the semi-dry Pierce Power Station transfer system (Thermofisher Scientific) at 25 V, 2.5mAmp for 10 min. Nitrocellulose membranes were subsequently blocked in 1× TBS-T + 5% (w/v) milk for 1 h at room temperature before being probed with primary antibody (Beta actin— Sigma-Aldrich A2228 1:10,000, PINK1—cell signalling technology 6946 1:500, PINK1— Novus Biologicals BC100-494, 1:1000, PARL—Abcam ab118554, 1:1000) in 1× TBS-T + 5% (w/v) milk overnight at 4 °C. Membranes were washed in 1× TBS-T + 5% milk for 30 min and subsequently probed with secondary antibody in TBS-T + 5% milk. Membranes were once again washed for 30 min in 1× TBS-T + 5% milk, incubated with SuperSignal^TM^ West Femto reagent and imaged on an Odyssey Fc (LI-COR).

### Protein model building

Protein models were built using AlphaFold2, running AlphaFold Multimer v3 via ColabFold v1.5.2 (Jumper et al, 2021; Evans et al, 2022; Mirdita et al, 2022). Models were built as per Table 1. The full PINK1/PARL/Tim17/Tim23/Tim44 complex was constructed by overlaying PARL from the individual models. No structural clashes were observed, suggesting that this complex is both physically and physiologically plausible. Five rounds were run for each prediction, with the best-scoring model used for later analysis. Images were made with PyMOL or VMD. All AlphaFold2 models are available for download at https://osf.io/xj9ca/ along with PAE and plDDT plots.

### Molecular dynamics simulations

The combined AlphaFold2 PINK1/PARL/Tim17/Tim23/Tim44 complex was used to seed MD simulations (with Tim44 trimmed as described in Table 1). The PINK1 loop between residues 180 and 209 was also removed due to low confidence and replaced with a GSGSGSG linker. Alternatively, simulations were built using PARL/Tim17/Tim23PINK1 with PINK1 trimmed to the TMD (residues 90-130) or the PINK1_TMD/Tim17/Tim23/ROMO1 complex with the GSGSGSGS linker removed and PINK1 trimmed to the TMD (residues 90-130). Models were built into simulation systems using CHARMM-GUI (Jo et al, 2007; Lee et al, 2016). Protein atoms were described with the CHARMM36m force field (Best et al, 2012; Huang et al, 2017). Side chain pKas were assessed using propKa3.1 (Søndergaard et al, 2011), and all side chain side charge states were set to their default. The proteins were built into membranes comprising 4.5:4.5:1 POPE, POPC, and cardiolipin (tetraoleyl tails). The membranes were solvated with TIP3P waters and neutralised with K^+^ and Cl^-^ to 150 mM.

Each system was minimised and equilibrated according to the standard CHARMM-GUI protocol, with a 2 ns final equilibration step. Production simulations were run in the NPT ensemble, with temperatures held at 303.5 K using a velocity-rescale thermostat and a coupling constant of 1 ps, and pressure maintained at 1 bar using a semi-isotropic Parrinello-Rahman pressure coupling with a coupling constant of 5 ps (Bussi et al, 2007; Parrinello and Rahman, 1981). Short-range van der Waals and electrostatics were cut off at 1.2 nm. Simulations were run to 450 ns with 5 repeats for the full complex, or to 300 or 500 ns for the PARL and ROMO1 TMD systems, respectively, with three repeats.

All simulations were run in Gromacs 2020.1 (Abraham et al, 2015). Data were analysed using Gromacs tools and VMD (Humphrey et al, 1996). Lipid interactions were analysed using the PyLipID package (Song et al, 2022) using cutoffs of 0.35 and 0.5 nm. Plots were made using Prism 10.

### Statistics

The in-built statistical tests on GraphPad Prism 7 were used for statistical analysis. A combination of unpaired and paired *t* tests as well as one-way ANOVA with Tukey’s post hoc multiple comparisons was used to determine if a statistical significance was observed. The *P* value cut-off for significance was <0.05, with significance values graded as follows: n.s. *P* = >0.05, *P = $\le$0.05, **P = $\le$0.01, ***P = $\le$0.001 and ****P = $\le$0.0001.

## Supplementary information

Peer Review File

Appendix

Source data Fig. 2

Source data Fig. 3

Source data Fig. 4

Source data Fig. 7

Source data Fig. 8

## Data Availability

All data are available in the main text or supplementary material. AlphaFold2 models are available for download at https://osf.io/xj9ca/ along with PAE and pLDDT plots. The source data of this paper are collected in the following database record: biostudies:S-SCDT-10_1038-S44318-026-00789-x.
