# Supplementary material for: Transmembrane domain switching controls PINK1 import and fate in mitochondria
Source: EMBO J. 2026 May 26;45(13):4447–68. doi: 10.1038/s44318-026-00789-x (PMC13324151; doi:10.1038/s44318-026-00789-x)
Supplement: Supplementary file 2 — Appendix [file 44318_2026_789_MOESM2_ESM.pdf]

# Appendix for Transmembrane domain Switching Controls PINK1 Import and Fate in Mitochondria

James S. Lorriman[1,*], Rhiannon J. Hughes[1,*], Adam G. Grieve[1,†], Robin A. Corey[2,†], Ian Collinson[1,†]

[1], *School of Biochemistry, University of Bristol, Bristol, BS8 1TD, UK*

[2], *School of Physiology, Pharmacology and Neuroscience, University of Bristol, BS8 1TD, UK*

[*], these authors contributed equally to the study

[†], corresponding authors: ian.collinson@bristol.ac.uk, adam.grieve@bristol.ac.uk, and robin.corey@bristol.ac.uk

**Appendix Table of Contents**

| | |
|---|---|
| Appendix Title page | 1 |
| Appendix Table S1 | 2 |
| Appendix Figure S1 | 3 |
| Appendix Figure S2 | 4 |
| Appendix Figure S3 | 5 |
| Appendix Figure S4 | 6 |
| Appendix Figure S5 | 7 |
| Appendix Figure S6 | 8 |
| Appendix Figure S7 | 9 |
| Appendix Figure S8 | 10 |
| Appendix Figure S9 | 11 |
| Appendix Figure S10 | 12 |
| Appendix Figure S11 | 13 |
| Appendix Figure S12 | 14 |
| Appendix Figure S13 | 15 |
| Appendix Figure S14 | 16 |
| Appendix Figure S15 | 17 |
| Appendix Figure S16 | 18 |
| Appendix References | 19 |

## APPENDIX TABLE S1:

### 1.1 Luminescent PINK1 constructs

| Construct | Amino acid position of luminescent pep86 reporter | Domain position of luminescent pep86 reporter |
|---|---|---|
| **PINK1 LDD** | 53 | N-terminal, between MTS and OMS. |
| **PINK1 DLD** | 140 | Between the TMD and kinase domain. |
| **PINK1 DDL** | 582 | C-terminal, following the kinase domain. |

### 1.2 Mutant PINK1 variants

| Variant | Mutation | Description |
|---|---|---|
| **PINK1 (GLGLGL)** | N/A | Wild-type human PINK1. |
| **PINK1 ∆1-50** | Deletion of residues 1-50 | Deletion of the classical MTS. |
| **PINK1 ∆1-91** | Deletion of residues 1-91 | Deletion of the classical MTS and OMS. |
| **PINK1 ∆1-150** | Deletion of residues 1-150 | Deletion of the classical MTS, OMS and TMD. Retains only the kinase domain. |
| **PINK1 3EA** | E112, E113, E117 to A112, A113, A117 | Mutation of three conserved glutamate residues to alanine immediately downstream of the TMD is postulated to destabilise PINK1 interactions with PARL/ROMO1-containing complexes. |
| **PINK1 I111S** | I111 to S | Mutation of isoleucine-111 to serine is a PD-associated mutation (Marongiu *et al*, 2007) , I111 thought to be important in stabilising PINK1 interactions. |
| **PINK1 (∆TMD)** | Deletion of residues 94-110 | Deletion of the PINK1 TMD to remove the PARL cleavage site (A103/F104), thus abrogating cleavage *(Deas et al, 2011).* |
| **PINK1 (ALALAL)** | G105, G107, G109 to A105, A107, G109 | Mutation of sequential helix-breaking glycine residues to alanine, resulting in stabilisation of the TMD in an α-helical conformation. |

## APPENDIX FIGURES AND LEGENDS:

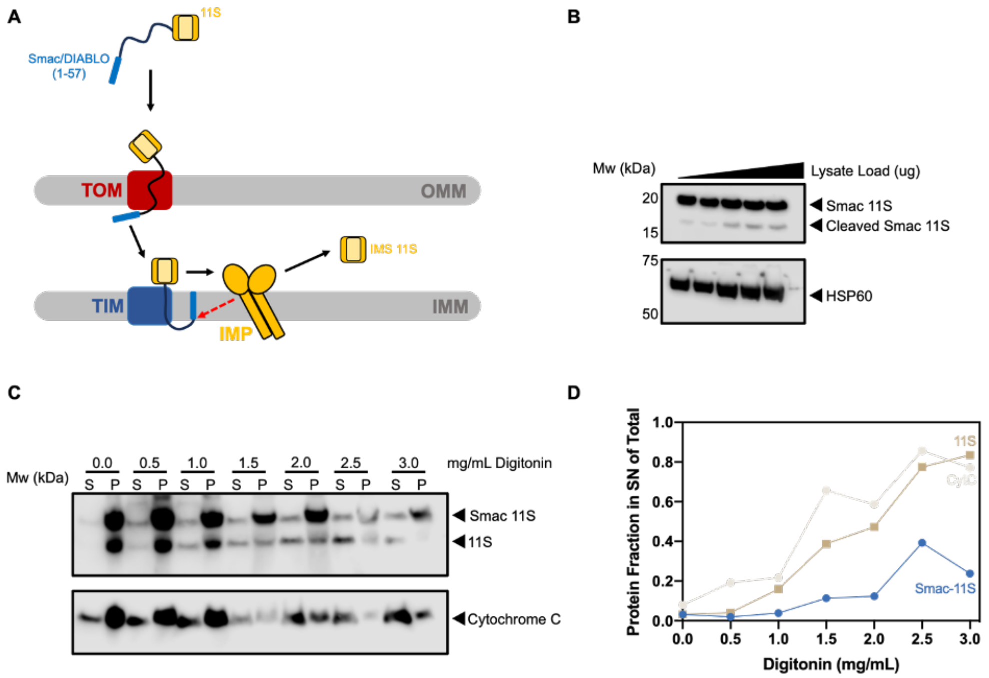


**Appendix Figure S1: The N-terminal Smac/DIABLO (1-57) fusion to 11S facilitates targeting to the IMS. (a)** Schematic representing targeting of 11S to the IMS via a fusion to the first 57 AAs from the proapoptogenic protein Smac/DIABLO. The N-terminal 57 residues facilitate targeting to the TIM complex whereby the TMD enables lateral release into the IMM by a 'stop-transfer' based mechanism. Cleavage of the N-terminal 57 residues by the IMP complex yields the 'mature' version of 11S in the IMS. **(b)** Immunoblot analysis of isolated mitochondria from HEK cells expressing the Smac/DIABLO(1-57)-11S construct. Mitochondrial lysates were loaded onto the gel in increasing increments of 20ug from 20ug-100ug left to right. Membrane was probed with anti-NanoLuc and anti-HSP60. **(c)** Immunoblot analysis of isolated mitochondria from HEK cells expressing Smac/DIABLO(1-57)-11S and exposed to increasing indicated concentrations of digitonin. 60ug mitochondrial lysate was loaded in each lane and membrane probed with anti-NanoLuc and anti-cytochrome C. S=supernatant (SN) and P=pellet (insoluble fraction). **(d)** Quantification of immunoblot as in (c). The fraction of protein present in the SN was calculated by S/(S+P).

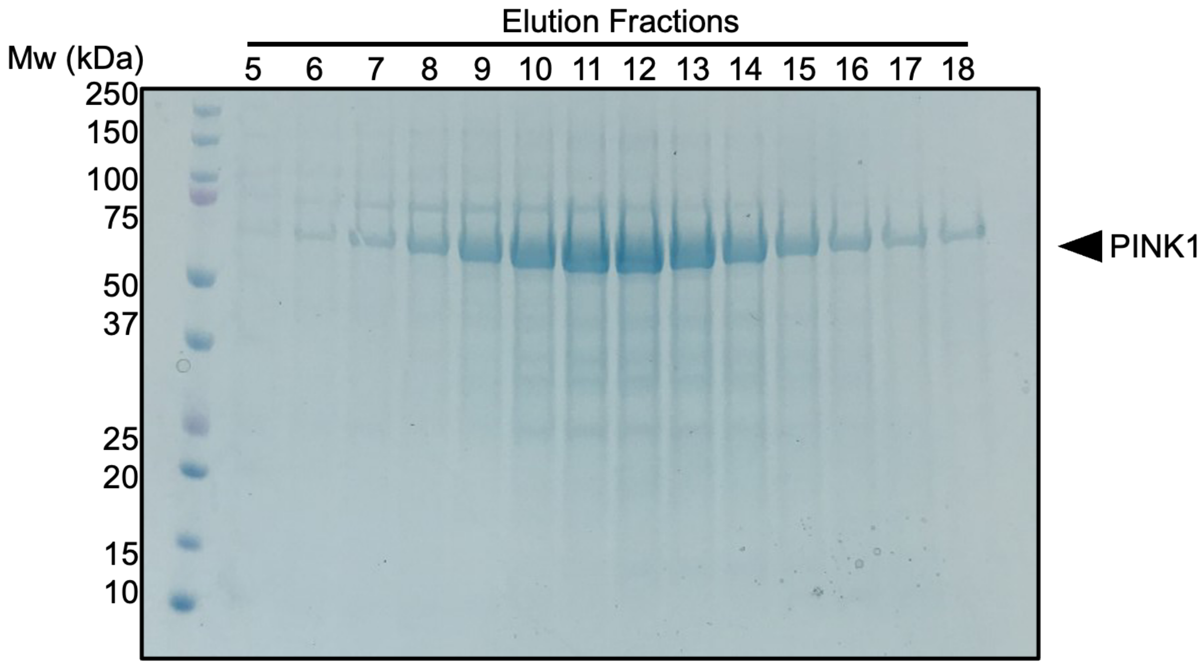


**Appendix Figure S2: Purification of human PINK1 construct.** SDS-PAGE analysis of fractions from cation exchange chromatography, stained by coomasie blue. The visualisation of PINK1 shown here is typical of the purification of all the variants generated for this study. In this case the variant DDL is presented; all the other variants, in respect of their purification, behaved in the same way.

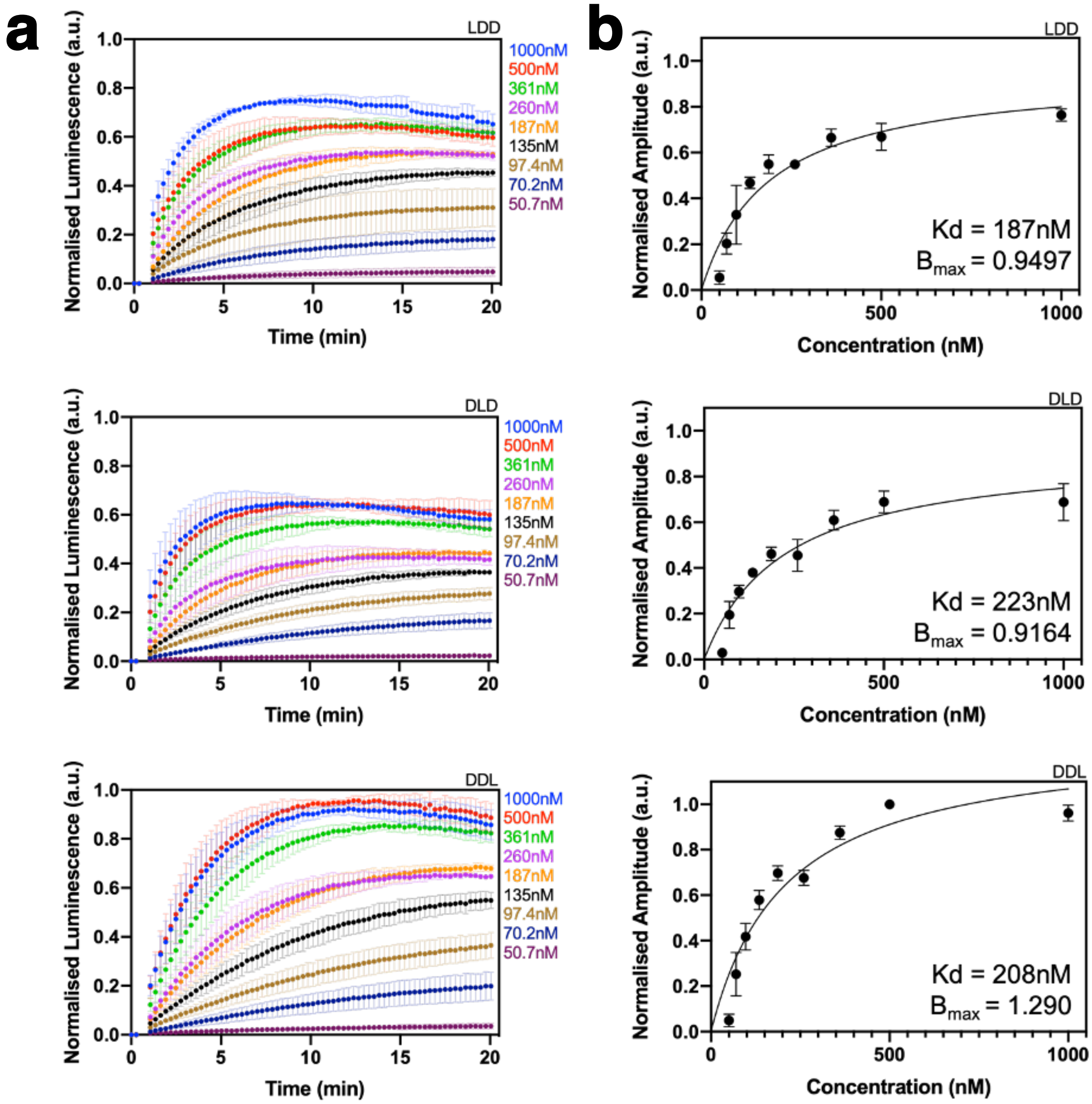


**Appendix Figure S3: Binding analysis of the PINK1 LD series variants to isolated 11S. (a)** Normalised data for PINK1 LDD, DLD and DDL binding to 11S (200pM final concentration) over an LD precursor titration range of 50.7-1000nM. Concentrations and their associated binding curves are indicated by colours. Data represent N=3, error bars for each concentration indicate SEM. **(b)** Normalised amplitude concentration dependence plots for data as in (a). For each LD precursor maximum normalised amplitude was calculated for each concentration and plotted against the corresponding concentration. Data was fit to a one-site binding model (hyperbola). Data represent N=3, error bars for each concentration indicate SEM.

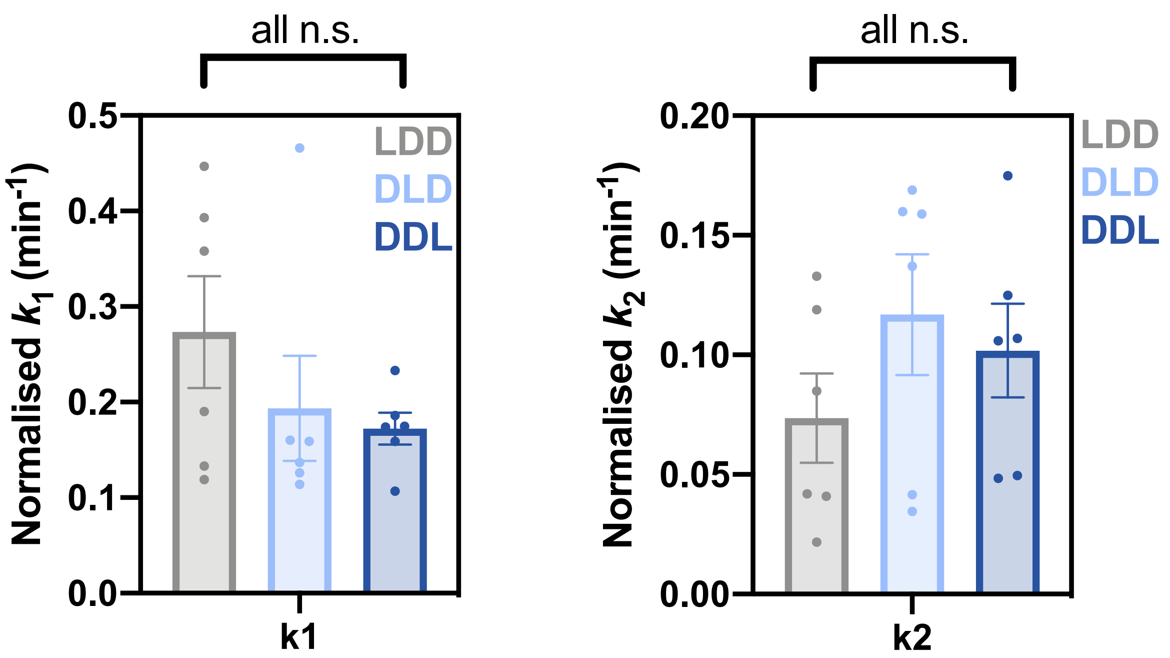


**Appendix Figure S4: Kinetic assessment of PINK1 LD series import into the IMS.** Data for each LD protein was fitted to a two-step model for import and $k_1$ **(a)** and $k_2$ **(b)** values calculated from the fitted trace. Error bars represent SEM and data are from N=6 biological repeats, where each biological repeat was calculated from three technical repeats. A paired t-test was used to determine significance.

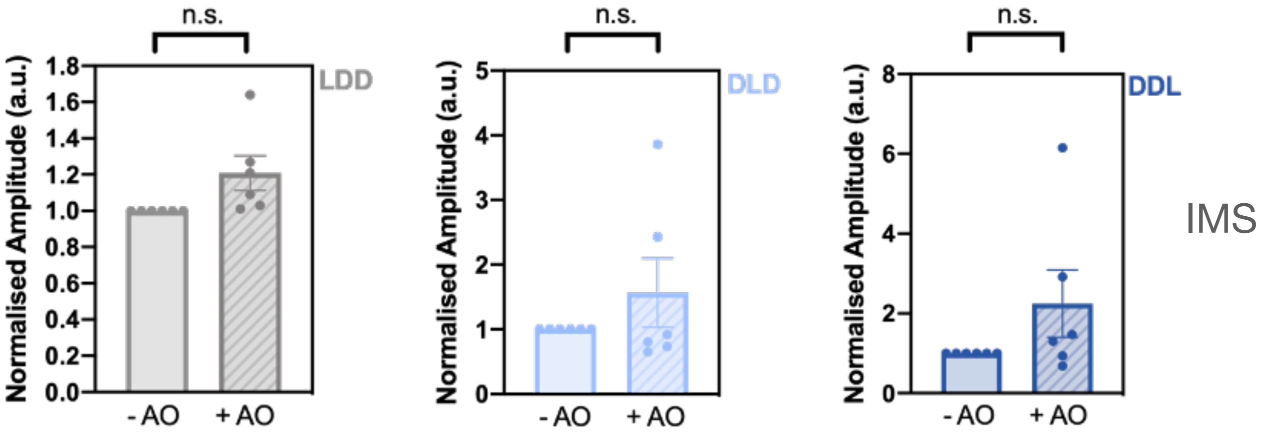


**Appendix Figure S5: Import of the PINK1 LD series into the IMS is insensitive to Antimycin A (A) and Oligomycin (O).** IMS import data was obtained in the -AO and +AO conditions for each PINK1 LD precursor and the normalised amplitude determined as before. Error bars represent SEM and N=3-6 biological replicates obtained. Amplitude significance was determined using a paired t-test, p-values = 0.0547, 0.3132 and 0.1711,

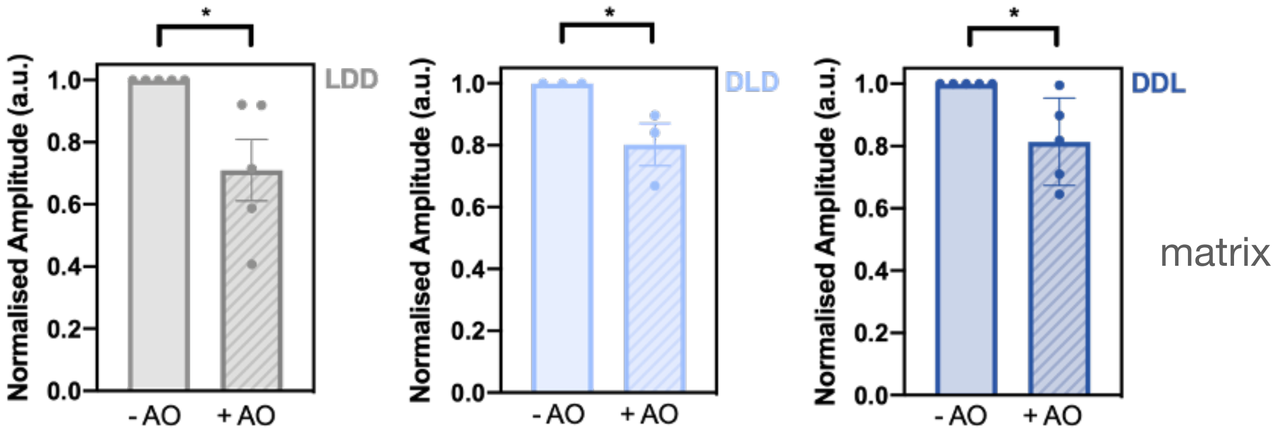


**Appendix Figure S6: Import of the PINK1 LD series into the matrix is sensitive to Antimycin A and Oligomycin.** As described for legend to Appendix Figure S5, but for matrix import data. p-values = 0.0188, 0.0442, and 0.0180.

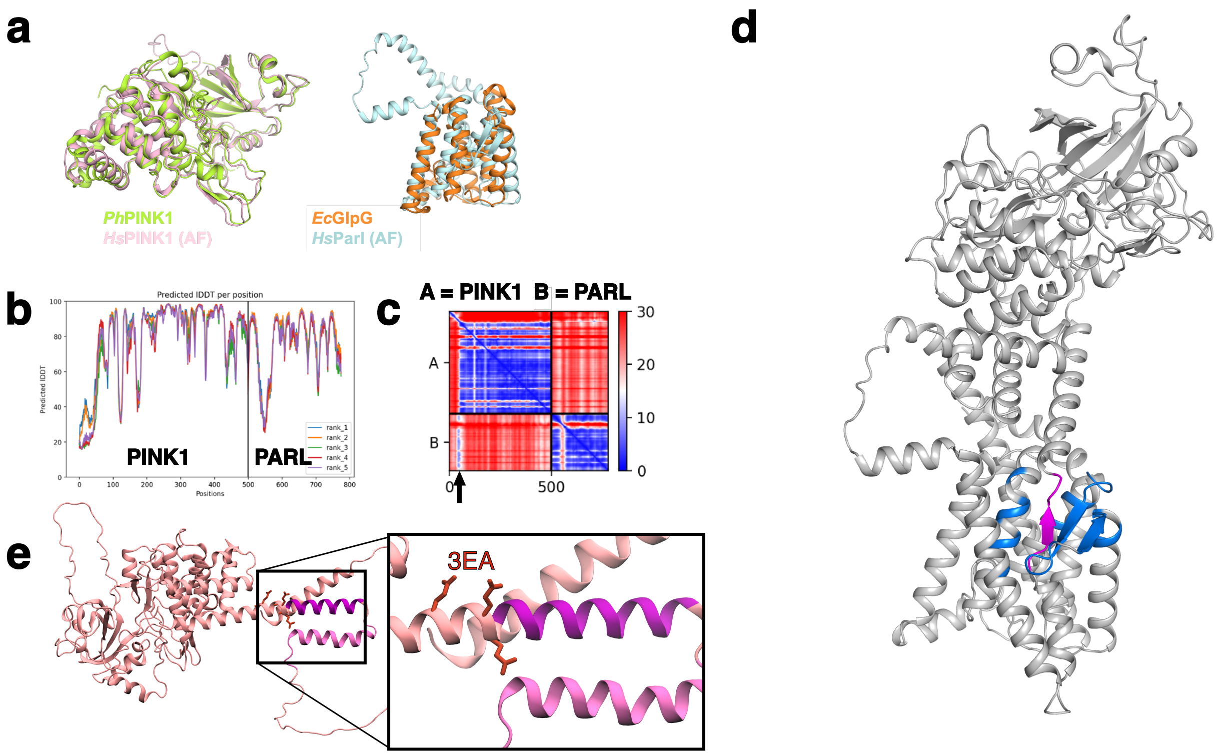


**Appendix Figure S7: AlphaFold2 modelling of the PINK1/PARL complex. (a)** Overlays of the modelled human PINK1 with a structure of a homologous PINK1 from *Pediculus humanus corporis* and the modelled PARL aligned to the *E. coli* rhomboid protease GlpG. **(b)** AlphaFold2 pLDDT for the PINK1-PARL complex model. **(c)** AlphaFold2 PAE scores for the PINK1-PARL complex. An arrow denotes the position of the PINK1-PARL β-strand which corresponds to a high PAE score. (**d**) Output from the ipSAE analysis highlighting residues (respectively pink and blue) in the PINK1-PARL AlphaFold complex within the defined PAE and distance cutoffs (<10 Å). The analysis indicates that although the overall interface is predicted with low confidence, the β-strand region of PINK1 interacting with PARL shows a comparatively higher confidence. **(e)** Human PINK1 from the EBI AlphaFold2 database. The positions of the 3EA are highlighted on the inset.

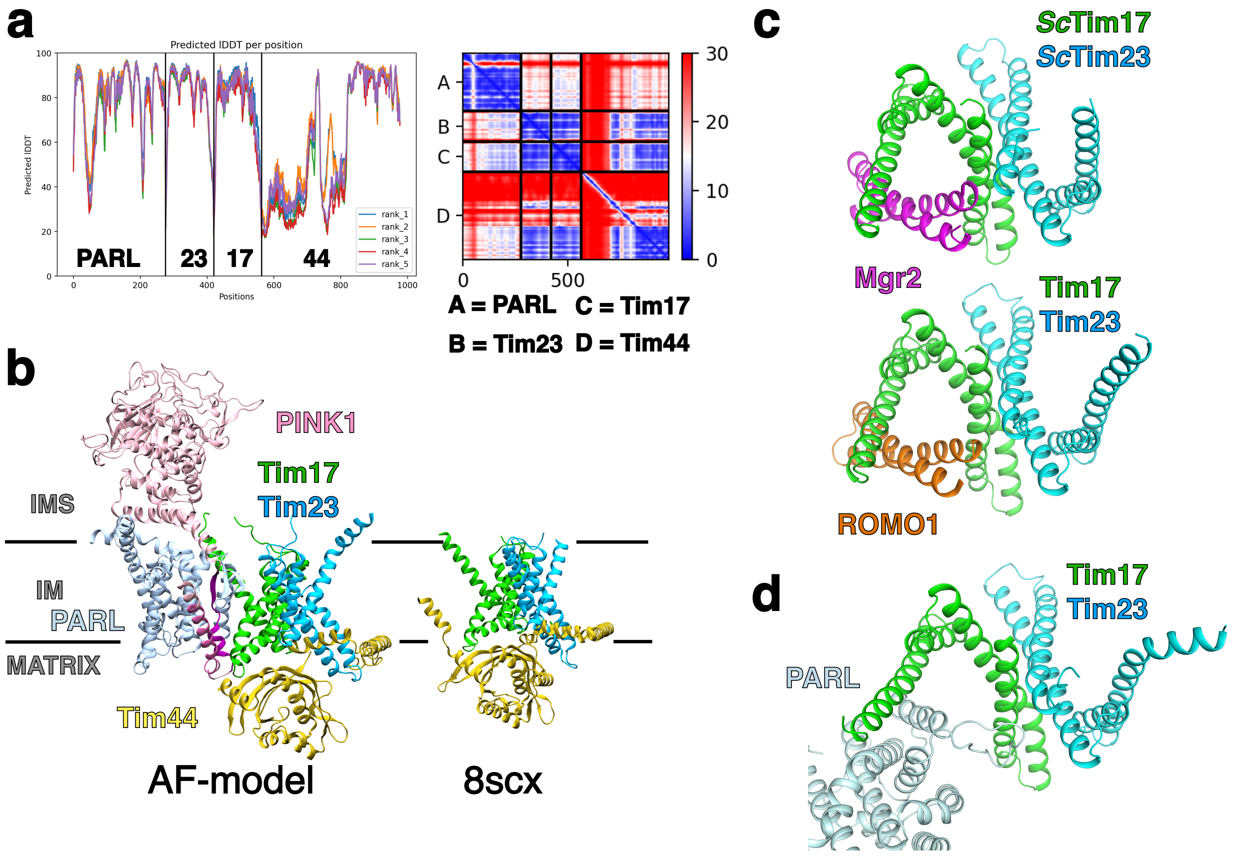


**Appendix Figure S8: AlphaFold2 modelling of the various complexes containing PINK1, PARL, Tim17, Tim23, Tim44 and Mgr2/ROMO1. (a)** AlphaFold2 pLDDT and PAE scores for the PARL-Tim17-Tim23-Tim44 complex model. **(b)** View of the hybrid AlphaFold2 PINK1-PARL-Tim17-Tim23-Tim44 model compared to the yeast Tim17-Tim23-Tim44 structure (Sim *et al*, 2023). The lines indicate the position of the bilayer. **(c)** Top view (from the IMS) of the human Tim17-23-ROMO1 (lower panel) and yeast (Sc) Tim17-Tim23-Mgr2 (upper panel) AlphaFold2 model showing the presence of the putative protein channel. **(d)** Same view as in (c) of the PARL-Tim17-Tim23 complex modelled without PINK1.

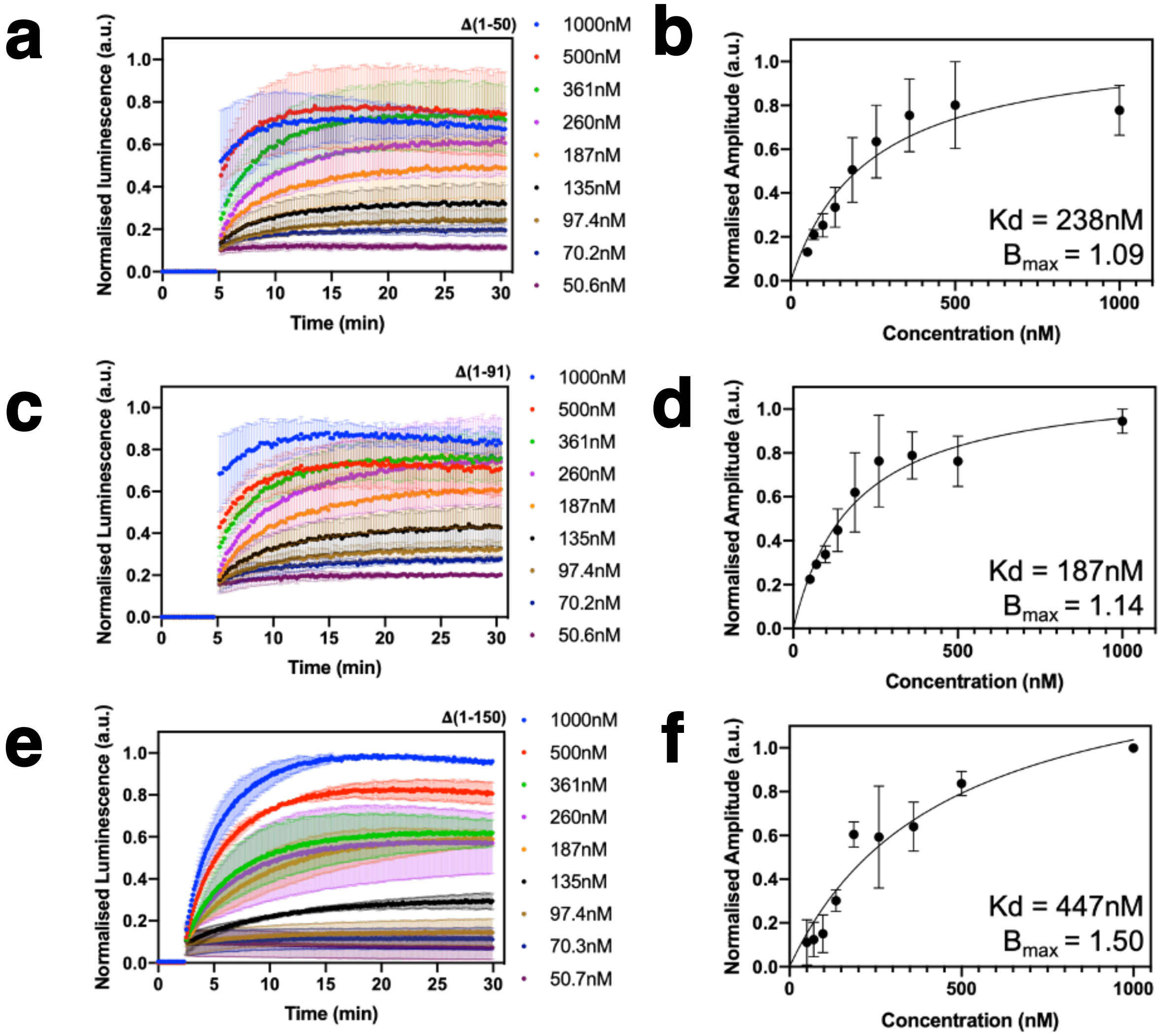


**Appendix Figure S9: Binding analysis of the truncated PINK1 precursors to isolated 11S. (a, c**, and **e)** Titration series of truncated PINK1 precursors was conducted from 50.7nM-1000nM and assayed for binding against 200pM of purified 11S. Maximum amplitudes were determined for each concentration of truncated precursor and plotted in (**b**, **d**, and **f**). Error bars represent SEM and data an N=3 biological repeats.

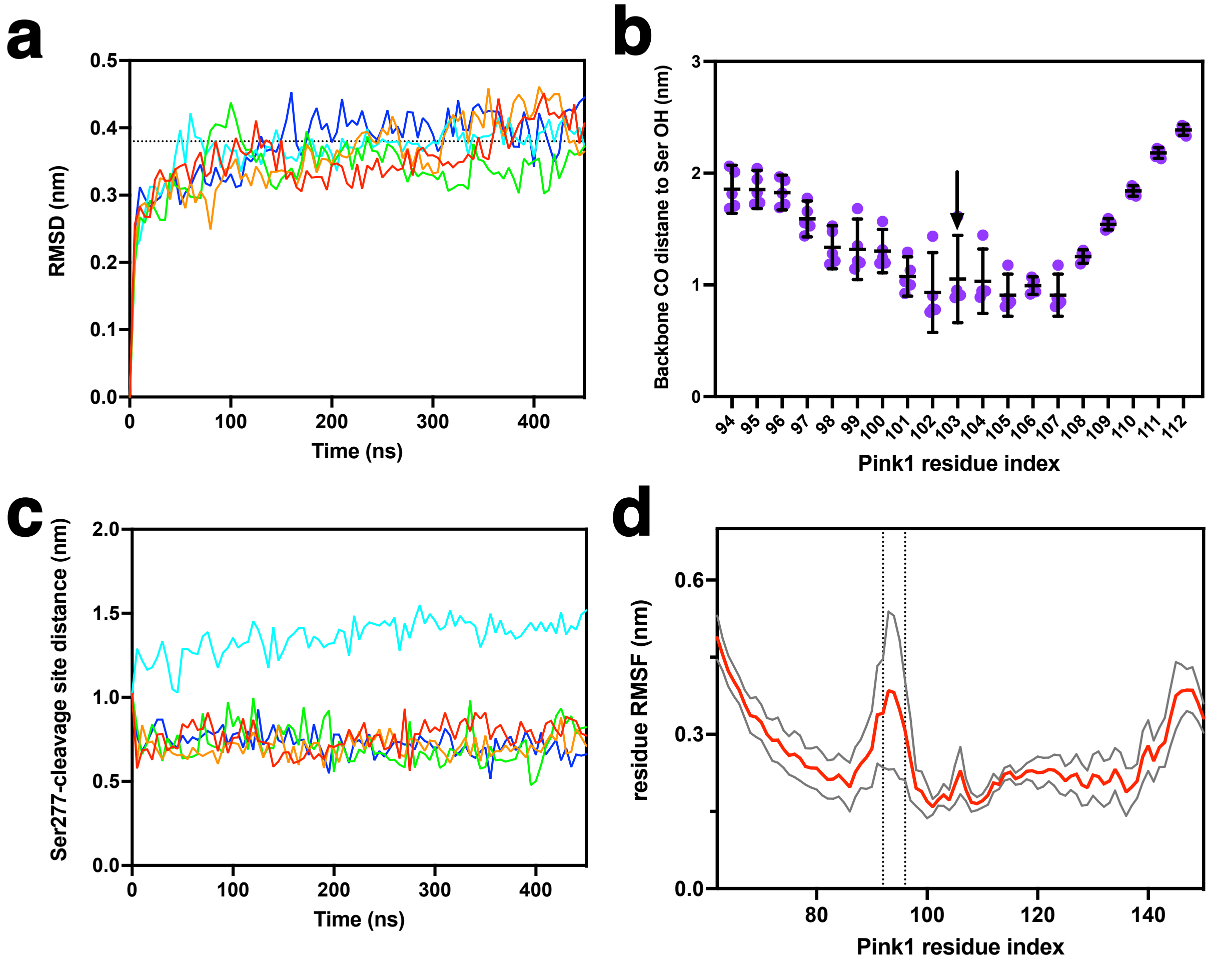


**Appendix Figure S10: Molecular dynamics supporting data. (a)** RMSDs of the simulations of the PINK1-PARL-Tim17-Tim23-Tim44 complex (excluding the PINK1 kinase domain). **(b)** Mapping distances between the Ser227 side chain hydroxyl (OH) group and the backbone carbonyl (CO) of the PINK1 TMD throughout our MD simulations reveals that the closest interacting region of PINK1 to the PARL active site is the stretch of residues from 103-104, which corresponds to the PINK1 cleavage site (arrow). **(c)** Distance plots between Ser277 and the PINK1 cleavage site over the MD simulations. **(d)** RMSF of PINK1 during the PINK1-PARL-Tim17-Tim23-Tim44 MD simulations. Dotted lines denote Cys 92 and 96.

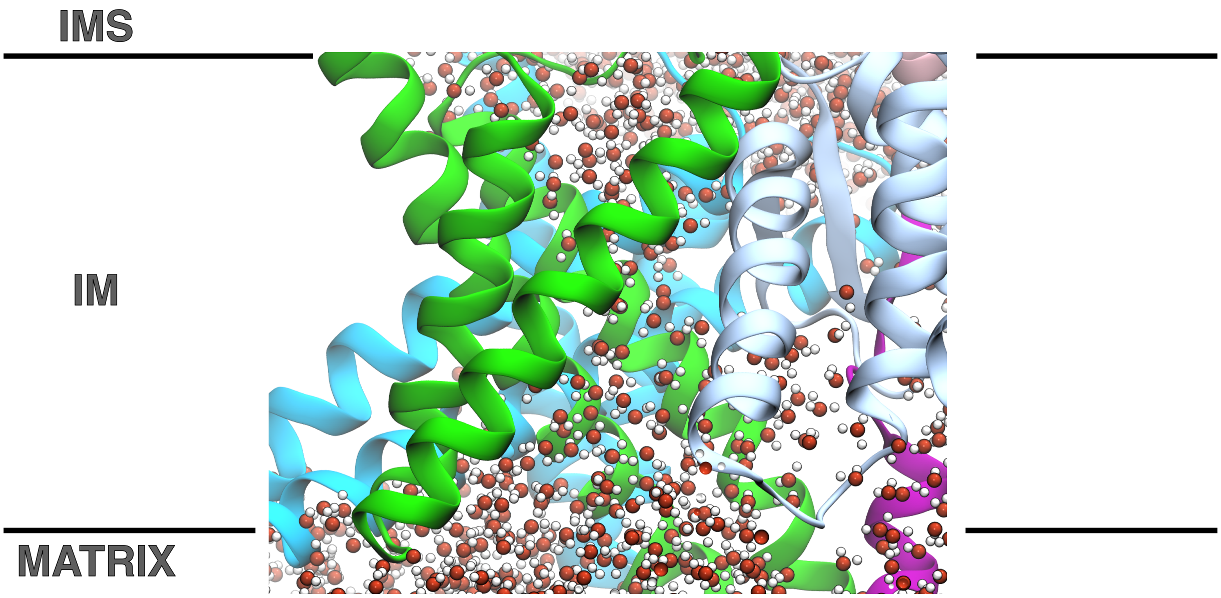


**Appendix Figure S11: A potential protein-channel at the interface between Tim17 and PARL.** Views of an MD simulation from the side of the membrane, showing Tim17 (green), Tim23 (blue), PARL (cyan) and PINK1 (pink). The location of the water molecules indicates an enclosed solvated channel running through the centre of the complex. The lines indicate the position of the bilayer.

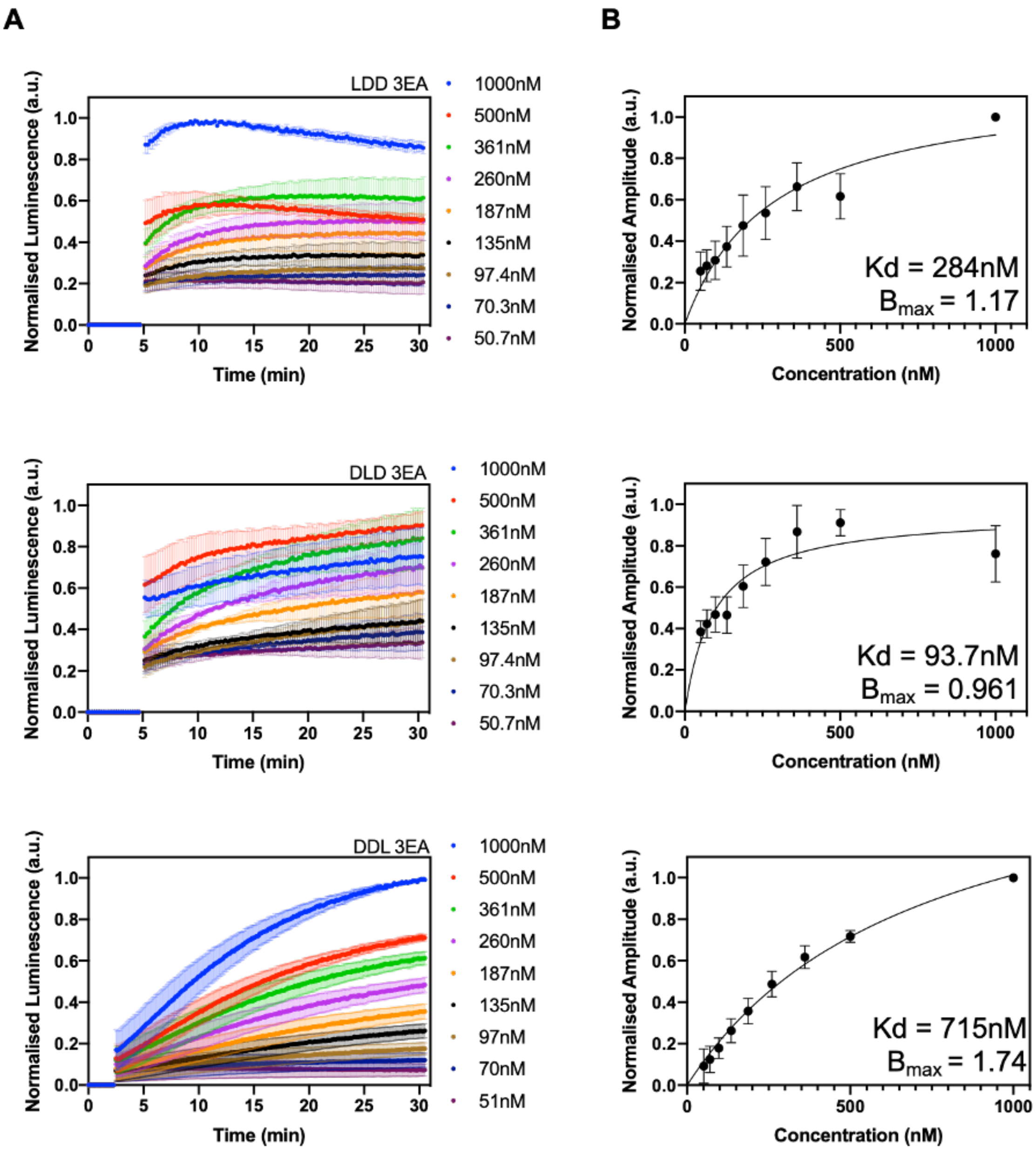


**Appendix Figure S12: Binding analysis of the PINK1 3EA variants to 11S.** (**a**) A titration series of the PINK1 LD 3EA precursors was conducted from 51nM to 1000nM and assayed for binding against a fixed concentration of purified 11S, 200pM. (**b**) Normalised maximum amplitudes were calculated and plotted against each concentration of precursor. Data were fit to a simple model for one site binding and the associated $K_d$ and $B_{max}$ (maximum amplitude) values were calculated based on the fit. Data represent an N=3 biological repeats and error bars the SEM.

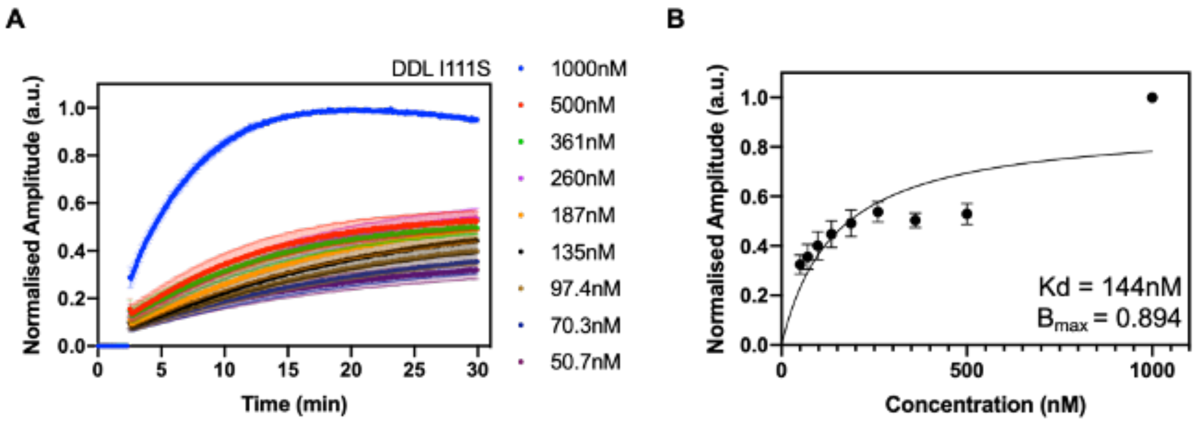


**Appendix Figure S13: Binding Analysis of the pathogenic PINK1 Variant I111S to 11S. (a)** A titration series of DDL I111S precursor was conducted from 50.7nM to 1000nM and assayed for binding against 200pM purified 11S. **(b)** Maximum amplitude at each concentration of I111S was calculated and plotted. Data was fitted to a one-site binding model and associated $K_d$ and $B_{max}$ (maximum amplitude) values shown.

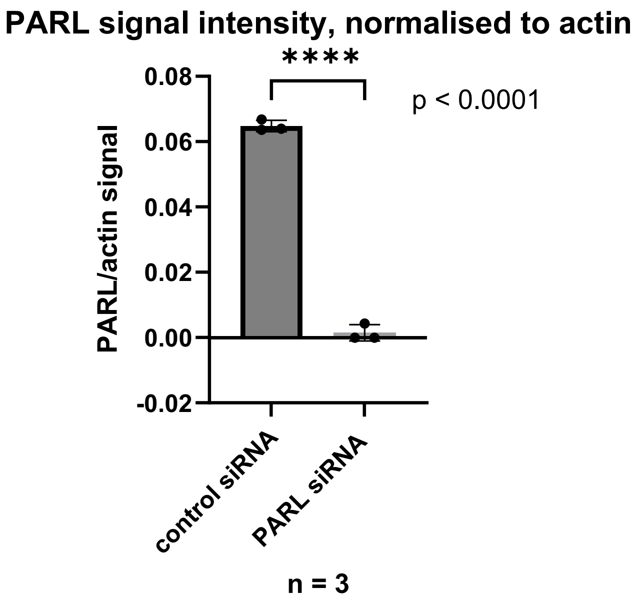


**Appendix Figure S14: Quantification of PARL knockdown by siRNA** (used in Figure 8 and Appendix Figure S15).

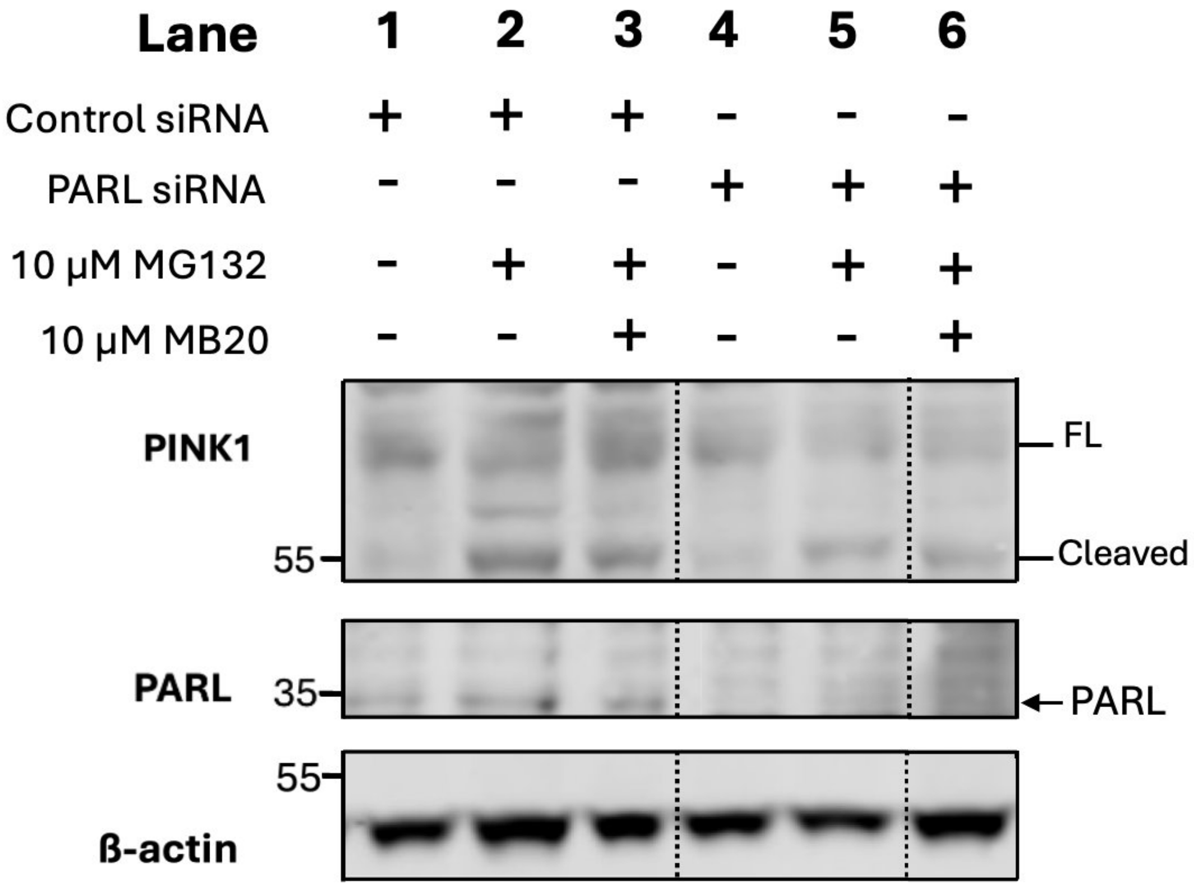


**Appendix Figure S15: PARL knockdown abrogates MB20 mediated increase in PINK1 cleavage.** Western blot of full-length or cleaved PINK1 with cell lysates from control or PARL siRNA-treated cells with addition of MG132 and MB20. Novus Biologics PINK1 antibody used.

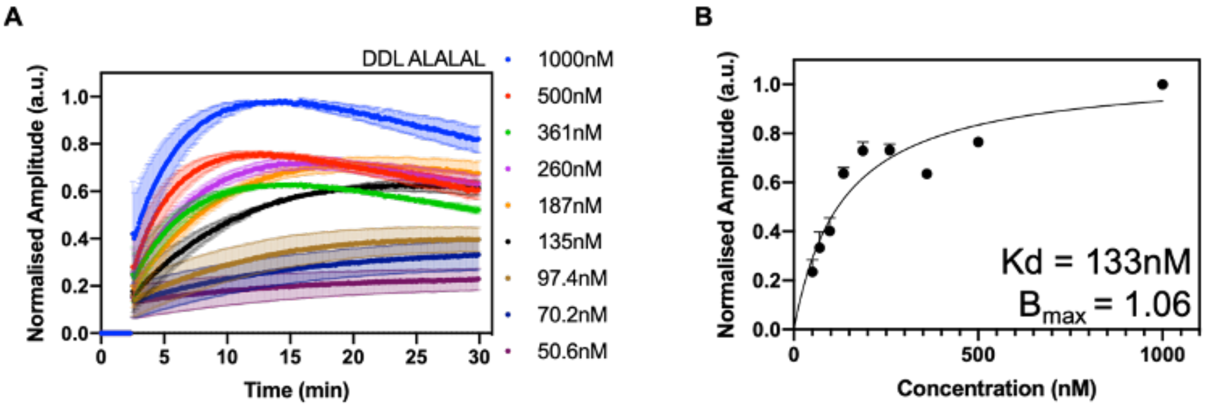


**Appendix Figure S16: Binding analysis of the PINK1 ALALAL variant to 11S. (a)** A titration series of PINK1 ALALAL was conducted from 50.6nM to 1000nM and assayed for binding against 200pM purified 11S. **(b)** Maximal amplitude at each ALALAL concentration was plotted and data fit to a simple one site binding model, resulting $K_d$ and $B_{max}$ (maximum amplitude) data are shown.

## APPENDIX REFERENCES:

Deas E, Plun-Favreau H, Gandhi S, Desmond H, Kjaer S, Loh SHY, Renton AEM, Harvey RJ, Whitworth AJ, Martins LM, *et al* (2011) PINK1 cleavage at position A103 by the mitochondrial protease PARL. *Hum Mol Genet* 20: 867–879

Marongiu R, Brancati F, Antonini A, Ialongo T, Ceccarini C, Scarciolla O, Capalbo A, Benti R, Pezzoli G, Dallapiccola B, *et al* (2007) Whole gene deletion and splicing mutations expand the PINK1 genotypic spectrum. *Hum Mutat* 28: 98–98

Sim SI, Chen Y, Lynch DL, Gumbart JC & Park E (2023) Structural basis of mitochondrial protein import by the TIM23 complex. *Nature* 621: 620–626
