# Supplementary figures and images for: Transmembrane domain switching controls PINK1 import and fate in mitochondria
Source: EMBO J. 2026 May 26;45(13):4447–68. doi: 10.1038/s44318-026-00789-x (PMC13324151; doi:10.1038/s44318-026-00789-x)

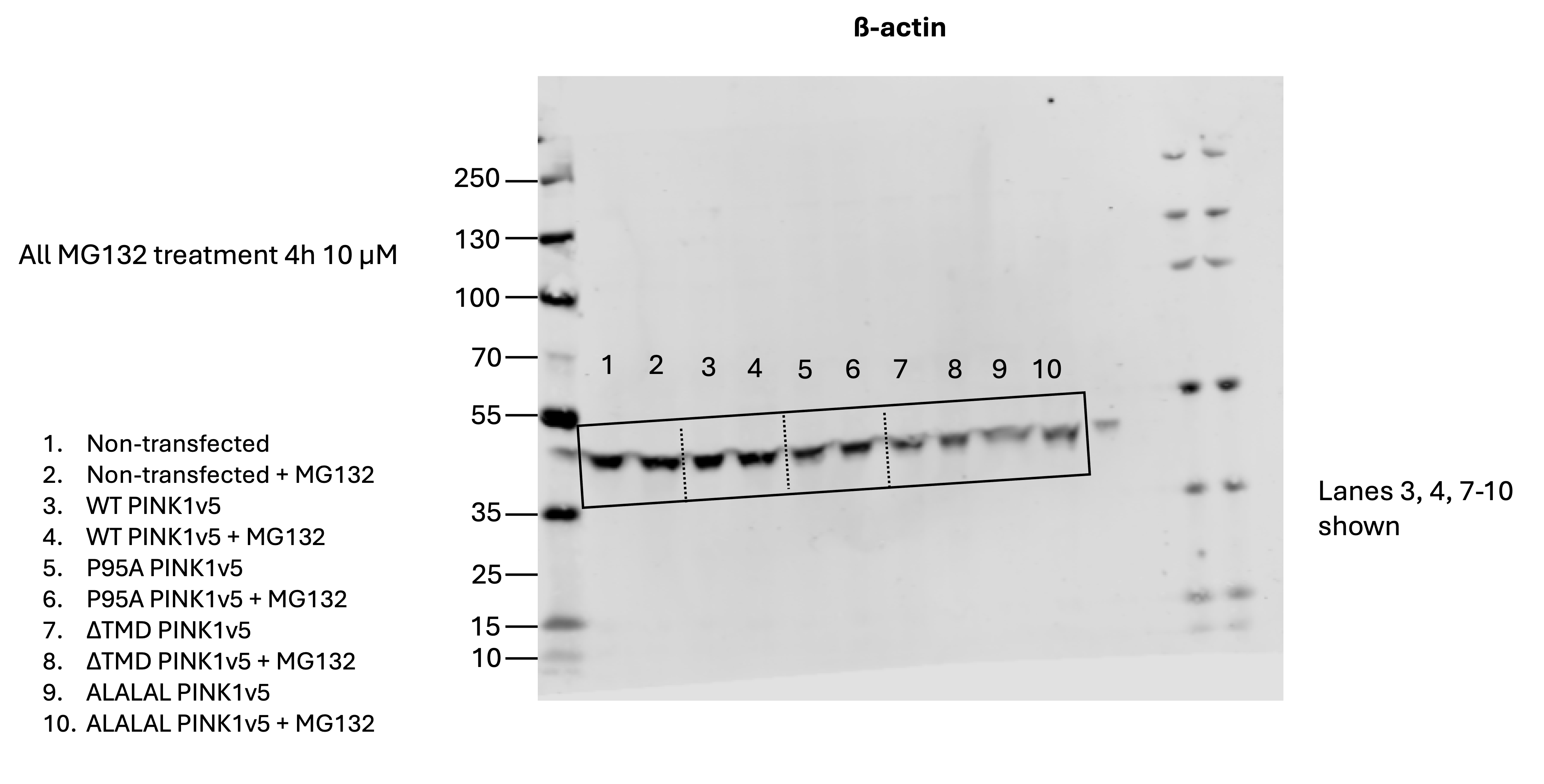

Supplement: Supplementary file 7 — Source data Fig. 8 [file 44318_2026_789_MOESM7_ESM.zip › Fig8/8b_blot_2.png]

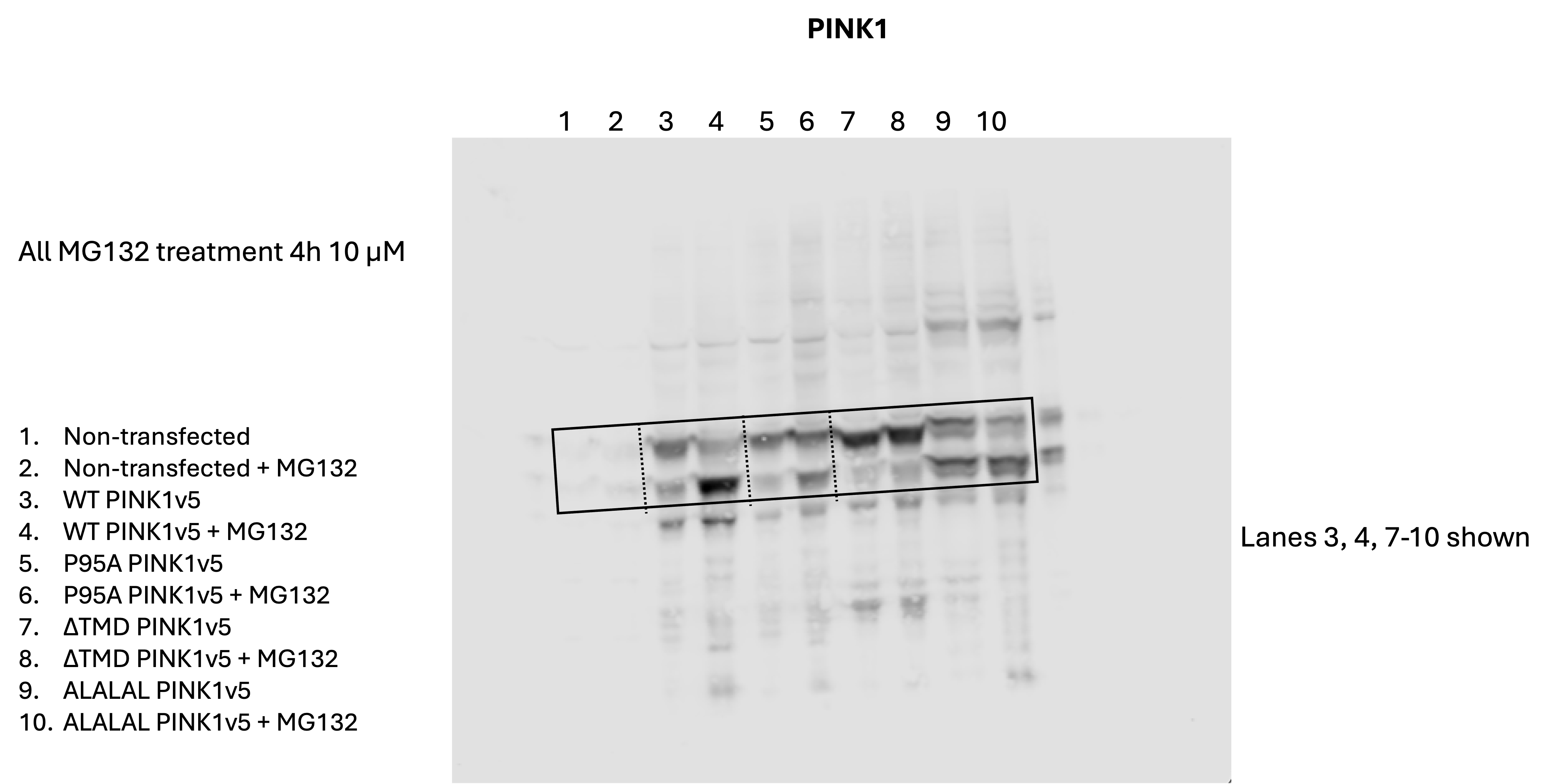

Supplement: Supplementary file 7 — Source data Fig. 8 [file 44318_2026_789_MOESM7_ESM.zip › Fig8/8b_blot_1.png]

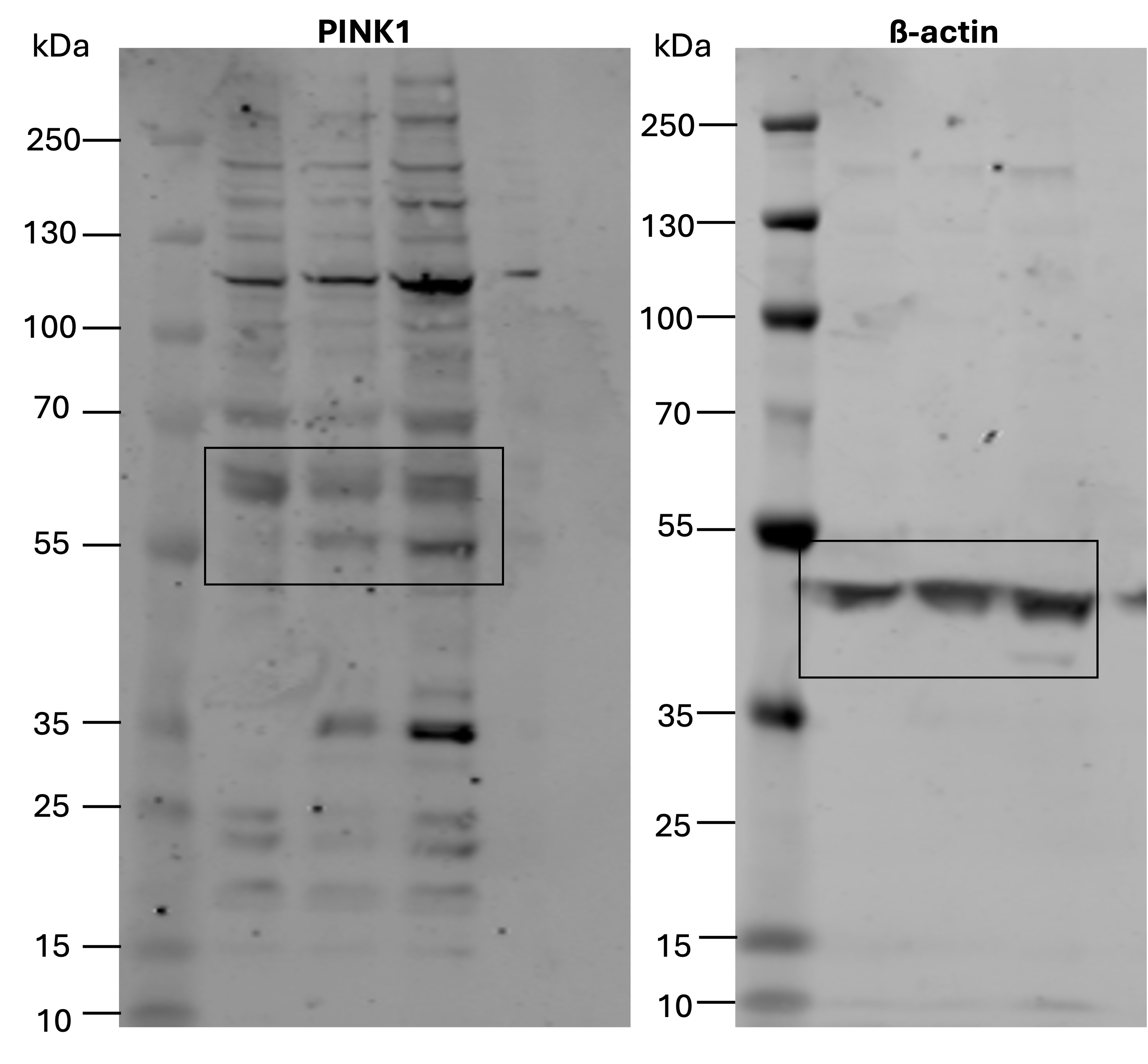

Supplement: Supplementary file 7 — Source data Fig. 8 [file 44318_2026_789_MOESM7_ESM.zip › Fig8/8a_blot.png]

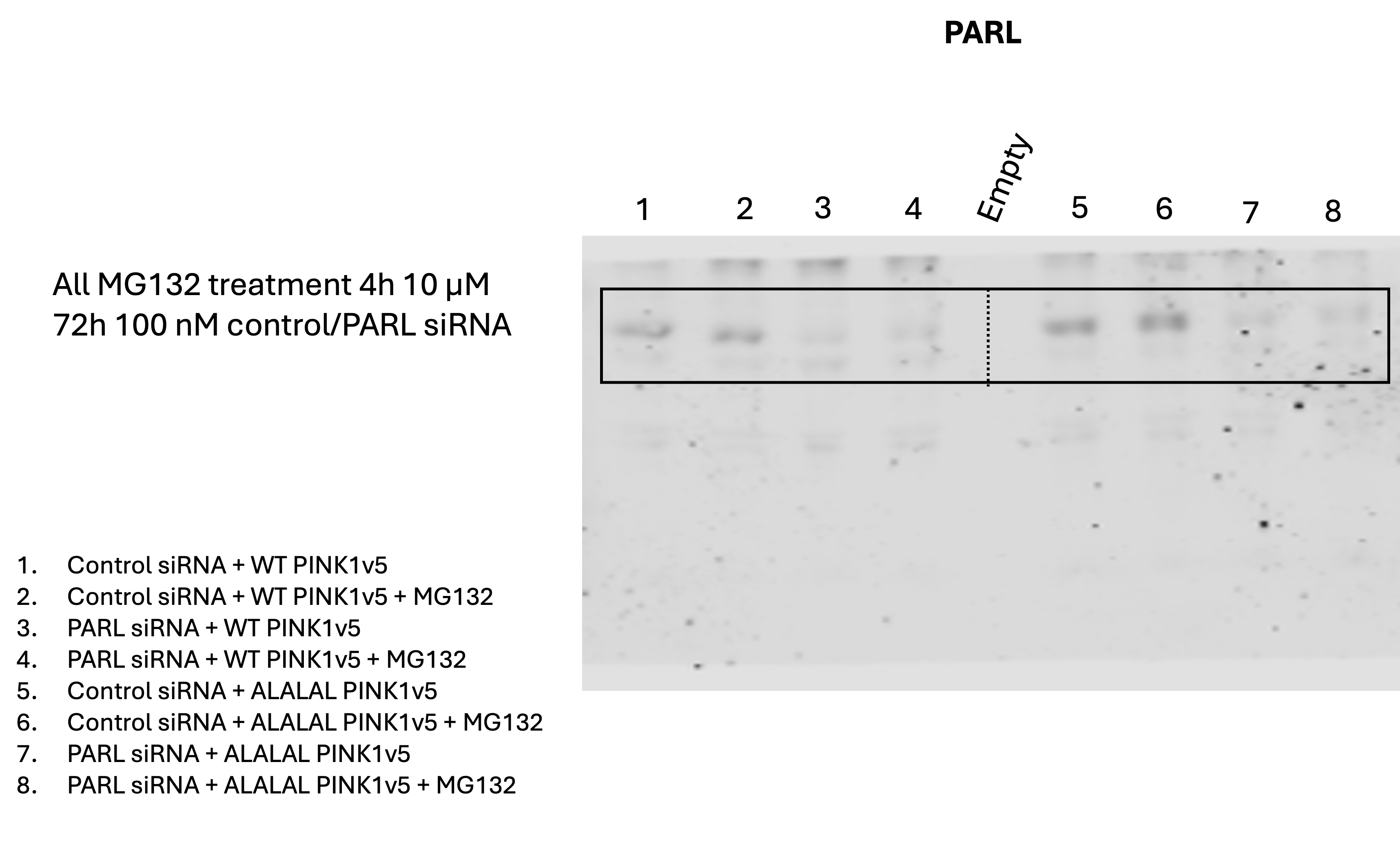

Supplement: Supplementary file 7 — Source data Fig. 8 [file 44318_2026_789_MOESM7_ESM.zip › Fig8/8c_blot_2.png]

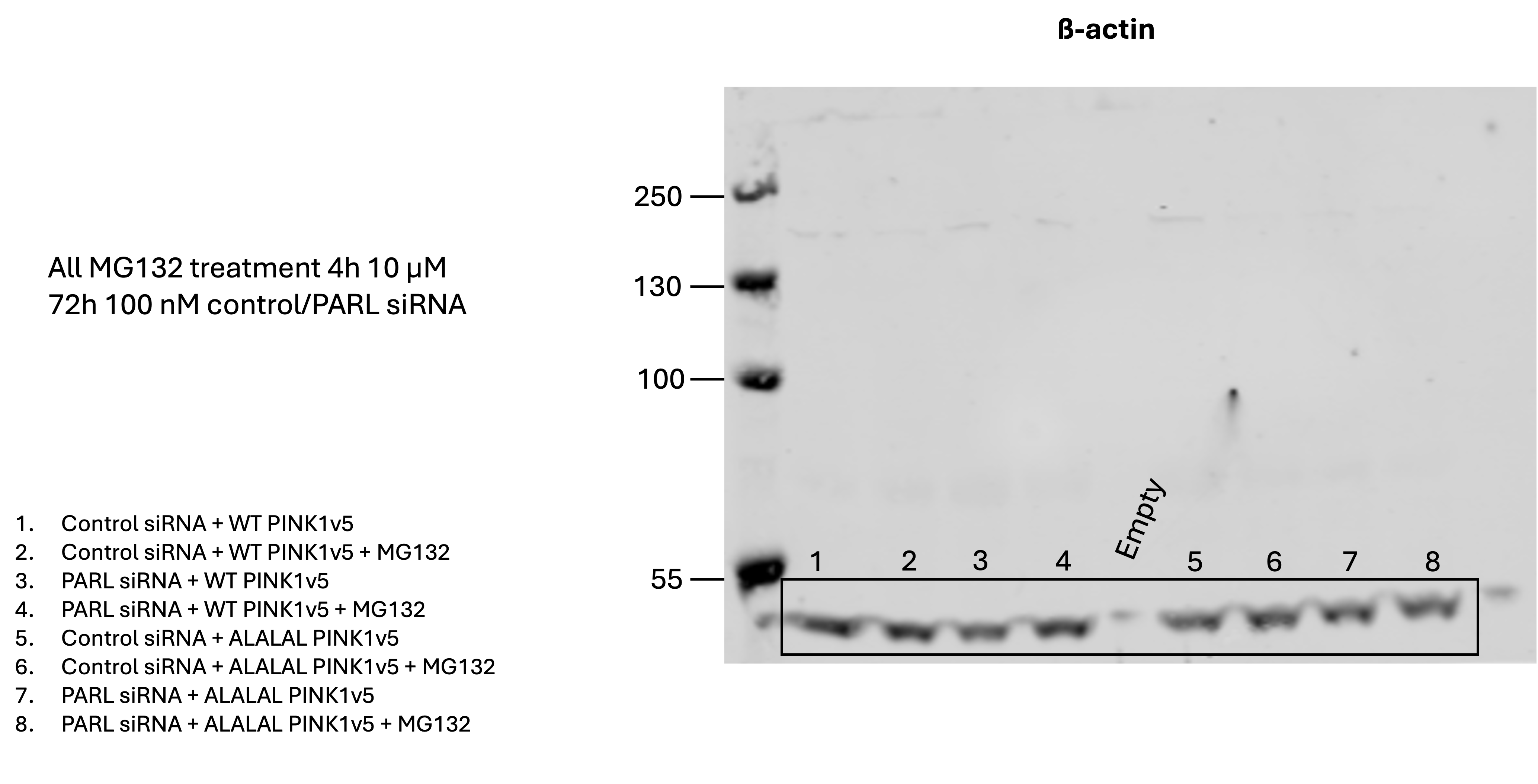

Supplement: Supplementary file 7 — Source data Fig. 8 [file 44318_2026_789_MOESM7_ESM.zip › Fig8/8c_blot_3.png]

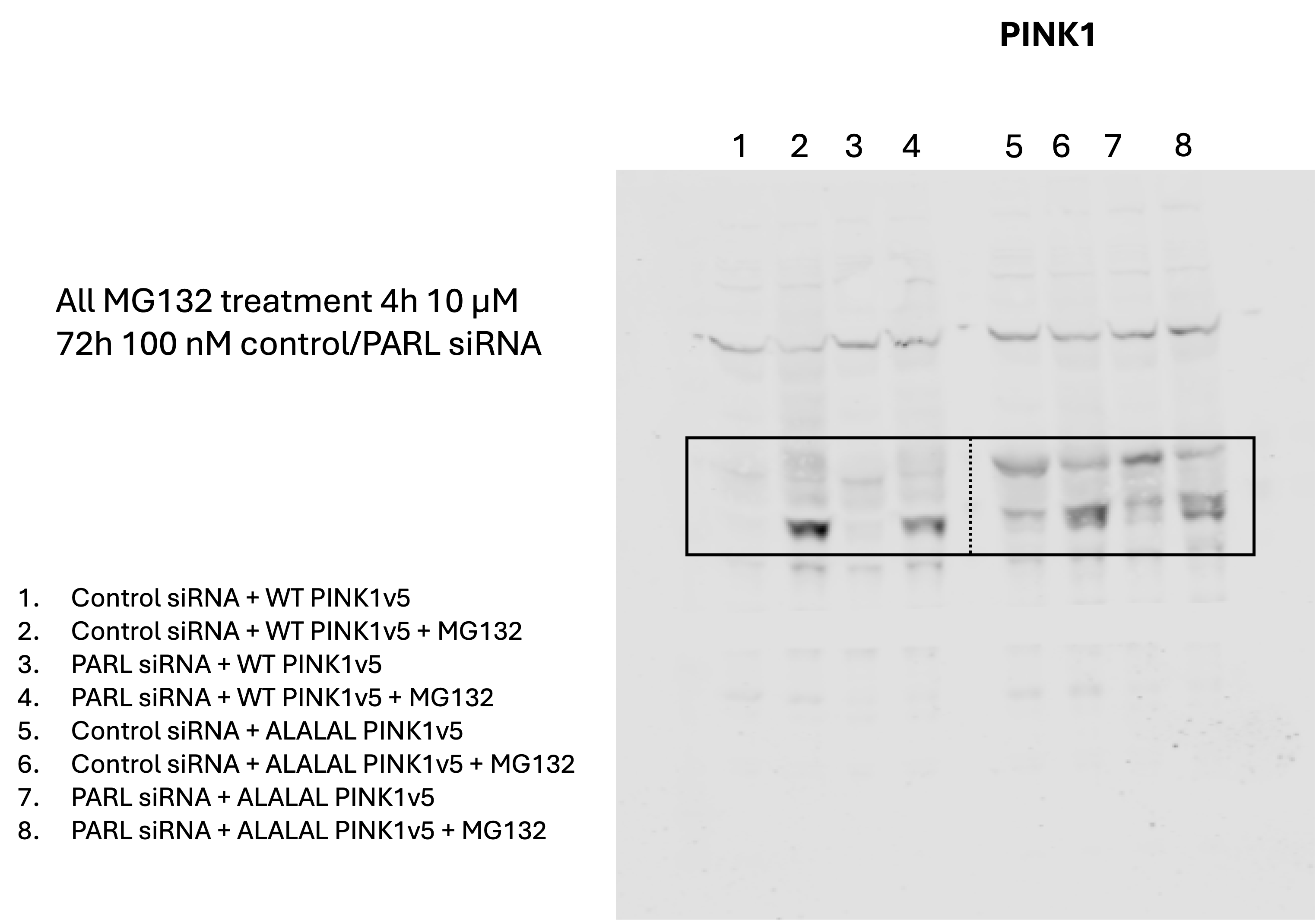

Supplement: Supplementary file 7 — Source data Fig. 8 [file 44318_2026_789_MOESM7_ESM.zip › Fig8/8c_blot_1.png]
