# Supplementary material for: Transmembrane domain switching controls PINK1 import and fate in mitochondria
Source: EMBO J. 2026 May 26;45(13):4447–68. doi: 10.1038/s44318-026-00789-x (PMC13324151; doi:10.1038/s44318-026-00789-x)
Supplement: Supplementary file 1 — Peer Review File [file 44318_2026_789_MOESM1_ESM.pdf]

THE EMBO JOURNAL

Trans-membrane-domain switching controls PINK1 import and fate in mitochondria

James Lorriman, Rhiannon Hughes, Adam Grieve, Robin Corey, and Ian Collinson

Corresponding author(s): Ian Collinson (ian.collinson@bristol.ac.uk) , Robin Corey (robin.corey@bristol.ac.uk), Adam Grieve (adam.grieve@bristol.ac.uk)

Review Timeline:

Submission Date:	6th Dec 24
Editorial Decision:	17th Feb 25
Revision Received:	13th Oct 25
Editorial Decision:	9th Dec 25
Appeal Received:	2nd Feb 26
Editorial Decision:	17th Mar 26
Revision Received:	26th Mar 26
Accepted:	1st Apr 26

Editor: William Teale

Transaction Report:

(Note: With the exception of the correction of typographical or spelling errors that could be a source of ambiguity, letters and reports are not edited. Depending on transfer agreements, referee reports obtained elsewhere may or may not be included in this compilation. Referee reports are anonymous unless the Referee chooses to sign their reports.)

1st Editorial Decision 17th Feb 2025

Dear Ian,

Thank you again for the submission of your manuscript entitled " Mitochondrial Import or Cleavage of PINK1 Controlled by Trans-Membrane-Domain Structural Plasticity". I have now received three referees' reports, which are copied to the bottom of this message. I would also like to apologise once more for the unusually long time the refereeing process has taken.

As you will see, all referees agree that the work is timely the topic is important; however, all referees shared similar concerns. Accordingly, the model that you present will need to be given a sounder experimental basis if this work is to be published in EMBO Journal.

An appreciable list of alternative models that may be also consistent with your data are suggested by the reviewers; therefore you will need to present data that exclude these. I am concerned that such additional experiments may not be feasible within a reasonable time-frame, and it may be in your best interests to submit the study elsewhere. Our usual revision time of three months is used as a guideline, not a deadline; manuscripts frequently take longer to revise. Once you and your team have had time to digest these reports, I recommend that we discuss the referees' comments further and go over our next steps on Zoom. In the meantime, I am prepared formally to invite you to submit a revised version of the manuscript.

I should add that it is The EMBO Journal policy to allow only a single major round of revision and that it is therefore important to resolve these concerns at this stage. I believe the concerns of the referees are reasonable and addressable, but please contact me if you have any questions, need further input on the referee comments or if you anticipate any problems in addressing any of their points. Please, follow the instructions below when preparing your manuscript for resubmission.

I would also like to point out that as a matter of policy, competing manuscripts published during this period will not be taken into consideration in our assessment of the novelty presented by your study ("scooping" protection). We have extended this 'scooping protection policy' beyond the usual 3 month revision timeline to cover the period required for a full revision to address the essential experimental issues. Please contact me if you see a paper with related content published elsewhere to discuss the appropriate course of action.

When preparing your letter of response to the referees' comments, please bear in mind that this will form part of the Review Process File, and will therefore be available online to the community. For more details on our Transparent Editorial Process, please visit our website: https://www.embopress.org/page/journal/14602075/authorguide#transparentprocess

Again, please contact me at any time during revision if you need any help or have further questions.

Thank you very much again for the opportunity to consider your work for publication. I look forward to your revision.

Best regards,

William

William Teale, Ph.D.
Editor
The EMBO Journal

When submitting your revised manuscript, please carefully review the instructions below and include the following items:

1) a .docx formatted version of the manuscript text (including legends for main figures, EV figures and tables). Please make sure that the changes are highlighted to be clearly visible.

2) individual production quality figure files as .eps, .tif, .jpg (one file per figure).

3) a .docx formatted letter INCLUDING the reviewers' reports and your detailed point-by-point response to their comments. As part of the EMBO Press transparent editorial process, the point-by-point response is part of the Review Process File (RPF), which will be published alongside your paper.

4) a complete author checklist, which you can download from our author guidelines (https://wol-prod-cdn.literatumonline.com/pb-assets/embo-site/Author Checklist%20-%20EMBO%20J-1561436015657.xlsx). Please insert information in the checklist that is also reflected in the manuscript. The completed author checklist will also be part of the RPF.

5) Please note that all corresponding authors are required to supply an ORCID ID for their name upon submission of a revised

manuscript.

6) We require a 'Data Availability' section after the Materials and Methods. Before submitting your revision, primary datasets produced in this study need to be deposited in an appropriate public database, and the accession numbers and database listed under 'Data Availability'. Please remember to provide a reviewer password if the datasets are not yet public (see https://www.embopress.org/page/journal/14602075/authorguide#datadeposition). If no data deposition in external databases is needed for this paper, please then state in this section: This study includes no data deposited in external repositories. Note that the Data Availability Section is restricted to new primary data that are part of this study.

Note - All links should resolve to a page where the data can be accessed.

7) When assembling figures, please refer to our figure preparation guideline in order to ensure proper formatting and readability in print as well as on screen:
http://bit.ly/EMBOPressFigurePreparationGuideline

Please remember: Digital image enhancement is acceptable practice, as long as it accurately represents the original data and conforms to community standards. If a figure has been subjected to significant electronic manipulation, this must be noted in the figure legend or in the 'Materials and Methods' section. The editors reserve the right to request original versions of figures and the original images that were used to assemble the figure.

8) For data quantification: please specify the name of the statistical test used to generate error bars and P values, the number (n) of independent experiments (specify technical or biological replicates) underlying each data point and the test used to calculate p-values in each figure legend. The figure legends should contain a basic description of n, P and the test applied. Graphs must include a description of the bars and the error bars (s.d., s.e.m.).

9) We would also encourage you to include the source data for figure panels that show essential data. Numerical data can be provided as individual .xls or .csv files (including a tab describing the data). For 'blots' or microscopy, uncropped images should be submitted (using a zip archive or a single pdf per main figure if multiple images need to be supplied for one panel). Additional information on source data and instruction on how to label the files are available at .

10) We replaced Supplementary Information with Expanded View (EV) Figures and Tables that are collapsible/expandable online (see examples in https://www.embopress.org/doi/10.15252/embj.201695874). A maximum of 5 EV Figures can be typeset. EV Figures should be cited as 'Figure EV1, Figure EV2" etc. in the text and their respective legends should be included in the main text after the legends of regular figures.

- For the figures that you do NOT wish to display as Expanded View figures, they should be bundled together with their legends in a single PDF file called *Appendix*, which should start with a short Table of Content. Appendix figures should be referred to in the main text as: "Appendix Figure S1, Appendix Figure S2" etc. See detailed instructions regarding expanded view here: .

- Additional Tables/Datasets should be labelled and referred to as Table EV1, Dataset EV1, etc. Legends have to be provided in a separate tab in case of .xls files. Alternatively, the legend can be supplied as a separate text file (README) and zipped together with the Table/Dataset file.

11) Our journal encourages inclusion of *data citations in the reference list* to directly cite datasets that were re-used and obtained from public databases. Data citations in the article text are distinct from normal bibliographical citations and should directly link to the database records from which the data can be accessed. In the main text, data citations are formatted as follows: "Data ref: Smith et al, 2001" or "Data ref: NCBI Sequence Read Archive PRJNA342805, 2017". In the Reference list, data citations must be labeled with "[DATASET]". A data reference must provide the database name, accession number/identifiers and a resolvable link to the landing page from which the data can be accessed at the end of the reference. Further instructions are available at .

Further instructions for preparing your revised manuscript:

Please make sure you upload a letter of response to the referees' comments together with the revised manuscript.

Please also check that the title and abstract of the manuscript are brief, yet explicit, even to non-specialists.

When assembling figures, please refer to our figure preparation guideline in order to ensure proper formatting and readability in print as well as on screen:
https://bit.ly/EMBOPressFigurePreparationGuideline
See also guidelines for figure legends: https://www.embopress.org/page/journal/14602075/authorguide#figureformat

IMPORTANT: When you send the revision we will require
- a point-by-point response to the referees' comments, with a detailed description of the changes made (as a word file).
- a word file of the manuscript text.
- individual production quality figure files (one file per figure)
- a complete author checklist, which you can download from our author guidelines (https://www.embopress.org/page/journal/14602075/authorguide).
- Expanded View files (replacing Supplementary Information)
Please see out instructions to authors
https://www.embopress.org/page/journal/14602075/authorguide#expandedview

Please remember: Digital image enhancement is acceptable practice, as long as it accurately represents the original data and conforms to community standards. If a figure has been subjected to significant electronic manipulation, this must be noted in the figure legend or in the 'Materials and Methods' section. The editors reserve the right to request original versions of figures and the original images that were used to assemble the figure.

Further information is available in our Guide For Authors: https://www.embopress.org/page/journal/14602075/authorguide

We realize that it is difficult to revise to a specific deadline. In the interest of protecting the conceptual advance provided by the work, we recommend a revision within 3 months (11th Jul 2022). Please discuss the revision progress ahead of this time with the editor if you require more time to complete the revisions. Use the link below to submit your revision:

https://emboj.msubmit.net/cgi-bin/main.plex

Instructions for preparing your revised manuscript:

Please make sure you upload a letter of response to the referees' comments together with the revised manuscript.

Please also check that the title and abstract of the manuscript are brief, yet explicit, even to non-specialists.

When assembling figures, please refer to our figure preparation guideline in order to ensure proper formatting and readability in print as well as on screen:
https://bit.ly/EMBOPressFigurePreparationGuideline
See also guidelines for figure legends: https://www.embopress.org/page/journal/14602075/authorguide#figureformat

At EMBO Press we ask authors to provide source data for the main manuscript figures. Our source data coordinator will contact you to discuss which figure panels we would need source data for and will also provide you with helpful tips on how to upload and organize the files.

IMPORTANT: When you send the revision we will require
- a point-by-point response to the referees' comments, with a detailed description of the changes made (as a word file).
- a word file of the manuscript text.
- individual production quality figure files (one file per figure)
- a complete author checklist, which you can download from our author guidelines (https://www.embopress.org/page/journal/14602075/authorguide).
- Expanded View files (replacing Supplementary Information)
Please see out instructions to authors
https://www.embopress.org/page/journal/14602075/authorguide#expandedview
- a Reagents and Tools Table as part of the Methods section, which can be downloaded from our author guidelines (https://www.embopress.org/page/journal/14602075/authorguide#structuredmethods)

Please remember: Digital image enhancement is acceptable practice, as long as it accurately represents the original data and conforms to community standards. If a figure has been subjected to significant electronic manipulation, this must be noted in the figure legend or in the 'Materials and Methods' section. The editors reserve the right to request original versions of figures and the original images that were used to assemble the figure.

Further information is available in our Guide For Authors: https://www.embopress.org/page/journal/14602075/authorguide

We realize that it is difficult to revise to a specific deadline. In the interest of protecting the conceptual advance provided by the work, we recommend a revision within 3 months (18th May 2025). Please discuss the revision progress ahead of this time with the editor if you require more time to complete the revisions. Use the link below to submit your revision:

https://emboj.msubmit.net/cgi-bin/main.plex

Referee #1:

Research on mitochondrial biogenesis and the integration of mitochondria into global cellular stress response networks is experiencing an utmost exciting period of time with a high frequency of groundbreaking studies reporting conceptually novel and unexpected findings. For example, the molecular models of the import machinery mediating the translocation of nuclear encoded proteins at the level of the mitochondrial inner membrane via the TIM23 complex has been revolutionized by elegant combinations of structural and biochemical studies recently. Around the same time an innovative new translocation assay to monitor mitochondrial import was introduced into the field that is built on the translocation-dependent formation of a luminescent split reporter protein. Some of the first results obtained with the new assay were in marked contrast to canonical views of the mitochondrial presequence import pathway. One surprising finding was that the import process may be characterized by two rate-limiting steps: first, transport across the outer membrane via the TOM complex and, second, initiation of inner membrane translocation via the TIM23 complex. Thus, these data raised doubts about the physiological significance of TOM-TIM23 super-complex formation, as it had been observed for decades to occur upon the experimental generation of arrested translocation intermediates. Notably, these findings have not been independently verified (to the best of my knowledge) and are controversially discussed in the field, like usual when a central dogma is challenged. However, who else than researchers studying mitochondrial biogenesis should know better from recent experience that dogmas can eventually fall?

In the presented manuscript, Lorriman et al. have used the luminescent split-reporter assay to study import of the PINK1 kinase into mitochondria under different conditions. PINK1 accumulation at mitochondria is an important stress-dependent trigger for induction of mitophagy and other responses. In healthy mitochondria PINK1 is translocated to inner membrane where it is cleaved by the rhomboid protease PARL priming the protein for proteasome-dependent degradation. In mitochondria with diminished import capability, e.g. through depolarization or induced by mutations affecting the import machinery, PINK1 will no longer be exposed to PARL and remain bound to a specific interface of the TOM complex to enhance its kinase activity. The authors claim that PINK1 import into mitochondria of permeabilized mammalian cells exhibits a two-step kinetic with distinct passages through TOM und TIM23, like they have suggested before for a different (matrix-targeted) model protein. These steps appear to be experimentally separable using split-reporter constructs placed at different positions of the PINK1 precursor in combination with intermembrane space- or matrix-localized complementary protein portion to restore fluorescence within a given compartment. A similar assay is used to test the previously formulated hypothesis that under fully energized conditions a fraction of PINK1 may not be cleaved by PARL, but rather undergo full translocation into the mitochondrial matrix to fulfill additional functions within this compartment. The authors then refer to the recently solved structure of the membrane-embedded Tim23-Tim17 protein import channel and use AlphaFold2 software to model putative molecular interactions at its lateral exit interface formed by Tim17 with substrate (PINK1) and potential partner proteins, like the previously suggested lateral gate keeper of the TIM23 complex, the Mgr2/ROMO1 protein. Alternative association of the lateral exit site with PARL would bring the PINK1 into a perfect position for proteolytic cleavage, while Mgr2/ROMO1 would shield the PINK1 preprotein from PARL. Molecular modelling revealed to possible conformations of the PINK1 transmembrane segment at Tim17, differing in their alpha-helix and beta-sheet content and favor the recruitment of either Mge2/ROMO1 or PARL, respectively, to trigger either matrix-translocation or cleavage and further degradation. Thus, a sensitive conformational equilibrium of the PINK1 transmembrane segment would determine the ultimate fate of the kinase. Notably, only a few of the hypotheses derived from this elegant model were tested experimentally, e.g. through mutational analysis, and thereby confirmed. Overall, this is a very exciting and elegant study that deserves attention of researchers from different areas of biochemistry and cell biology. However, this referee is convinced that the presentation and discussion of the paper must be substantially improved prior to publication in the EMBO Journal. Moreover, additional experiments will be required to substantiate the strong and far-reaching conclusions.

I apologize in advance, if I am being rude, but already in the introduction, several explanations provided by the authors remind me on a bull in a china shop.
- The choice of references is unconventional and certainly biased, neglecting most of the canonical literature. The two-step import mechanism via first TOM and then TIM23 is presented as a generally excepted model rather than the minority opinion that is certainly still is.
- Moreover, the novel recent considerations regarding the molecular nature of the protein-conducting (half-)channel in the TIM23 complex are over-simplified by stating that any possible matrix-translocation channel must definitely contain other proteins forming just another type of a canonical transmembrane pore. Possible translocation along a Tim17-half channel / phospholipid interface is completely neglected here, although exciting and elegant models have been developed. I suspect that the "mandatory pore" assumption is made for strategical reasons to justify the strict need for a competitive interaction of Mgr2/ROMO1 and other proteins with the Tim17-half channel later on.

- Finally, exciting recent insights into the accumulation of PINK1 at a TOM / TIM23 interface and its putative activation mechanism as well as the potential contribution of OMA1 to PINK1 biogenesis and processing are more or less completely neglected throughout the manuscript. Certainly, space is limited and neither introduction nor discussion should resemble a short review. But all these aspects are highly relevant for the detailed modelling of TIM23 / PINK1 /ROMO1 / PARL behavior later in the manuscript and cannot be ignored.

It is unclear to me, if the authors believe that the behavior of their model preprotein and the exposure of its different portions to distinct mitochondrial sub-compartments documents specific features of PINK1 or can be generalized to conventional precursor proteins imported into mitochondria via a stop-transfer mechanisms? If the latter is the case, an inner membrane-sorted control substrate with an N-terminal TMD should have been used for comparison. The authors occasionally refer to ACP1 as a control, the import of which they have studied in a previous publications. But for general mechanistic conclusions here regarding mitochondrial protein import and inner membrane sorting this is not enough.

I do not understand how the authors want to explain that split reporter constructs in the both N-terminus and C-terminus of PINK1 exhibit the same response kinetics? Can import across TOM in principle be initiated from either side the precursor (although less efficient from the C-terminus, therefore less total amount)? What is the negative control here for some random diffusion processes? What happens if the split-reporter is placed at the C-terminus of a cytosolic protein (bound to the surface of mitochondria)?

Importantly, the formation of the full-length luminescent reporter may well represent an artificial trapping mechanism. Ligand-induced protein folding is a generally accepted natural import mechanism for mitochondrial intermembrane space proteins, e.g. via the MIA pathway. The author must provide suitable controls that interrogate the potential contribution of such artificial trapping.

The data suggesting that a portion of PINK1 is imported into the mitochondrial matrix in fully energized, healthy mitochondria are overall convincing. This referee wonders, if the complete luminescent reporter protein could also be formed in PARL cleaves the LDD variant of PINK1, because the cut-off N-terminal part may at least transiently be expose to the matrix (as depicted e.g. in figure 1 of this manuscript. Or is this fragment rapidly degraded?

The molecular modelling data appears convincing as far as this referee can judge. The influence of PARL binding on the conformation of the PINK1 transmembrane segment is very interesting and may probably inspire similar studies in related fields. However, in some critical parts essential experimental verifications are missing. For example, the localization of the PINK1 transmembrane domain within a potential TIM23-PARL super-complex should be addressed by a minimum of site-specific crosslinking analysis, e.g. using engineered cysteine residues. As this manuscript makes very strong and distinct mechanistic claims of far-reaching importance, such verifications, at least in a minimal form, must be within the scope of this study.

I am confused by the experiments shown in parts of figure 4. If I am not completely misunderstanding something, the authors here claim that the N-terminal MTS is not critical for PINK1 import into mitochondria. A crucial importance of the MTS for PINK1 import and function at mitochondria has been shown several times before in vivo and in vitro. How do the authors explain their findings? (If the experiment is meant to shown something else, then a better explanation is necessary.)

It has been shown before in Ieva et al. (2014) that Mgr2 in the yeast system preferentially interacts with negatively charged amino acid residues flanking the transmembrane segment of a the inner membrane-sorted mitochondrial model preproteins used in that study. This fact should be acknowledged and discussed.

In general, it must be emphasized again that this manuscript contains a lot of interesting and thoroughly generated modeling data, but is falling short on experimental verification. This referee does not want to push the authors into a certain experimental direction that may be too difficult for them, but wherever possible additional experimental data should be provided to support the conclusions. Just to give one additional example: If Mgr2 / ROMO1 and PARL compete for the same (or overlapping) binding site(s) at the TIM23 complex, overexpression of either one in cells should affect this equilibrium and thus the outcome of the relevant MitoLuc assays for PINK1 import.

Finally, I strongly recommend to shorten and tone down the discussion.

Referee #2:

Mitochondria consist of two distinct membranes, the outer and inner membranes. Since 99% of mitochondrial precursors are synthesized on the cytosolic ribosomes, they must cross or be inserted into outer and/or inner membranes. However, the detailed mechanisms by which the precursors are transported to their destinations are still unknown. In particular, the PINK1 import pathway is not only unique, but also important for understanding of the elimination of dysfunctional mitochondria. PINK1 is a mitochondrial kinase, the gene product of which is mutated in familial Parkinson's disease. So far, it is known that, in healthy mitochondria, the PINK1 precursor is degraded in the proteasomes after cleavage by PARL, the rhomboid protease localized in

the inner mitochondrial membrane, and a minute amount of PINK1 is targeted to the matrix where it phosphorylates NdufA10, a subunit of respiratory chain complex I. However, how the PARL protease recognizes PINK1 in the inner membrane and how a portion of PINK1 crosses the inner membrane remains unknown. In this study, Lorriman et al. demonstrate that a portion of PINK1 reaches the mitochondrial matrix using MitoLuc assay that the authors have previously established. They also used an Alpha Fold (AF) structure model to construct a PINK1 intermediate complex with PARL, Tim17, and Tim23. While the overall model proposed by the authors is interesting, the predicted structures of AF have not been tested experimentally. The authors' models are not beyond the realm of the models. The reviewer believed that they need to demonstrate their model with a molecular biology strategy. Otherwise, the authors' manuscript is difficult to be accepted in the EMBO J.

Main comments
1) Since multiple pieces of information about the localization of PINK1 are contained in the N-terminal region including MTS, TM, OMS, PARL cleavage and others, it cannot be ruled out the possibility that the insertion of the peptide sequence pep86 altered the original localization information.

2) The authors state "in contrast to IMS import, passage into the matrix is sensitive to Antimycin A and Oligomycin (page 8)," but why is the inhibitory effect so small? The authors need to find a condition where DDL is not transported into the matrix at all, either by complete loss of membrane potential or by knockdown of the TIMM23 pathway by siRNA or other means. Otherwise, PINK1 transport to the matrix cannot be precisely evaluated.

3) In the authors' experiments in which a portion if PINK1 is transported to the matrix (Figure 3), the authors should show that PINK1 import to the matrix is unique, using as controls precursors (for example, TIMM50, TIMM21, MIC60) that are exclusively inserted into IMM via a stop-transfer pathway.

4) The authors' AF model that PARL associates with the TIM23 complex and ROMO1 dissociates from the TIM23 complex must be experimentally demonstrated.

5) Figure S5: The authors quantified the amount of PINK1 localized to the IMS by MitoLuc assay. However, the authors should show that i11S is exclusively localized to the IMS. Similarly, it is necessary to show that m11S is exclusively localized to matrix.

6) Figure 4g, h: The result that PINK1 mutants lacking MTS are still transported to matrix is very surprising and may change the conventional pathway of mitochondrial protein import. In order to prove this, the authors should test in more detail. For example, does PINK1 localize to the matrix even when PINK1 mutants lacking MTS are stably expressed in cells?

7) The authors' model that PINK1-PARL interaction is weakened by I111S substitution and that C92 and C96 of PINK1 enhance the sensitivity to oxidation needs to be shown experimentally.

Minor Comments
The following papers should be cited as a reference.
Okatsu et al. 2015 JCS doi: 10.1242/jcs.161000
Yamano and Youle 2013 Autophagy doi: 10.4161/auto.24633

Referee #3:

Lorriman et al. investigate the import of the mitochondrial kinase PINK1, primarily under healthy conditions. They emphasize that it is well established that PINK1 is imported into healthy mitochondria via the TOM and TIM complexes, where it is subsequently cleaved by PARL and degraded through retro-translocation to the cytosol. However, the current models do not account for the possibility that PINK1's kinase domain may be fully imported into the inter membrane space (IMS) to go to the matrix. The authors set out to re-examine PINK1's import into healthy mitochondria, with a particular focus on its ability to access both the IMS and the matrix. To achieve this, they purified full-length human PINK1 and adapted a previously established real-time cell assay (MitoLuc) to track mitochondrial import. The authors modified the assay by adding pep86 fragments at different regions of PINK1, along with a large fragment of split luciferase (11S) into the cells in either the matrix or IMS. They also compared PINK1 to ACP1, a matrix-bound protein, to provide a strong comparison control. The authors first demonstrate that PINK1 can be imported into the inner membrane space through two rate-limiting steps. Notably, they find that disruption of the membrane potential does not significantly impact the transport of PINK1 into the IMS. Continuing with this methodology, the authors reveal an exciting finding, that PINK1 can be fully imported into the matrix, which has not been widely accepted in the literature. They further explore the mechanism behind how PINK1's import occurs through structural modeling with AlphaFold2 and MD simulations. Their models predict that ROMO1 overlays the predicted binding site of PARL, suggesting that PINK1 binds either PARL or ROMO1, but not both simultaneously, indicating a new alternative pathway for PINK1 import. Additionally, they show that the transmembrane domain (TMD) of PINK1 forms an α-helix rather than an α/β-hybrid structure when interacting with ROMO1, as opposed to PARL, suggesting a structural plasticity and a layer of complexity to PINK1. Additionally, through various deletions of regions in PINK1, they demonstrate that the TMD is the crucial region necessary for matrix import. Importantly, the study highlights that the three glutamic acid residues in PINK1, which are known to be important for PINK1

accumulation, may play a key role in matrix import. The paper also shows that the Parkinson's disease mutation (I111S) impacts matrix import, further linking the findings to the disease. Finally, the authors use mutagenesis to find that disrupting the α/β-hybrid formation favors matrix import, suggesting that manipulating the TMD structure could influence the trafficking of PINK1.

Overall, the paper sheds light on how PINK1 is imported into mitochondria, as well as novel insights into the role of both PARL and ROMO1 for PINK1 import using an original methodology (MitoLuc). With recent advances in this field stressing that PINK1 and the TIM23 complex now interact under damaged conditions (see Akabane et al 2023 as well as Eldeeb et al 2024, which should have been cited), this paper is timely. The paper for the most part was well-written (with a few parts that need clarifications as indicated below). However, the AlphaFold2 prediction are over-interpreted and there is no experimental validation for these predicted models. Here are the main points that the authors should address.

1) In the introduction, the authors neglect to mention three important studies that are highly relevant to this work, and somewhat contradict their findings. The first one is Filipuzzi et al (2017, Nat Chem Biol), where the authors show that stendomycin, which blocks TIM23-dependent import, does not alter the normal PINK1 processing. The other two report that PINK1 binds to the TIM23 complex (TIM23, TIM17a/b, TIM50) upon mitochondrial depolarization, and in fact are part of the 800 kDa complex formed by PINK1 (Akabane et al 2023, Cell Rep; Eldeeb et al 2024, PNAS). However, the latter did not identify TIM44 nor ROMO1 in their proteomics data. How do the authors reconcile these data? This should be at the very least discussed.

2) Another important shortcoming of this manuscript is the overinterpretation of the AlphaFold2 results. First, a low score (high error) region of the PAE plot has nothing to do with an interaction being "transient"; AlphaFold2 does not provide any information on affinity. All it means is that AlphaFold2 is unable to predict the interaction between PINK1 and PARL, and thus it is pointless to analyze the interaction interface between the two. Secondly, the PAE plot is also very poor between PARL and the TIM23 complex, as well as PINK1 with the TIM23 complex, so these interactions should also not be interpreted. I would remove Figure 4 entirely, unless experimental validation is brought forward to support these models. Likewise, there is no experimental evidence supporting the findings from the MD simulations, thus I would remove Figures 5 and 6, they are too speculative. As an aside, a key component of the TIM complex that has been shown to have an important interaction with PINK1 is TIM50. TIM50 is a receptor part of the complex important for pre-protein import. It is not mentioned in the study, but has come up in mass-spectrometry analysis of the PINK1-TOM-TIM complex (Akabane et al 2023, Cell Rep; Eldeeb et al 2024, PNAS). Authors should comment on if TIM50 has a key role in their proposed model.

3) The placement of the 2nd pep86 fragment (between the three "Glu" residues and residue 150) is problematic in the light of more recent structures of PINK1 and work on the role of the N-terminal extension (NTE) (see Rasool et al 2022, Mol Cell; Kakade et al 2022, Open Biol). In the structure of TcPINK1 from Rasool et al, it is very clear that the N-terminal extension (equivalent to residue 120-150 in human PINK1) forms an helix that binds the C-terminal extension. More recently, the same groups showed that the NTE-CTE binds TOM20 and is important for the folding of PINK1 (Eldeeb et al. 2024, PNAS; Raimi et al 2024, Sci Adv). Thus, by inserting the pep86 fragment within the NTE, this will affect the ability of the kinase to fold, which in turn may affect PINK1's import properties. The authors should consider placing the pep86 fragment at a different position, perhaps after the NTE. Furthermore, it is not clear at all where the pep86 fragment is inserted... the methods should be explicit about where the pep86 fragment is insert in all three constructs. Another key point to address: it would be useful to show that upon addition of the pep86 fragment into PINK1, PINK1 is still functional and able to phosphorylate ubiquitin or parkin.

4) We are given an SDS-page gel to show purified human PINK1 (Supplemental figure 2). How do we know that this product is PINK1, and not an E. coli chaperone that co-purifies with PINK1? Human PINK1 is notoriously difficult from the E. coli, and I'm surprised the authors have been able to obtain so much protein... the elution should be analyzed by mass spectrometry to confirm that the primary product is PINK1. It would also be important to clarify which variant of human PINK1 we are being shown in this figure as it has not been specified in the figure legend nor labelled anywhere.

5) It is worth highlighting a key part of this study is the difference between the 11S locations. 11S has two key locations, in the matrix and in the inner membrane space (IMS). However, the authors do not show data to prove that the matrix and IMS 11S are properly localized. The addition of this figure in supplemental would be important to stress that the 11S have been properly localized to strengthen the results of this study.

6) Three key deletions were done in the study. The deletion of PINK1 from regions 1-50, the deletion from regions 1-91 and lastly the deletion of region 1-150. Based off of these deletions, it was concluded that only the deletion from regions 1-150 caused there to be a full abolishment of import into the matrix and the authors suggest this is because of the transmembrane domain. To further stress these results, and that it is not because of the OMS and MTS, I suggest deleting the segment of PINK1 that pertains only to its transmembrane domain, to say with confidence it is only the transmembrane domain having this impact and not because a large portion of the protein is being deleted.

7) The authors mutate the three glutamic residues of PINK1 (E112, 113, 117) to alanine, as well as make a Parkinson-disease mutation in PINK1 (I111S), shown in Figure 7. However, there lacks a strong disease-relevance to this study. Although it is stated that I111S is a disease mutation that was analyzed, other mutations would be interesting to study as well with this import model, since PINK1's relevance of Parkinson's Disease is highlighted several times in the paper. One that comes to mind is R98W, which is present in the transmembrane domain of PINK1. It would be interesting to know if this has any relevance and if it

could change the plasticity if this region. Any other disease mutations that are relevant to the key areas of PINK1 for import can also be added, as it would increase the human health relevance of the study.

Minor points to address (wording of text, formatting of figures etc...)

Abstract Page 1, line 14: "Failure of this process, in compromised mitochondria, leads to kinase activation at the surface and ultimately mitophagy" - In this sentence the authors are trying to say that if PINK1 is not cleaved by PARL, it will accumulate and cause mitophagy. This is PINK1's function under damaged state. However, by using the words "failure of this process". It makes it seem like PINK1 is not working properly. I suggest, removing this and starting with "in compromised mitochondria", to avoid confusion to the reader.

Fig 1B - The organization of the figure is confusing for the reader and someone not familiar with PINK1 import pathway. I suggest dividing it into two panels: one for compromised mitochondria and one for functional mitochondria. This information will be easier to digest for the reader and it is crucial since it is the starting figure of the paper.

Introduction, page 2, line 21: The authors do a great job describing the import of protein into the mitochondria, which is often complex to put into words. However, on line 21, they mention mtHsp70, without mentioning it is a core part of the PAM complex. Since this paper is all about import into the mitochondria, I think it would be crucial to mention the PAM complex in the introduction, even briefly. Could be added in line 26 as well, where ever the authors feel fit to add it in.

Introduction, page 3, line 27: OMD is written instead of OMS.

Page 6, line 13: "save for the position of the active reporter" ... This is unclear and I am not sure what this sentence means. I think it should be re-worded to be a bit clearer to readers.

Figure 7: The figure legend in figure 7 does not match the figure. On line 6, it says that figure a-b shows IMS data and d-F shows matrix data, but it is the opposite in the figure shown.

Introduction, page 4, line 15: One of the "also" present in this sentence can be removed, since it is repetitive.

Introduction, page 5, line 2: There is a comma missing. "Given uncertainties about these alternative destinations of PINK1, ..."

Introduction, page 5, line 18: There is a spelling mistake here. "The inner-mmebrane" need to be changed to "the inner-membrane".

Page 6, line 8-9: Location of where the pep86 was located was described to readers, but reason for why these locations were chosen were not explained until line 31. I recommend moving the explanation in the same area, as this will be less confusing for readers and make it clear right away why these positions were chosen, and where they are exactly. I also recommend adding a table into the paper, that readers can refer back to, as a quick way for readers to remember what every variant of PINK1 means, as it can be confusing to remember.

Conclusion line 7: Wording is not the clearest in this sentence. I would re-phrase to put an emphasis on that fact that this study showed the transmembrane domain is important.

Conclusion line 25 : the word "retro-translocation" is used to describe PINK1 not being imported into the matrix of cleaved by PARL, however I find this mis-leading to the readers. I suggest re-wording this sentence, because when I read retro-translocation, I automatically think of PINK1 going back to the cytosol for degradation.

Figure 9: Similar to a previous comment, to make it more clear to readers, the damaged state of PINK1 should be in a separate panel, especially because this study focuses more on healthy state conditions. I suggest one panel for ROMO1 and PARL graphics and another panel for compromised mitochondria graphics, to make it less confusing to the reader when looking at the figure.

Dr William Teale
Scientific Editor *EMBO J.*

13th October, 2025

Ian Collinson

Professor of Biochemistry

School of Biochemistry
University Walk
Bristol BS8 1TD
United Kingdom

Tel.: +44 780 95-39701
E-mail: ian.collinson@bristol.ac.uk

Dear Dr Teale,

We would like to thank you and the reviewers for the considerable time and effort spent evaluating our manuscript. The reviews and our subsequent discussions have been extremely valuable in helping us improve and refine the manuscript. We believe that the revised version is much better, and hope you will agree, and now consider it suitable for publication in *The EMBO Journal*.

As discussed, we have conducted additional experiments and amended the text extensively to address the concerns of the reviewers and where clarifications were required. Below we have summarised our general approach (as discussed with you) for the preparation of the new submission, followed by a point-by-point response to each of the three reviewers.

The concerns of the reviewers could be roughly separated into three sections, which we outline below, along with our general response:

1. **Validation of the import assay**.

Some of the reviewers' concerns were around the protein import assay. The *MitoLuc* import assay has been published in three peer reviewed methods papers [1–3] . This includes being deployed in the research reports describing the analysis of import into isolated mitochondria [4], and into mitochondria within intact cells [5], as we also do in the enclosed manuscript. Therefore, we feel the reliability and validity of the import assay has already been firmly established, and doing so again would not be a good use of time and resources. With that being said, we have addressed specific concerns where they arise in the point-by-point below.

2. **Validation of the structural models**.

The reviewers rightly requested some validation of the structural modelling. In this we fully agree. Therefore, we have provided an extended section on this very point, which includes additional comparison to experimental structures and a new analysis figure that demonstrates the model's correct prediction of the PINK1 cleavage site based on

proximity to the PARL active site (see point-by-point below). We also include some new analysis metrics of the AlphaFold models including ipSAE metrics, released since initial submission of the manuscript.

3. **Testing the model for PINK1**.

We are in agreement with the reviewers and the editor that the pressing issue was to provide further support for our model for PINK1's alternative destinations, which if true will have far reaching implications for the role of PINK1 in the regulation of mitochondrial quality control and function—critical for health and disease. Therefore, most of our efforts, in terms of the provision of new experimental data, were focussed on strengthening this aspect of the manuscript.

To this end, we have included a new results section incorporating additional text and figure panels. In summary, we have further explored the concept that PINK1 can be diverted to either the outer-membrane or the matrix, respectively for the regulation of quality control (mitophagy) or mitochondrial structure and function. We also sought to provide new data in support of these alternative pathways being determined by the conformation of PINK1's trans-membrane domain. The results both support and enrich the story. They show that inhibiting matrix import favours the alternative pathway—cleavage by PARL, retro-translocation and outer-membrane delivery. While, we could also show, as predicted, stabilisation of the TMD as an α-helix (α/β-hybrid destabilisation) favours import and prevents cleavage by PARL.

Sincerely

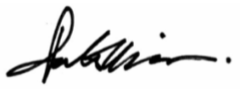

Ian Collinson, on behalf of all the authors.

Point-by-point response:

Referee #1:

Research on mitochondrial biogenesis and the integration of mitochondria into global cellular stress response networks is experiencing an utmost exciting period of time with a high frequency of groundbreaking studies reporting conceptually novel and unexpected findings. For example, the molecular models of the import machinery mediating the translocation of nuclear encoded proteins at the level of the mitochondrial inner membrane via the TIM23 complex has been revolutionized by elegant combinations of structural and biochemical studies recently. Around the same time an innovative new translocation assay to monitor mitochondrial import was introduced into the field that is built on the translocation-dependent formation of a luminescent split reporter protein. Some of the first results obtained with the new assay were in marked contrast to canonical views of the mitochondrial presequence import pathway. One surprising finding was that the import process may be characterized by two rate-limiting steps: first, transport across the outer membrane via the TOM complex and, second, initiation of inner membrane translocation via the TIM23 complex. Thus, these data raised doubts about the physiological significance of TOM-TIM23 super-complex formation, as it had been observed for decades to occur upon the experimental generation of arrested translocation intermediates. Notably, these findings have not been independently verified (to the best of my knowledge) and are controversially discussed in the field, like usual when a central dogma is challenged. However, who else than researchers studying mitochondrial biogenesis should know better from recent experience that dogmas can eventually fall?

In the presented manuscript, Lorriman et al. have used the luminescent split-reporter assay to study import of the PINK1 kinase into mitochondria under different conditions. PINK1 accumulation at mitochondria is an important stress-dependent trigger for induction of mitophagy and other responses. In healthy mitochondria PINK1 is translocated to inner membrane where it is cleaved by the rhomboid protease PARL priming the protein for proteasome-dependent degradation. In mitochondria with diminished import capability, e.g. through depolarization or induced by mutations affecting the import machinery, PINK1 will no longer be exposed to PARL and remain bound to a specific interface of the TOM complex to enhance its kinase activity. The authors claim that PINK1 import into mitochondria of permeabilized mammalian cells exhibits a two-step kinetic with distinct passages through TOM und TIM23, like they have suggested before for a different (matrix-targeted) model protein. These steps appear to be experimentally separable using split-reporter constructs placed at different positions of the PINK1 precursor in combination with intermembrane space- or matrix-localized complementary protein portion to restore fluorescence within a given compartment. A similar assay is used to test the previously formulated hypothesis that under fully energized conditions a fraction of PINK1 may not be cleaved by PARL, but rather undergo full translocation into the mitochondrial matrix to fulfil additional functions within this compartment. The authors then refer to the recently solved structure of the membrane-embedded Tim23-Tim17 protein import channel and use AlphaFold2 software to model

putative molecular interactions at its lateral exit interface formed by Tim17 with substrate (PINK1) and potential partner proteins, like the previously suggested lateral gate keeper of the TIM23 complex, the Mgr2/ROMO1 protein. Alternative association of the lateral exit site with PARL would bring the PINK1 into a perfect position for proteolytic cleavage, while Mgr2/ROMO1 would shield the PINK1 preprotein from PARL. Molecular modelling revealed two possible conformations of the PINK1 transmembrane segment at Tim17, differing in their alpha-helix and beta-sheet content and favor the recruitment of either Mgr/ROMO1 or PARL, respectively, to trigger either matrix-translocation or cleavage and further degradation. Thus, a sensitive conformational equilibrium of the PINK1 transmembrane segment would determine the ultimate fate of the kinase. Notably, only a few of the hypotheses derived from this elegant model were tested experimentally, e.g. through mutational analysis, and thereby confirmed. Overall, this is a very exciting and elegant study that deserves attention of researchers from different areas of biochemistry and cell biology.

However, this referee is convinced that the presentation and discussion of the paper must be substantially improved prior to publication in the EMBO Journal. Moreover, additional experiments will be required to substantiate the strong and far-reaching conclusions.
The revised manuscript has been substantially improved throughout the text and through the incorporation of new data.

I apologize in advance, if I am being rude, but already in the introduction, several explanations provided by the authors remind me on a bull in a china shop.
- The choice of references is unconventional and certainly biased, neglecting most of the canonical literature. The two-step import mechanism via first TOM and then TIM23 is presented as a generally excepted model rather than the minority opinion that is certainly still is.
The introduction has been amended incorporating also more citations concerning the 'canonical literature'. We have slightly expanded the one-step versus multi-step model comparison. Interesting recent work from the Li lab [6] is supportive of the latter, suggesting that channel formation by Mgr2 and Tim17 is one such step, which directly relates to our model.

- Moreover, the novel recent considerations regarding the molecular nature of the protein-conducting (half-)channel in the TIM23 complex are over-simplified by stating that any possible matrix-translocation channel must definitely contain other proteins forming just another type of a canonical transmembrane pore. Possible translocation along a Tim17-half channel / phospholipid interface is completely neglected here, although exciting and elegant models have been developed. I suspect that the "mandatory pore" assumption is made for strategical reasons to justify the strict need for a competitive interaction of Mgr2/ROMO1 and other proteins with the Tim17-half channel later on.
The recent paper highlighted above adds further support the channel composition (Tim17 and Mgr2/Romo1) [6]. Nevertheless, we have described the current thinking on the role of the protein/lipid interface in protein sorting and cite a new review article which discusses the channel conundrum in greater depth.

- Finally, exciting recent insights into the accumulation of PINK1 at a TOM / TIM23 interface and its putative activation mechanism as well as the potential contribution of OMA1 to PINK1 biogenesis and processing are more or less completely neglected throughout the manuscript. Certainly, space is limited and neither introduction nor discussion should resemble a short review. But all these aspects are highly relevant for the detailed modelling of TIM23 / PINK1 /ROMO1 / PARL behavior later in the manuscript and cannot be ignored.
As noted, being mindful of space limitations, we have addressed this point with the citation of an excellent recent review (2024) from the Youle lab and have mentioned the OMA1 story.

It is unclear to me, if the authors believe that the behavior of their model preprotein and the exposure of its different portions to distinct mitochondrial sub-compartments documents specific features of PINK1 or can be generalized to conventional precursor proteins imported into mitochondria via a stop-transfer mechanisms? If the latter is the case, an inner membrane-sorted control substrate with an N-terminal TMD should have been used for comparison. The authors occasionally refer to ACP1 as a control, the import of which they have studied in a previous publications. But for general mechanistic conclusions here regarding mitochondrial protein import and inner membrane sorting this is not enough.
We believe the matrix-import pathway of PINK1 is likely to be similar to that of a canonical precursor. We have clarified this point the text, but at this stage were unable to elaborate the mechanistic detail. A deeper analysis of PINK1's matrix import alongside canonical precursors, with stop-transfer sequences (or otherwise), would certainly be very interesting. However, it is beyond the scope of this paper and the time available for its revision. Moreover, at this stage we have only been able to fit the IMS import data (but not the matrix import data) to a kinetic model. So, quite a bit more work needs to be done towards the analysis of comparative mechanistic details of matrix import.

I do not understand how the authors want to explain that split reporter constructs in the both N-terminus and C-terminus of PINK1 exhibit the same response kinetics? Can import across TOM in principle be initiated from either side the precursor (although less efficient from the C-terminus, therefore less total amount)? What is the negative control here for some random diffusion processes? What happens if the split-reporter is placed at the C-terminus of a cytosolic protein (bound to the surface of mitochondria)?
Unlike previously using isolated mitochondria [4], the kinetics of import within intact cells, as described here, could only be achieved for import into the IMS, but not the matrix. This is because the behaviour in this context is too complex (there are more than 2 rate-limiting steps) and challenging to determine and fit to a model. The overall rates observed do indeed look similar, which means that translocation *per se* is not the limiting factor, but something else (*e.g.* initiation at the inner-membrane, TIM17-Romo1 channel formation, *etc.*) We feel that an extended discussion on this point would distract the reader.

We feel that our control for non-specific import is robust: PINK1(Δ150) without its N-terminal region of which no trace can be detected in the matrix (Figure 4h).

Importantly, the formation of the full-length luminescent reporter may well represent an artificial trapping mechanism. Ligand-induced protein folding is a generally accepted natural import mechanism for mitochondrial intermembrane space proteins, e.g. via the MIA pathway. The author must provide suitable controls that interrogate the potential contribution of such artificial trapping.
For a number of reasons, we do not consider this to be a concern. The appearance of the pep86 (small luciferase) in the IMS or matrix occurs by virtue of the endogenous import machinery; not because of the large fragment (11S) residing there. Once transport has occurred it becomes available for binding, forms an active luciferase and generates a signal. Even if the binding event enhances translocation then it will not contribute to the assay readout as there are no additional 11S binding sites.

One possibility is that association with the 11S prevents reverse-translocation and thereby artificially enhances the import activity. Though there is no evidence that the import machinery undergoes retro-transport. Quite the contrary; if this were the case then blocking import with a precursor-DHFR fusion would prevent forward transport and allow backward transport, bringing about precursor dissociation. However, this does not occur.

Furthermore, when pep86 is on the C-terminus we know that artificial trapping will have no effect because translocation across the membrane is already complete, after which reverse translocation cannot occur.

As noted above, the import assays have already been through peer review, so we feel that an extended retrospective of the assay would be distractive.

The data suggesting that a portion of PINK1 is imported into the mitochondrial matrix in fully energized, healthy mitochondria are overall convincing. This referee wonders, if the complete luminescent reporter protein could also be formed in PARL cleaves the LDD variant of PINK1, because the cut-off N-terminal part may at least transiently be expose to the matrix (as depicted e.g. in figure 1 of this manuscript. Or is this fragment rapidly degraded?
Good point, from the data we cannot say. If so, this would result in an artificially increase in the matrix signal for LDD, which does not amount to a serious concern. A note has been added to the text to mention this possibility.

The molecular modelling data appears convincing as far as this referee can judge. The influence of PARL binding on the conformation of the PINK1 transmembrane segment is very interesting and may probably inspire similar studies in related fields. However, in some critical parts essential experimental verifications are missing. For example, the localization of the PINK1 transmembrane domain within a potential TIM23-PARL super-complex should be addressed by a minimum of site-specific crosslinking analysis, e.g. using engineered cysteine residues. As this manuscript makes very strong and distinct mechanistic claims of far-reaching importance, such verifications, at least in a minimal form, must be within the scope of this study.

We agree that it is important that the model we presented are verified, therefore we have extended this section of the results. The reviewer suggests that we undertake a series of targeted cysteine disulphide mapping, which, for several reasons, are impractical:

(1) Cysteine-cysteine cross-linking is good for the verification of static assemblies, however, the models we present capture single states of a dynamic assembly. In reality, PINK1 will be translocating through the inner-membrane for cleavage or matrix import. So, any attempts at cysteine scanning would prove messy and likely inconclusive.
(2) The biological system is too complex. The cross-linking would need to be done in live system—isolated mitochondria or mitochondria within permeabilised cells (required to promote PINK1 association for cleavage/import). Moreover, mitochondria are quite cysteine rich, which would create an overwhelmingly high background signal. Therefore, the analysis would be very unlikely to deliver clean and conclusive results.
(3) The main region of the model that we are interested is the highly conserved TMD, which as we have shown is very sensitive to amino acid substitution. Therefore, the cysteine scanning could well affect the behaviour of the structure in unexpected ways.
(4) Finally, from a pragmatic perspective the time required for this type of analysis would go way beyond the permissible time for a revision.

Therefore, we devised a better approach. Instead of using the model to predict location of neighbouring residues for cysteine mutagenesis and disulphide prediction, we used the model to predict the location of the PINK1 cleavage. The analysis, shown as a new figure panel, correctly identifies the cleavage site of PINK1, information which the AlphaFold modeller was not privy to. We also employ a new analysis method, ipSAE, to assess the confidence of this region of the model—added to the results text and incorporated into a new supplementary Figure S7d.

I am confused by the experiments shown in parts of figure 4. If I am not completely misunderstanding something, the authors here claim that the N-terminal MTS is not critical for PINK1 import into mitochondria. A crucial importance of the MTS for PINK1 import and function at mitochondria has been shown several times before in vivo and in vitro. How do the authors explain their findings? (If the experiment is meant to shown something else, then a better explanation is necessary.)
Correct, our data show that the MTS and indeed all the N-terminal residues up to 91 are not required for import. The TMD, however, is required for both import and cleavage (new Figure 8b). Both models of the cleavage and import complex are consistent with this finding. This is now clearly stated in the text.

It has been shown before in Ieva et al. (2014) that Mgr2 in the yeast system preferentially interacts with negatively charged amino acid residues flanking the transmembrane segment of a the inner membrane-sorted mitochondrial model preproteins used in that study. This fact should be acknowledged and discussed.

Thank you. We have cited the Ieva *et al* study to highlight the interaction of Mgr2 with negatively charged residues flanking the transiting trans-membrane domain. This paper supports the presented model of the matrix import complex, involving interactions of PINK1's TMD with ROMO1.

In general, it must be emphasized again that this manuscript contains a lot of interesting and thoroughly generated modeling data, but is falling short on experimental verification. This referee does not want to push the authors into a certain experimental direction that may be too difficult for them, but wherever possible additional experimental data should be provided to support the conclusions. Just to give one additional example: If Mgr2 / ROMO1 and PARL compete for the same (or overlapping) binding site(s) at the TIM23 complex, overexpression of either one in cells should affect this equilibrium and thus the outcome of the relevant MitoLuc assays for PINK1 import.
Excellent point. We have conducted new experiments and now show that this equilibrium can be shifted in both directions. If we inhibit import then cleavage is enhanced (Figure 8a). While, if we destabilise the interaction with PARL then import is stimulated (Figure 8b-d)

Finally, I strongly recommend to shorten and tone down the discussion.
The discussion has been shortened and toned down.

Referee #2:

Mitochondria consist of two distinct membranes, the outer and inner membranes. Since 99% of mitochondrial precursors are synthesized on the cytosolic ribosomes, they must cross or be inserted into outer and/or inner membranes. However, the detailed mechanisms by which the precursors are transported to their destinations are still unknown. In particular, the PINK1 import pathway is not only unique, but also important for understanding of the elimination of dysfunctional mitochondria. PINK1 is a mitochondrial kinase, the gene product of which is mutated in familial Parkinson's disease. So far, it is known that, in healthy mitochondria, the PINK1 precursor is degraded in the proteasomes after cleavage by PARL, the rhomboid protease localized in the inner mitochondrial membrane, and a minute amount of PINK1 is targeted to the matrix where it phosphorylates NdufA10, a subunit of respiratory chain complex I. However, how the PARL protease recognizes PINK1 in the inner membrane and how a portion of PINK1 crosses the inner membrane remains unknown. In this study, Lorriman et al. demonstrate that a portion of PINK1 reaches the mitochondrial matrix using MitoLuc assay that the authors have previously established. They also used an Alpha Fold (AF) structure model to construct a PINK1 intermediate complex with PARL, Tim17, and Tim23. While the overall model proposed by the authors is interesting, the predicted structures of AF have not been tested experimentally. The authors' models are not beyond the realm of the models. The reviewer believed that they need to demonstrate their model with a molecular biology strategy. Otherwise, the authors' manuscript is difficult to be accepted in the EMBO J.
The revised MS now has an expanded section on model validation (see also above, and point-by-point below).

Main comments

1) Since multiple pieces of information about the localization of PINK1 are contained in the N-terminal region including MTS, TM, OMS, PARL cleavage and others, it cannot be ruled out the possibility that the insertion of the peptide sequence pep86 altered the original localization information.

The location of the pep86 sequence was intentionally located in low conservation areas of PINK1. The pep86 insertion of LDD and DLD are actually quite far away from the key N-terminal region including the TMD. To emphasise and clarify this point we now include the annotated modified sequence of PINK1 deployed in the MitoLuc assay as a main figure panel (Figure 1c). We have also indicated where the pep86 insertion sites are located on the schematic structural depiction of PINK1 (Figure 4f).

Another point to note here is that import can proceed when the N-terminal region containing the first site of insertion (of LDD) is deleted. Therefore, the first insertion site is very unlikely to affect import. Furthermore, the final pep86 reporter (of DDL) is on the very C-terminus, which cannot possibly have an impact on import.

Finally, our previous peer reviewed method papers on the luciferase assay deployed in bacterial secretion and in MitoLuc found no indication whatsoever of interference by the pep86 incorporation.

2) The authors state "in contrast to IMS import, passage into the matrix is sensitive to Antimycin A and Oligomycin (page 8)," but why is the inhibitory effect so small? The authors need to find a condition where DDL is not transported into the matrix at all, either by complete loss of membrane potential or by knockdown of the TIMM23 pathway by siRNA or other means. Otherwise, PINK1 transport to the matrix cannot be precisely evaluated.

The effect of antimycin A and oligomycin (AO) was far from complete because they are less effective, in this particular system, at reducing the membrane potential [2]. We had to make do with this approach because the more effective ionophore CCCP is incompatible with the luciferase assay. Though the effect of AO is lower than ideal, it is significant. Words to this effect have been added to the text.

Knocking down the TIM23 pathway would almost certainly create a whole bunch of confusing off-target effects. The requested negative control 'where DDL is not transported into the matrix at all' has been achieved by deletion of PINK1's N-terminal region 1-150 and shown in Figure4h.

3) In the authors' experiments in which a portion if PINK1 is transported to the matrix (Figure 3), the authors should show that PINK1 import to the matrix is unique, using as controls precursors (for example, TIMM50, TIMM21, MIC60) that are exclusively inserted into IMM via a stop-transfer pathway.

We have already provided robust validation of our MitoLuc assay as part of our previous 3 peer reviewed method papers. Further, the construction, characterisation and comparative analysis of the suggestions outline above would take far longer than the time line of a revision. Moreover, a detailed comparison of PINK1 import versus canonical

stop-transfer precursors, also as noted above, would take us beyond the scope of this study.

4) The authors' AF model that PARL associates with the TIM23 complex and ROMO1 dissociates from the TIM23 complex must be experimentally demonstrated.
We have included an extended section in the results (including new experiments) that tests the model of a dynamic association with either ROMO1 or PARL, respectively for import or cleavage. We now show that this equilibrium can be shifted in both directions. We show that destabilisation of the import complex with an inhibitor enhances cleavage (Figure 8a), while perturbation of PINK1's interaction with PARL stimulates import (Figure 8b-d).

In the meantime, a recent paper from the Li lab [6] suggests that the reversible association of Mgr2 (yeast ROMO1) with Tim17, for channel formation, is an inherent part of the transport mechanism. This is consistent with our model involving a dynamic reversible interaction with of Tim17 with either ROMO1 or PARL (Figure 9).

5) Figure S5: The authors quantified the amount of PINK1 localized to the IMS by MitoLuc assay. However, the authors should show that i11S is exclusively localized to the IMS. Similarly, it is necessary to show that m11S is exclusively localized to matrix.
The targeting of 11S was achieved by the cox8a mitochondrial pre-sequence—tried and tested for the successful targeting of non-mitochondrial proteins to the matrix. The resulting luciferase signal associated with precursor import was previously shown to be characteristic of matrix import (dependence on the membrane potential and sensitivity to matrix import inhibitors) [2]. We know that there is residual 11S in the cytosol, which we have been able to quench with the 'dark'-peptide bound to GST (GST-Dark), but it is not possible for residual 11S in the IMS to be quenched in this way. Nevertheless, there is compelling circumstantial evidence that any matrix destined 11S in the IMS has limited impact. For example, we show that the C-terminus of the PINK1-3EA variant (DDL) can enter the IMS (Figure 7f), but not the matrix (Figure 7c). In the latter experiment, significant amounts of matrix targeted 11S in the IMS would have generated a false positive for matrix import, which was not the case. Therefore, the amounts of matrix directed 11S in the IMS must be marginal.

The IMS import assay, in contrast, has not been previously validated, so the reviewer was also right to query this point. For IMS targeting of 11S we deployed the Smac/DIABLO targeting sequence [7] . To verify its expected localisation we conducted a biochemical analysis of its mitochondrial distribution (Figure S1). The results are consistent with the processed and unprocessed versions of Smac/DIABLO targeted 11S being, respectively, detached and attached to the inner-membrane, but both in the IMS. Furthermore, the IMS signal is not sensitive to the antimycin A and oligomycin. If there was a significant proportion of 11S in the matrix, then there would be a corresponding sensitivity to these drugs, but this was not the case. Finally, and similar to the point made above, the luminescent signal liberated for entry of the variant PINK1-3EA variant (DDL) in the IMS confirms that the 11S is located in the IMS, rather than the matrix. If the latter were the case then no signal would have resulted.

Additional manuscript text, along with the new supplementary figures, have been incorporated into the revision package to summarise our confidence in the location of IMS and matrix targeted 11S.

6) Figure 4g, h: The result that PINK1 mutants lacking MTS are still transported to matrix is very surprising and may change the conventional pathway of mitochondrial protein import. In order to prove this, the authors should test in more detail. For example, does PINK1 localize to the matrix even when PINK1 mutants lacking MTS are stably expressed in cells?
The results do show that in the absence of the MTS import proceeds, but less efficiently. Furthermore, as import is abolished with (Δ1-150) but retained with Δ1-91, then 91-150 (containing the TMD) is necessary and sufficient (this is what we say in the results). This suggests a non-essential role in the refinement of targeting and translocation process. We were not particularly surprised by this finding as lots of mitochondrial proteins do not possess a cleavable N-terminal pre-sequence [8]. In those cases, the targeting information needs to be incorporated into the mature regions of the precursor; such as the TMD of PINK1. Words to this effect have been added to text.

7) The authors' model that PINK1-PARL interaction is weakened by I111S substitution and that C92 and C96 of PINK1 enhance the sensitivity to oxidation needs to be shown experimentally.
This is indeed an important line of inquiry and these experiments will be conducted for a follow up paper.

Minor Comments
The following papers should be cited as a reference.
Okatsu et al. 2015 JCS doi: 10.1242/jcs.161000 Yamano and Youle 2013 Autophagy doi: 10.4161/auto.24633
Done

Referee #3:

Lorriman et al. investigate the import of the mitochondrial kinase PINK1, primarily under healthy conditions. They emphasize that it is well established that PINK1 is imported into healthy mitochondria via the TOM and TIM complexes, where it is subsequently cleaved by PARL and degraded through retro-translocation to the cytosol. However, the current models do not account for the possibility that PINK1's kinase domain may be fully imported into the inter membrane space (IMS) to go to the matrix. The authors set out to re-examine PINK1's import into healthy mitochondria, with a particular focus on its ability to access both the IMS and the matrix. To achieve this, they purified full-length human PINK1 and adapted a previously established real-time cell assay (MitoLuc) to track mitochondrial import. The authors modified the assay by adding pep86 fragments at different regions of PINK1, along with a large fragment of split luciferase (11S) into the cells in either the matrix or IMS. They also compared PINK1 to ACP1, a matrix-bound

protein, to provide a strong comparison control. The authors first demonstrate that PINK1 can be imported into the inner membrane space through two rate-limiting steps. Notably, they find that disruption of the membrane potential does not significantly impact the transport of PINK1 into the IMS. Continuing with this methodology, the authors reveal an exciting finding, that PINK1 can be fully imported into the matrix, which has not been widely accepted in the literature. They further explore the mechanism behind how PINK1's import occurs through structural modeling with AlphaFold2 and MD simulations. Their models predict that ROMO1 overlays the predicted binding site of PARL, suggesting that PINK1 binds either PARL or ROMO1, but not both simultaneously, indicating a new alternative pathway for PINK1 import. Additionally, they show that the transmembrane domain (TMD) of PINK1 forms an α-helix rather than an α/β-hybrid structure when interacting with ROMO1, as opposed to PARL, suggesting a structural plasticity and a layer of complexity to PINK1. Additionally, through various deletions of regions in PINK1, they demonstrate that the TMD is the crucial region necessary for matrix import. Importantly, the study highlights that the three glutamic acid residues in PINK1, which are known to be important for PINK1 accumulation, may play a key role in matrix import. The paper also shows that the Parkinson's disease mutation (I111S) impacts matrix import, further linking the findings to the disease. Finally, the authors use mutagenesis to find that disrupting the α/β-hybrid formation favors matrix import, suggesting that manipulating the TMD structure could influence the trafficking of PINK1.

Overall, the paper sheds light on how PINK1 is imported into mitochondria, as well as novel insights into the role of both PARL and ROMO1 for PINK1 import using an original methodology (MitoLuc). With recent advances in this field stressing that PINK1 and the TIM23 complex now interact under damaged conditions (see Akabane et al 2023 as well as Eldeeb et al 2024, which should have been cited), this paper is timely. The paper for the most part was well-written (with a few parts that need clarifications as indicated below). However, the AlphaFold2 prediction are over-interpreted and there is no experimental validation for these predicted models. Here are the main points that the authors should address.
We have now cited those papers, along with the new article highlighting the structure of the PINK1 dimer at the outer-membrane sandwiched between 2 sets of TOM-complexes, and unexpectedly, VDAC [9].

Note also that we have included additional experimental validation of the model, addressing the concern above.

1) In the introduction, the authors neglect to mention three important studies that are highly relevant to this work, and somewhat contradict their findings. The first one is Filipuzzi et al (2017, Nat Chem Biol), where the authors show that stendomycin, which blocks TIM23-dependent import, does not alter the normal PINK1 processing.
We now show that inhibition of import by MB20 enhances the alternative pathway consistent with our dynamic model (new Figure 8c). While Filipuzzi *et al* show that inhibition of import through TIM23 by another inhibitor (stendomycin) does not seem to positively affect normal processing of PINK1—though it is quite difficult to be sure because only one blot relating to this result was shown, and MG132 does not appear to be used to visualise the PARL cleavage product.

The other two report that PINK1 binds to the TIM23 complex (TIM23, TIM17a/b, TIM50) upon mitochondrial depolarization, and in fact are part of the 800 kDa complex formed by PINK1 (Akabane et al 2023, Cell Rep; Eldeeb et al 2024, PNAS). However, the latter did not identify TIM44 nor ROMO1 in their proteomics data. How do the authors reconcile these data? This should be at the very least discussed.
Thank you, these papers have now been incorporated into our MS. False negatives are common place in proteomic studies; we suspect that Tim44 and ROMO1 could have fallen off upon extraction, affinity isolation and preparation for mass-spectrometry. Indeed, as discussed above we suspect that Mgr2/ROMO1 is subject to reversible association with the TIM23-complex[6] .

2) Another important shortcoming of this manuscript is the overinterpretation of the AlphaFold2 results. First, a low score (high error) region of the PAE plot has nothing to do with an interaction being "transient"; AlphaFold2 does not provide any information on affinity. All it means is that AlphaFold2 is unable to predict the interaction between PINK1 and PARL, and thus it is pointless to analyze the interaction interface between the two. Secondly, the PAE plot is also very poor between PARL and the TIM23 complex, as well as PINK1 with the TIM23 complex, so these interactions should also not be interpreted. I would remove Figure 4 entirely, unless experimental validation is brought forward to support these models. Likewise, there is no experimental evidence supporting the findings from the MD simulations, thus I would remove Figures 5 and 6, they are too speculative. As an aside, a key component of the TIM complex that has been shown to have an important interaction with PINK1 is TIM50. TIM50 is a receptor part of the complex important for pre-protein import. It is not mentioned in the study, but has come up in mass-spectrometry analysis of the PINK1-TOM-TIM complex (Akabane et al 2023, Cell Rep; Eldeeb et al 2024, PNAS). Authors should comment on if TIM50 has a key role in their proposed model.
We agree that AF2 does not report on affinity, and did not mean to imply this. In terms of the transience, we instead were referring firstly to a predicted increase in dynamics of the interface (as has been shown to correlate with PAE) as well as with a lack of experimental structures that would be available in the AF2 training set (which would decrease modelling confidence). To avoid confusion, we have removed this phrasing.

We respectfully disagree about removing Figures 4, 5 and 6, as we feel that these data offer considerable structural insights into the mechanism of activity, and are the best data we have *in lieu* of an experimental structural model (which we anticipate will be difficult to obtain). As described in the text, the use of molecular dynamics provides some degree of validation of the models.

To further support our AlphaFold models and MD data, we have expanded the section of structure validation, including new experiments. We have also introduced analysis using ipSAE (not available during the initial submission) to support the confidence of the PINK1-PARL model with respect to the beta-strand region. Once published we hope that the structures and their implications will be interrogated further and refined and modified accordingly

Regarding the PARL to TIM23-complex prediction, the predicted water channel forms between PARL and Tim17, which are chains A and C in the model. We have added a section to the text describing the confidence of this region (including an ipSAE score) with caveats about using caution not to overinterpret the data.

Most likely TIM50 will play a role, but as yet we do not have any experimental data ready for inclusion here. We think we could agree that further AF modelling of additional subunits will need additional empirical support.

3) The placement of the 2nd pep86 fragment (between the three "Glu" residues and residue 150) is problematic in the light of more recent structures of PINK1 and work on the role of the N-terminal extension (NTE) (see Rasool et al 2022, Mol Cell; Kakade et al 2022, Open Biol). In the structure of TcPINK1 from Rasool et al, it is very clear that the N-terminal extension (equivalent to residue 120-150 in human PINK1) forms an helix that binds the C-terminal extension. More recently, the same groups showed that the NTE-CTE binds TOM20 and is important for the folding of PINK1 (Eldeeb et al. 2024, PNAS; Raimi et al 2024, Sci Adv). Thus, by inserting the pep86 fragment within the NTE, this will affect the ability of the kinase to fold, which in turn may affect PINK1's import properties. The authors should consider placing the pep86 fragment at a different position, perhaps after the NTE.
See also response above (Referee #2, point 1). We looked at the structures showing how the N-terminal helix of PINK1 (NTE) contacts its C-terminal extension (CTE). The pep86 reporter was inserted into a non-conserved region and flanked by residues usually associated with high flexibility ...PG/P.... The insertion was then flanked by additional residues to enhance flexibility, as follows:

....PGSG-pep86-GSGP.......

For the purposes of illustration below we have shown the Human AF3 structure, which is in excellent agreement with the insect version (TcPINK1). The structure shows that the site of pep86 insertion of the 2nd pep86 (DLD; residues shown as spheres) within the N-terminal helix around 120-150 (hot pink) is actually not in contact with the CTE (blue)—

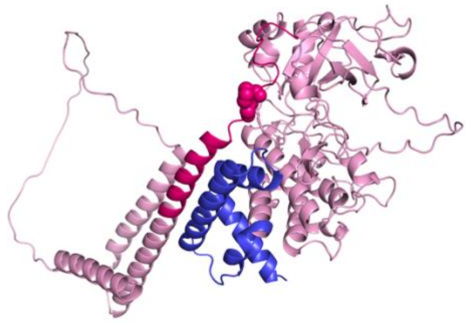

this is also apparent when looking at the Tc structure. Therefore, it is likely that the folded structure would tolerate the pep86 insertion.

Even if the final folded state is affected by this insertion then it would not be a problem for our experiments. PINK1 like all other precursors will be transported in an unfolded conformation. While the secondary structure will be maintained, so that the structure of the TMD will be recognised (α-helical or the α/β-hybrid form), the tertiary structural elements will be lost. All 3 pep86 sites are far away from the critical TMD so will not affect

its secondary structural conformation. Certainly also, perturbation of the interaction with the N- and C-terminal regions would have no impact on import activity, as they will not be in contact during import. Furthermore, even if folding is affected upon matrix entry, this event will occur after the appearance of pep86 in the IMS/matrix and therefore not affect the import signal.

Furthermore, it is not clear at all where the pep86 fragment is inserted... the methods should be explicit about where the pep86 fragment is insert in all three constructs.
The insertion sites were described in a supplemental figure (S1), which has now been promoted to the main figures (Figure 1c), and we have also described their whereabout in a structural schematic (Figure 4f).

Another key point to address: it would be useful to show that upon addition of the pep86 fragment into PINK1, PINK1 is still functional and able to phosphorylate ubiquitin or parkin.
All 3 pep86 sites are incorporated within non-conserved sites, not part of the of the kinase domain. So, in all likelihood, they would not affect PINK1's ability to phosphorylate ubiquitin/parkin. Moreover, this paper concerns matrix import and cleavage of PINK1, occurring during translocation of PINK, during which time the protein is unfolded (as discussed above). In this unfolded state the pep86 sites will be far away from the TMD of PINK1, and have no impact on its structural configuration (α-helical or α/β-hybrid)—critical for deciding its fate. Therefore, even in the unlikely event that pep86 insertion affects PINK1's kinase activity, they will still have no consequences for matrix-import or cleavage.

While we are interested in the behaviour of PINK1 at the outer-membrane, it is not really a feature of this paper, so new experiments in this direction would form parts of a follow-up story.

4) We are given an SDS-page gel to show purified human PINK1 (Supplemental figure 2). How do we know that this product is PINK1, and not an E. coli chaperone that co-purifies with PINK1? Human PINK1 is notoriously difficult from the E. coli, and I'm surprised the authors have been able to obtain so much protein... the elution should be analyzed by mass spectrometry to confirm that the primary product is PINK1. It would also be important to clarify which variant of human PINK1 we are being shown in this figure as it has not been specified in the figure legend nor labelled anywhere.
Full-length human PINK1 forms inclusion bodies when over-produced in *E. coli*. The major component of inclusion bodies is almost always insoluble aggregated recombinant protein, and this is the case for PINK1. The washed isolated inclusion bodies are already quite pure.

The protein is then solubilised from the inclusion bodies and further purified chromatographically. We know that the product of this purification, shown in the gel (Figure S2), is PINK1 because it was affinity purified by its Histag. Additionally, it results in the formation of an active luciferase when added to 11S—by virtue of its pep86 fragment (Figure S3 and others). No other protein in *E. coli* will do this. It also has the correct apparent molecular weight.

The protein was created as a fusion with SUMO at the N-terminus; this was the trick that enabled us to make the protein (it is indeed very tricky to make the full-length human protein recombinantly). The cleavage of the SUMO protein resulted in an expected reduction in the MW of the extracted protein to produce the band shown in Figure S2. The figure shows the SDS-PAGE analysis of the variant DDL (this information has now been added to the MS and supplemental figure legend). All of the other variants, in respect of their purification, behaved in the same way, so we decided not to show them.

Therefore, we know that the protein shown is of the right size, contains SUMO, a Ulp1 protease cleavage site (for SUMO removal), a Histag and the pep86 sequence, leaving no doubt about its identity as the recombinantly produced PINK1.

5) It is worth highlighting a key part of this study is the difference between the 11S locations. 11S has two key locations, in the matrix and in the inner membrane space (IMS). However, the authors do not show data to prove that the matrix and IMS 11S are properly localized. The addition of this figure in supplemental would be important to stress that the 11S have been properly localized to strengthen the results of this study.
Done; see response to Referee #2, point 5.

6) Three key deletions were done in the study. The deletion of PINK1 from regions 1-50, the deletion from regions 1-91 and lastly the deletion of region 1-150. Based off of these deletions, it was concluded that only the deletion from regions 1-150 caused there to be a full abolishment of import into the matrix and the authors suggest this is because of the transmembrane domain. To further stress these results, and that it is not because of the OMS and MTS, I suggest deleting the segment of PINK1 that pertains only to its transmembrane domain, to say with confidence it is only the transmembrane domain having this impact and not because a large portion of the protein is being deleted.
As the PINK1(Δ1-91) variant (Figure 1c) retains its ability to enter the matrix (Figure 4g) we can clearly conclude that neither the MTS nor the OMS are essential for matrix import.

7) The authors mutate the three glutamic residues of PINK1 (E112, 113, 117) to alanine, as well as make a Parkinson-disease mutation in PINK1 (I111S), shown in Figure 7. However, there lacks a strong disease-relevance to this study. Although it is stated that I111S is a disease mutation that was analyzed, other mutations would be interesting to study as well with this import model, since PINK1's relevance of Parkinson's Disease is highlighted several times in the paper. One that comes to mind is R98W, which is present in the transmembrane domain of PINK1. It would be interesting to know if this has any relevance and if it could change the plasticity if this region. Any other disease mutations that are relevant to the key areas of PINK1 for import can also be added, as it would increase the human health relevance of the study.
Excellent point. We have recently secured new funding to further explore more of the mutations associated with Parkinson’s Disease.

Minor points to address (wording of text, formatting of figures etc...)

Abstract Page 1, line 14: "Failure of this process, in compromised mitochondria, leads to kinase activation at the surface and ultimately mitophagy" - In this sentence the authors are trying to say that if PINK1 is not cleaved by PARL, it will accumulate and cause mitophagy. This is PINK1's function under damaged state. However, by using the words "failure of this process". It makes it seem like PINK1 is not working properly. I suggest, removing this and starting with "in compromised mitochondria", to avoid confusion to the reader.
Done.

Fig 1B - The organization of the figure is confusing for the reader and someone not familiar with PINK1 import pathway. I suggest dividing it into two panels: one for compromised mitochondria and one for functional mitochondria. This information will be easier to digest for the reader and it is crucial since it is the starting figure of the paper.
Done

Introduction, page 2, line 21: The authors do a great job describing the import of protein into the mitochondria, which is often complex to put into words. However, on line 21, they mention mtHsp70, without mentioning it is a core part of the PAM complex. Since this paper is all about import into the mitochondria, I think it would be crucial to mention the PAM complex in the introduction, even briefly. Could be added in line 26 as well, where ever the authors feel fit to add it in.
Done

Introduction, page 3, line 27: OMD is written instead of OMS.
Fixed

Page 6, line 13: "save for the position of the active reporter" ... This is unclear and I am not sure what this sentence means. I think it should be re-worded to be a bit clearer to readers.
Clarified

Figure 7: The figure legend in figure 7 does not match the figure. On line 6, it says that figure a-b shows IMS data and d-F shows matrix data, but it is the opposite in the figure shown.
Thank you, good spot!

Introduction, page 4, line 15: One of the "also" present in this sentence can be removed, since it is repetitive.
Done

Introduction, page 5, line 2: There is a comma missing. "Given uncertainties about these alternative destinations of PINK1, ..."
Done

Introduction, page 5, line 18: There is a spelling mistake here. "The inner-mmebrane" need to be changed to "the inner-membrane".

Fixed

Page 6, line 8-9: Location of where the pep86 was located was described to readers, but reason for why these locations were chosen were not explained until line 31. I recommend moving the explanation in the same area, as this will be less confusing for readers and make it clear right away why these positions were chosen, and where they are exactly.
Done

I also recommend adding a table into the paper, that readers can refer back to, as a quick way for readers to remember what every variant of PINK1 means, as it can be confusing to remember.
Done

Conclusion line 7: Wording is not the clearest in this sentence. I would re-phrase to put an emphasis on that fact that this study showed the transmembrane domain is important.
Done

Conclusion line 25 : the word "retro-translocation" is used to describe PINK1 not being imported into the matrix of cleaved by PARL, however I find this mis-leading to the readers. I suggest re-wording this sentence, because when I read retro-translocation, I automatically think of PINK1 going back to the cytosol for degradation.
Done

Figure 9: Similar to a previous comment, to make it more clear to readers, the damaged state of PINK1 should be in a separate panel, especially because this study focuses more on healthy state conditions. I suggest one panel for ROMO1 and PARL graphics and another panel for compromised mitochondria graphics, to make it less confusing to the reader when looking at the figure.
Done

REFERENCES:

[1] G.C. Pereira, W.J. Allen, D.W. Watkins, L. Buddrus, D. Noone, X. Liu, A.P. Richardson, A. Chacinska, I. Collinson, A High-Resolution Luminescent Assay for Rapid and Continuous Monitoring of Protein Translocation across Biological Membranes., Journal of Molecular Biology 431 (2019) 1689–1699. https://doi.org/10.1016/j.jmb.2019.03.007.

[2] H.I. Needs, J.S. Lorriman, G.C. Pereira, J.M. Henley, I. Collinson, The MitoLuc Assay System for Accurate Real-Time Monitoring of Mitochondrial Protein Import Within Mammalian Cells, J. Mol. Biol. 435 (2023) 168129. https://doi.org/10.1016/j.jmb.2023.168129.

[3] Hope.I. Needs, Y. Yan, N.M. Niemi, I. Collinson, The MitoLuc assay for the analysis of the mechanism of mitochondrial protein import, Methods Enzym. (2024). https://doi.org/10.1016/bs.mie.2024.07.033.

[4] H.C. Ford, W.J. Allen, G.C. Pereira, X. Liu, M.S. Dillingham, I. Collinson, Towards a molecular mechanism underlying mitochondrial protein import through the TOM and TIM23 complexes, Elife 11 (2022) e75426. https://doi.org/10.7554/elife.75426.

[5] H.I. Needs, E. Glover, G.C. Pereira, A. Witt, W. Hübner, M.P. Dodding, J.M. Henley, I. Collinson, Rescue of mitochondrial import failure by intercellular organellar transfer, Nat. Commun. 15 (2024) 988. https://doi.org/10.1038/s41467-024-45283-2.

[6] Y. Yang, S. Wang, G. Wang, Y. Lian, L. Xue, W. Jiang, Q. Guo, C. Song, L. Li, Dynamic TOM–TIM23 supercomplex directs mitochondrial protein translocation and sorting, Nat. Struct. Mol. Biol. (2025) 1–11. https://doi.org/10.1038/s41594-025-01662-x.

[7] L. Burri, Y. Strahm, C.J. Hawkins, I.E. Gentle, M.A. Puryer, A. Verhagen, B. Callus, D. Vaux, T. Lithgow, Mature DIABLO/Smac Is Produced by the IMP Protease Complex on the Mitochondrial Inner Membrane, Mol. Biol. Cell 16 (2005) 2926–2933. https://doi.org/10.1091/mbc.e04-12-1086.

[8] Y.S. Bykov, T. Flohr, F. Boos, N. Zung, J.M. Herrmann, M. Schuldiner, Widespread use of unconventional targeting signals in mitochondrial ribosome proteins, Embo J 41 (2022) e109519. https://doi.org/10.15252/embj.2021109519.

[9] S. Callegari, N.S. Kirk, Z.Y. Gan, T. Dite, S.A. Cobbold, A. Leis, L.F. Dagley, A. Glukhova, D. Komander, Structure of human PINK1 at a mitochondrial TOM-VDAC array, Science 388 (2025) 303–310. https://doi.org/10.1126/science.adu6445.

1st Revision - Editorial Decision 9th Dec 2025

Dear Ian,

Thank you for submitting your manuscript entitled 'Mitochondrial Import or Cleavage of PINK1 Controlled by Trans-Membrane-Domain Structural Plasticity' (EMBOJ-2024-119845) to our editorial office. We have now considered the study within our editorial team, and unfortunately come to the conclusion that we cannot offer publication in The EMBO Journal. On the way to this decision, I have engaged all reviewers in further discussions; I am, though, very sorry for the unusually long time this process has taken. As you will see from the reports of your revised version, Referee #1 remained positive; however, both Referee #3 and Referee #2 (in comments on the other two referee reports) maintained that without further testing with site-directing mutagenesis, they did not have sufficient confidence in the model to be able to support publication. I hoped that we could find a way through, but in the end I simply didn't have the support from the referees that I would have needed.

I am sorry that I cannot be more positive for The EMBO Journal on this occasion, but very much hope that you will not be discouraged from working with us at EMBO Press in the future.

Best wishes,

William

William Teale, PhD
Editor
The EMBO Journal
w.teale@embojournal.org

Referee #1:

The authors have made substantial efforts to carefully address all points raised by the reviewers. In particular, they provide convincing new experimental data in the revised version that support the validity of the structural models. Not all critical points could be resolved, e.g. the how the model of a transient protein-conducting channel of Tim17 and Mgr2/ROMO1 as the main passageway for protein import into the matrix can be reconciled with the fact that knockout mutants lacking Mgr2 or ROMO1, respecitvely, display only a minor matrix translocation phenotype. However, in my view these minor shortcoming are acceptable here, because overall the manuscript has much imoproved in the revision. This elegant study provides very interesting new insights into the mechanisms of mitochondrial protein sorting as well as PINK1 biology and will be of great interest for the broad readership of the EMBO Journal. With respect to the competitive situation in this vivid area of research, I recommend to accept this paper in the present form now.

Referee #3:

General comment.

Lorriman et al have submitted a revised version of the manuscript which addresses many of the points we raise. However, I remain unconvinced by the structural modeling, which was not validated in a substantial way. The addition of the MB20 experiments adds value, but there are still some flaws. Detailed comments are found below.

Mentioning the new PINK1-TOM structure is indeed important to mention now, thus we appreciate the authors adding it to their paper. However, when mentioning the structure be sure to mention it is VDAC2, as there are three isoforms of VDAC. This is not specified in the paper.

Here are the responses to each numbered points I raised previously.

1) The results with MB20 do strengthen the manuscript, but the immunoblot showing the PARL knockdown in Figure S14 is not convincing. We understand the PARL antibody is not good, but perhaps RT-PCR or even mass spec could be used to demonstrate PARL-KD.

2) The ipSAE is indeed an improvement on the ipTM score, which is particularly well suited for cases such as this, where only a small region in a protein (like the PINK1 peptide) binds to another protein (PARL). However, it remains a prediction, and not an experimental validation. Having performed the modelling myself, with both AF2 and AF3, I can also see that the top 5 models don't converge well, with mean rmsd > 5 Ang between the 5 models for the segment of PINK1 100-113, after superposing the PARL domain. This is not a good sign; it doesn't prove the interaction doesn't exist (AlphaFold is prone to false negative), but it

reduces our confidence in the model. Having written that, I would have less qualms about this if some experimental validation was provided (i.e. a mutagenesis to prove that a given interaction is important for the cleavage), but this was not provided for the PINK1-PARL interaction. The only new experiments that I can see are those with the ALALAL mutants in Figure 8, which improves the manuscript, but don't validate the structural model. I agree that the proximity of the PINK1 Phe104 to the active site serine in PARL suggests the model must be close to the real structure, but by presenting a single model of the structure, it provides a false sense of certainty about the structure. The authors should at least show an ensemble of the structure predicted by AlphaFold.

While the PINK1-PARL structural prediction is somewhat believable, the PARL:TIM23 supercomplex or PINK1-TIM23 are not. Again, the authors do acknowledge the speculative nature of these complexes, but it is misleading to show these structures as static models in Figure 4d,e, given that they are low confidence models. Furthermore, no PAE plots are shown for the PINK1-TIM23 complex. Unless the PAE plot confidently predicts an interaction between PINK1-TIM23, the structural model should be removed entirely. If there is a possible interaction, the authors must also demonstrate that the models converge. In additional, they must provide experimental validation of these models, using site-specific mutagenesis.

By the way, there is no figure legend description for Fig. S7e, and no legend description for the color code in Fig. S7d. Likewise, the Fig. S11 could better labeled. Subunits directly labelled on the figure would be a big help to get the key point of the figure across.

3) The authors are incorrect in their assumptions. Even though the NTE is far from the active site of PINK1, its interaction with TOM20 is critical for the activation, as disruption of this interaction abrogates PINK1's kinase activity. In turn, the folding (or lack) of PINK1 in the cytosol may affect the translocation kinetics of PINK1. While we understand that this paper is not concerned with PINK1 accumulation at the OMM, it should at least be stated that the function of PINK1 under damaged condition with the pep86 peptide was not investigated, and this in turn may affect PINK1's import kinetics.

4) Thanks for the clarification on the purification.

5) OK

6) Yes, we understood that, but then the authors should write on page 14: "Therefore, only the region between 91-150, including the TMD (containing PARL cleavage site) and the NTE, but not the OMS, are necessary and sufficient for association with the TIM23-complex and import." To make it clear that it's not just the TMD. I will also note that this contradicts previous results showing that deletion of the N-terminal MTS prevents PINK1 import into the matrix and renders PINK1 constitutively active (Okatsu et al 2015). The difference here may have to do with the point #3 above, i.e. that PINK1 folding (or misfolding by insertion of pep86) may affect import properties.

7) OK

Other comments:

8) Introduction: Line 26-27, page 4. It is worth mentioning OMA1 cleavage of PINK1 is induced under specific conditions that disrupt PINK1 accumulation (TOM7 deletion, OMS deletion, TIM deletion etc.). This is not clear in this new introduction.

9) On page 10, the authors added a new sentence "The more effective ionophore CCCP could not be used because of its incompatibility with the luciferase assay". This sentence is not accurate. To start with, antimycin A and oligomycin A are not protonophores; protonophores carry protons across membranes whereas antimycin A and oligomycin A are ETC inhibitors. It's also surprising to read that they are not efficient in depolarizing, given they have been used in numerous and achieve complete depolarization. What concentrations of antimycin A and oligomycin A are being used in this study? This should be specified, maybe insufficient concentrations were being used (but we cannot tell).

** As a service to authors, EMBO Press provides authors with the possibility to transfer a manuscript that one journal cannot offer to publish to another EMBO publication or the open access journal Life Science Alliance launched in partnership between EMBO Press, Rockefeller University Press and Cold Spring Harbor Laboratory Press. The full manuscript and if applicable, reviewers' reports, are automatically sent to the receiving journal to allow for fast handling and a prompt decision on your manuscript. For more details of this service, and to transfer your manuscript please click on Link Not Available. **

Please do not share this URL as it will give anyone who clicks it access to your account.

Ian Collinson

Professor of Biochemistry

School of Biochemistry
University Walk
Bristol BS8 1TD
United Kingdom

email: ian.collinson@bristol.ac.uk

Dr William Teale
Scientific Editor *EMBO J.*

30th January, 2026

Dear Dr Teale,

I am enclosing a rebuttal to the review of our paper: '*Mitochondrial Import or Cleavage of PINK1 Controlled by Trans-Membrane-Domain Structural Plasticity*' (EMBOJ-2024-119845), as agreed over email. The original manuscript was subject to a rigorous and largely positive review early last year. The referees' suggestions and concerns were mostly fair and constructive. Some of them could be addressed through clarification and straight forward experimentation, while others would have required more significant analyses that were impractical within the time frame of a revision. Some of the remarks were not necessarily helpful or valid. With all things being considered, we were invited to prepare a revised manuscript incorporating new experiments to address the major concerns. These were discussed in detail by video link, and a clear experimental plan was agreed in order to gain more support for the new model of PINK1 trafficking (Figure 9), and to further validate the structural models. It was acknowledged at that stage that the MitoLuc assay constitutes a *bona fide* and validated measure of mitochondrial import, as established in multiple peer reviewed papers. On this basis, it was agreed that the validity of the assay did not need to be addressed again here.

The new experiments we conducted were successful and their results were incorporated, together with further clarifications, into the revised manuscript alongside a point-by-point explanatory response (13-10-25, included further below). We also explained why some of the suggestions were impractical or unlikely to help. For instance, as previously explained, disulphide cross-linking experiments to validate the structural models would not be a suitable approach. Instead, we presented an independent analysis revealing that the predicted model and subsequent Molecular Dynamics simulations accurately recapitulate the interaction between the PARL active site and PINK1's cleavage site. The analysis precisely identified the cleavage site in PINK1's TMD, without any prior knowledge of its location, a result that is extremely unlikely to arise from an incorrect or random model.

The revised manuscript incorporated substantial new data and a rigorous and comprehensive response to the reviewers' critiques – the product of a monumental effort of ~ 6 months intensive work. In this context, we were genuinely surprised that the revision was deemed insufficient. We are concerned that the remaining objections – particularly those of Reviewer 3 – are not grounded in sound scientific reasoning and appear to reflect a preconceived opinion, rather than an evaluation of the data presented. Under these circumstances, it is difficult to identify any realistic additional work that would have satisfied this reviewer. **Below, I have included a brief point-by-point response to the report of our revised paper in justification of this rebuttal**.

Given the competitive nature of this field and the prevailing view regarding PINK1 localisation and function, we believe this work has encountered an unusually high level of resistance. To ensure a fair and objective resolution, we respectfully propose that an additional, independent expert reviewer be invited to evaluate the revised manuscript. Ideally this would be a senior authority in the mitochondrial protein import field, with a reputation for rigor and impartiality (particularly concerning PINK1), such as Profs. Nikolaus Pfanner or Agnieszka Chacinska.

Should an additional expert consider our work to meet *The EMBO Journal's* standards of rigor and significance, we believe that the most appropriate course of action would be to proceed with publication, incorporating minor revisions as needed (outlined below). This would allow the field to engage directly with the findings and pursue follow up studies. Given the likely importance of these results for our understanding of mitochondrial quality control and regulation, which are critical to human health, prolonged delay in peer review serves neither the community nor *The EMBO Journal*. We appreciate your careful consideration of this rebuttal, and look forward to your response.

With Best Wishes

Ian Collinson (on behalf also of Adam Grieve and Robin Corey)

Referee #1:

The authors have made substantial efforts to carefully address all points raised by the reviewers. In particular, they provide convincing new experimental data in the revised version that support the validity of the structural models. Not all critical points could be resolved, e.g. the how the model of a transient protein-conducting channel of Tim17 and Mgr2/ROMO1 as the main passageway for protein import into the matrix can be reconciled with the fact that knockout mutants lacking Mgr2 or ROMO1, respecitvely, display only a minor matrix translocation phenotype. However, in my view these minor shortcoming are acceptable here, because overall the manuscript has much imoproved in the revision. This elegant study provides very interesting new insights into the mechanisms of mitochondrial protein sorting as well as PINK1 biology and will be of great interest for the broad readership of the EMBO Journal. With respect to the competitive situation in this vivid area of research, I recommend to accept this paper in the present form now.

Referee #3:

General comment.

Lorriman et al have submitted a revised version of the manuscript which addresses many of the points we raise. However, I remain unconvinced by the structural modeling, which was not validated in a substantial way. The addition of the MB20 experiments adds value, but there are still some flaws. Detailed comments are found below.

Mentioning the new PINK1-TOM structure is indeed important to mention now, thus we appreciate the authors adding it to their paper. However, when mentioning the structure be sure to mention it is VDAC2, as there are three isoforms of VDAC. This is not specified in the paper.

This will be very easy to fix. We will of course refine our reference as directed to these important new results.

Here are the responses to each numbered points I raised previously.

1) The results with MB20 do strengthen the manuscript, but the immunoblot showing the PARL knockdown in Figure S14 is not convincing. We understand the PARL antibody is not good, but perhaps RT-PCR or even mass spec could be used to demonstrate PARL-KD.

We have been able to quantify this from the immunoblots (see below), which is quite conclusive. We could include this data. Mass spectrometry is not an appropriate approach for addressing this question.

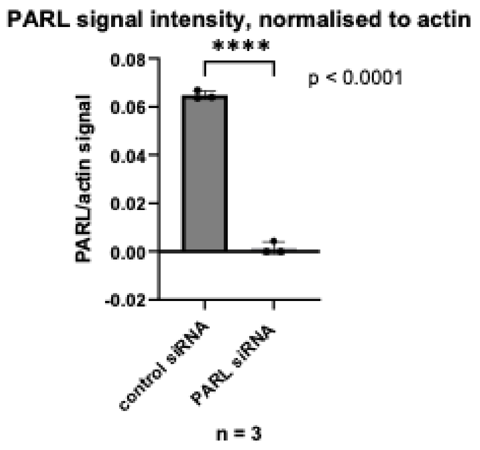

2) The ipSAE is indeed an improvement on the ipTM score, which is particularly well suited for cases such as this, where only a small region in a protein (like the PINK1 peptide) binds to another protein (PARL). However, it remains a prediction, and not an experimental validation. Having performed the modelling myself, with both AF2 and AF3, I can also see that the top 5 models don't converge well, with mean rmsd > 5 Ang between the 5 models for the segment of PINK1 100-113, after superposing the PARL domain. This is not a good sign; it doesn't prove the interaction doesn't exist (AlphaFold is prone to false negative), but it reduces our confidence in the model. Having written that, I would have less qualms about this if some experimental validation was provided (i.e. a mutagenesis to prove that a given interaction is important for the cleavage), but this was not provided for the PINK1-PARL interaction. The only new experiments that I can see are those with the ALALAL mutants in Figure 8, which improves the manuscript, but don't validate the structural model. I agree that the proximity of the PINK1 Phe104 to the active site serine in PARL suggests the model must be close to the real structure, but by presenting a single model of the structure, it provides a false sense of certainty about the structure. The authors should at least show an ensemble of the structure predicted by AlphaFold.

It is impossible for us to assess the quality of the referee's modelling, as no methodological details were provided (for example, which subset of atoms were included in the calculation, or whether the alignment was performed pairwise or against model 0). Irrespective of these substantial points, we feel that the referee's analysis is fundamentally flawed. They

say that they ‘superposed’ their PINK1 models onto the PARL domain and then measured the degree of structural deviation of the different PINK1 β-strand positions. While it is not very clear what they did exactly, we believe they have overlooked the important fact that (at least in our predictions) the PARL β-stranded loop, where PINK1 binds, is inherently flexible – as might be expected for a loop in this region of the membrane. This loop forms a stable β-strand interaction with PINK1, that survives during Molecular Dynamics simulations, so the PINK1 position is strongly linked to the conformation of the flexible PARL loop. Therefore, the referee’s approach likely simply captures the flexibility of the PARL β-stranded loop rather than meaningfully assessing the PINK1-PARL interface (the focus of this research). Therefore, we consider this analysis to be inappropriate, and uninformative.

We emphasise that the original PAE and the improved ipSAE analysis consistently indicate high confidence in the key feature of this study: the PINK1/PARL β-strand. Given the extreme experimental challenges associated with capturing this transient intra-membrane structural state – and the fact that a structure of PARL alone has not ever been determined – our predicted models represent the forefront of our understanding of this crucial interaction. Importantly, this interaction has been experimentally supported, and the model we present is highly plausible in light of established analogies to other intra-membrane proteases (*e.g.* γ-secretase), as well as the precise correspondence between the known active site of PARL and cleavage site of PINK1. We also argue that the ALALAL variant, which we designed to strongly disfavour helical unwinding and thereby enhance import, is in fact a convincing support of our structural and mechanistic models, and we are unclear why the referee claims otherwise.

We agree with the referee it is appropriate to be transparent about potential sources of uncertainty. To that end, we would be pleased to include all other AlphaFold models, along with associated analyses and confidence metrics, *via* our provided OSF link.

While the PINK1-PARL structural prediction is somewhat believable, the PARL:TIM23 supercomplex or PINK1-TIM23 are not. Again, the authors do acknowledge the speculative nature of these complexes, but it is misleading to show these structures as static models in Figure 4d,e, given that they are low confidence models. Furthermore, no PAE plots are shown for the PINK1-TIM23 complex. Unless the PAE plot confidently predicts an interaction between PINK1-TIM23, the structural model should be removed entirely. If there is a possible interaction, the authors must also demonstrate that the models converge.

The primary PINK1/PARL/TIM23 super-complex was made by combining two separate AlphaFold2 predictions, so it is impossible to present a PAE for this particular configuration; though we have presented this information for the separate models. We highlight that our analysis does not include any direct interactions between PINK1 and TIM23 – rather we present high confidence predictions for PINK1 with PARL and PARL with TIM23. Therefore, our currently presented data sufficiently support our claims. We will clarify this point further in the text.

In additional, they must provide experimental validation of these models, using site-specific mutagenesis.

We have done so. Experimental validation by site specific amino acid changes is included in the manuscript; such as the 3EA triple substitution and the Parkinson’s linked I111S, which gave us predicted and interpretable results in line with our findings. Therefore, the need for ‘site-specific mutagenesis’ has been addressed.

We explained in the previous rebuttal why some of the referees’ suggestions were not achievable from a pragmatic point of view, or due to time constraints, along with reasoning that some of the proposed experiments would not generate useful data. For instance, cysteine-cysteine disulphide analysis would not work (please see our previous response, below). To do this near neighbour analysis, we would need to deploy genetic reprogramming for incorporation of non-native photo activatable amino acids for more specific cross-linking, which would take considerable time and resources, and would represent a whole new study perhaps suitable for a future publication.

In addition to the confidence we have in the AF-modelling (see above), we would like to reiterate the power of Molecular Dynamics (MD) simulations and its potential for validation, which has not been recognised by the critical referee. MD simulations are built upon physical principles (force fields carefully refined using experimental physical chemistry data and dynamics based on classical Newtonian mechanics) and are entirely independent to AF (based on learning from pre-existing structures). Thus, the compliance of our AF structures to MD simulations is compelling. Indeed, the correct positioning by MD of tightly bound phospholipids to the AF models, has been experimentally verified elsewhere (Sim *et al.* 2023, ***Nature***), which tells us that the simulations represent reality. Moreover, the simulated models have recapitulated verifiable biochemical predictions (identification of PINK1’s cleavage site, shown here), in addition to novel structural features in PINK1 (the α/β-hybrid TMD). The similarity of this completely new configuration of the TMD of PINK1 (not seen before experimentally) in association with PARL, with other intra-membrane protease-substrate interaction is very close to proving that the structure is correct (again, it is almost impossible to conceive this similarity would have arisen from incorrect or random modelling). Moreover, this conformation has been experimentally validated by the expected biochemical outcome following its destabilising (by the ALALAL variant).

By the way, there is no figure legend description for Fig. S7e, and no legend description for the color code in Fig. S7d. Likewise, the Fig. S11 could better labeled. Subunits directly labelled on the figure would be a big help to get the key point of the figure across.

We agree; this can be rectified easily.

3) The authors are incorrect in their assumptions. Even though the NTE is far from the active site of PINK1, its interaction with TOM20 is critical for the activation, as disruption of this interaction abrogates PINK1's kinase activity. In turn, the folding (or lack) of PINK1 in the cytosol may affect the translocation kinetics of PINK1. While we understand that this paper is not concerned with PINK1 accumulation at the OMM, it should at least be stated that the function of PINK1

under damaged condition with the pep86 peptide was not investigated, and this in turn may affect PINK1's import kinetics.

This is a minor point with regard to our conclusions that PINK1 can target the mitochondrial matrix (as acknowledged by the referee). Therefore, we propose to state that we have not formally ruled out a consequence of pep86 in outer mitochondrial membrane accumulation.

4) Thanks for the clarification on the purification.

5) OK

6) Yes, we understood that, but then the authors should write on page 14: "Therefore, only the region between 91-150, including the TMD (containing PARL cleavage site) and the NTE, but not the OMS, are necessary and sufficient for association with the TIM23-complex and import." To make it clear that it's not just the TMD. I will also note that this contradicts previous results showing that deletion of the N-terminal MTS prevents PINK1 import into the matrix and renders PINK1 constitutively active (Okatsu et al 2015). The difference here may have to do with the point #3 above, i.e. that PINK1 folding (or misfolding by insertion of pep86) may affect import properties.

Again, this is not a major point, and we will clarify further in line with our response to point 3.

7) OK

Other comments:

8) Introduction: Line 26-27, page 4. It is worth mentioning OMA1 cleavage of PINK1 is induced under specific conditions that disrupt PINK1 accumulation (TOM7 deletion, OMS deletion, TIM deletion etc.). This is not clear in this new introduction.

We will amend.

9) On page 10, the authors added a new sentence "The more effective ionophore CCCP could not be used because of its incompatibility with the luciferase assay". This sentence is not accurate. To start with, antimycin A and oligomycin A are not protonophores; protonophores carry protons across membranes whereas antimycin A and oligomycin A are ETC inhibitors.

We do understand the action of antimycin A, oligomycin and CCCP, this was just a miswording. This is easily rectified by text. This point is inconsequential to the findings.

It's also surprising to read that they are not efficient in depolarizing, given they have been used in numerous and achieve complete depolarization. What concentrations of antimycin A and oligomycin A are being used in this study? This should be specified, maybe insufficient concentrations were being used (but we cannot tell).

This was an oversight and we shall include the concentrations. In our hands, the effects of antimycin A and oligomycin are limited (which we have shown before). We think that this discrepancy between the referee’s experience and ours is possibly due to a difference in model system; we applied these compounds to intact cells, rather than isolated mitochondria.

Dear Ian,

I have now heard back from an arbitrating fourth reviewer who had access to previous versions of the manuscript and referee reports. As you will see from the report below, the report is fully supportive of publication. I will now look at the remaining editorial points that need to be addressed and contact you again soon, but we can now move quickly towards publication.

Best wishes,

William

William Teale, PhD
Editor
The EMBO Journal
w.teale@embojournal.org

Instructions for preparing your revised manuscript:

Read our guidance for manuscript revisions and related editorial policies: https://link.springer.com/journal/44318/submission-guidelines#cms-Revised-submissions

Please also check that the title and abstract of the manuscript are brief, yet explicit, even to non-specialists.

When assembling figures, please refer to our figure preparation guideline in order to ensure proper formatting and readability in print as well as on screen:
https://media.springernature.com/original/springer-cms/rest/v1/content/27825798/data/v1

IMPORTANT: When you send the revision we will require
- a point-by-point response to the referees' comments, with a detailed description of the changes made (as a word file).
- a word file of the manuscript text.
- individual production quality figure files (one file per figure)
- a complete author checklist
- Expanded View files (replacing Supplementary Information)
- a Reagents and Tools Table as part of the Methods section

Please remember: Digital image enhancement is acceptable practice, as long as it accurately represents the original data and conforms to community standards. If a figure has been subjected to significant electronic manipulation, this must be noted in the figure legend or in the 'Methods' section. The editors reserve the right to request original versions of figures and the original images that were used to assemble the figure.

We realize that it is difficult to revise to a specific deadline. In the interest of protecting the conceptual advance provided by the work, we recommend a revision within 3 months (15th Jun 2026). Please discuss the revision progress ahead of this time with the editor if you require more time to complete the revisions. Use the link below to submit your revision:

https://emboj.msubmit.net/cgi-bin/main.plex

Referee #4:

I have carefully evaluated the revised manuscript, the constructive comments of the referees and the detailed response of the authors in both rounds. This study presents exciting insight in the molecular mechanisms of sorting and cleavage of the PINK1 kinase by mitochondria. By a combination of elegant experiments and modelling, the authors provide strong evidence for the proposed alternative pathways: ROMO1-Tim17 for matrix import and PARL-Tim17 for cleavage and retrotranslocation. In the first round of revision, the authors carefully revised the manuscript and included substantial additional data in support of their novel model. The PINK1(GLGLGL) versus PINK1(ALALAL) experiment in Fig. 8 fully supports the conclusions of the authors.

The role of PINK1 under physiological and pathophysiological conditions is a rapidly moving and competitive field with important implications for understanding mitochondrial quality control and regulation. I fully agree with the final conclusion of referee 1 that

the revised manuscript should be published very soon, including the minor revisions outlined by the authors in their second rebuttal letter.

University of BRISTOL

Ian Collinson

Professor of Biochemistry

School of Biochemistry
University Walk
Bristol BS8 1TD
United Kingdom

email: ian.collinson@bristol.ac.uk

Dr William Teale
Scientific Editor *EMBO J.*

26th March 2026

Dear Dr Teale,

Thank you and the reviewers for your decision to accept our manuscript following our rebuttal and the views of the fourth arbitrating reviewer (below). We have prepared a second revision of the manuscript with minor changes highlighted in the earlier rebuttal (further below). In addition to those changes we also corrected a few typographical errors and made a few minor stylistic changes, along with your requested editorial amendments.

Sincerely,

Ian Collinson, on behalf of all the authors.

Referee #4:

I have carefully evaluated the revised manuscript, the constructive comments of the referees and the detailed response of the authors in both rounds. This study presents exciting insight in the molecular mechanisms of sorting and cleavage of the PINK1 kinase by mitochondria. By a combination of elegant experiments and modelling, the authors provide strong evidence for the proposed alternative pathways: ROMO1-Tim17 for matrix import and PARL-Tim17 for cleavage and retrotranslocation. In the first round of revision, the authors carefully revised the manuscript and included substantial additional data in support of their novel model. The PINK1(GLGLGL) versus PINK1(ALALAL) experiment in Fig. 8 fully supports the conclusions of the authors.

The role of PINK1 under physiological and pathophysiological conditions is a rapidly moving and competitive field with important implications for understanding mitochondrial quality control and regulation. I fully agree with the final conclusion of referee 1 that the revised manuscript should be published very soon, including the minor revisions outlined by the authors in their second rebuttal letter.

Dear Ian,

I am pleased to inform you that your manuscript has been accepted for publication in the EMBO Journal.

Congratulations to you and all involved!

Your manuscript will be processed for publication by EMBO Press. It will be copy edited and you will receive page proofs prior to publication. Please note that you will be contacted by Springer Nature Author Services to complete licensing and payment information.

You may qualify for financial assistance for your publication charges - either via a Springer Nature fully open access agreement or an EMBO initiative. Check your eligibility: https://link.springer.com/journal/44318/how-to-publish-with-us

Should you be planning a Press Release on your article, please get in contact with embo_production@springernature.com as early as possible in order to coordinate publication and release dates.

If you have any questions, please do not hesitate to contact the Editorial Office. Thank you for your contribution to The EMBO Journal.

Best wishes,

William

William Teale, PhD
Editor
The EMBO Journal
w.teale@embojournal.org

*****
Please note that it is The EMBO Journal policy for the transcript of the editorial process (containing referee reports and your response letters) to be published as an online supplement to each paper. If you should prefer removal of any referee-only figures included in the point-by-point response(s), e.g. because they may still be used for future publication or because they have been reproduced from published work by others, please do let us know immediately via response email.
More information is available here: https://link.springer.com/partners/embo-press/editorial-policies#Peer%20review

** Click here to be directed to your login page: https://emboj.msubmit.net

EMBO Press Author Checklist

Corresponding Author Name: Ian Collinson	
Journal Submitted to: The EMBO Journal	
Manuscript Number: EMBOJ-2024-119845	

USEFUL LINKS FOR COMPLETING THIS FORM
The EMBO Journal - Author Guidelines
EMBO Reports - Author Guidelines
Molecular Systems Biology - Author Guidelines
EMBO Molecular Medicine - Author Guidelines

Reporting Checklist for Life Science Articles (updated January
This checklist is adapted from Materials Design Analysis Reporting (MDAR) Checklist for Authors. MDAR establishes a minimum set of requirements in transparent reporting in the life sciences (see Statement of Task: 10.31222/osf.io/9sm4x). Please follow the journal's guidelines in preparing your
Please note that a copy of this checklist will be published alongside your article.

Abridged guidelines for figures
1. Data
The data shown in figures should satisfy the following conditions:
- the data were obtained and processed according to the field's best practice and are presented to reflect the results of the experiments in an accurate and unbiased manner.
- ideally, figure panels should include only measurements that are directly comparable to each other and obtained with the same assay.
- plots include clearly labeled error bars for independent experiments and sample sizes. Unless justified, error bars should not be shown for technical
- if n<5, the individual data points from each experiment should be plotted. Any statistical test employed should be justified.
- Source Data should be included to report the data underlying figures according to the guidelines set out in the authorship guidelines on Data

2. Captions
Each figure caption should contain the following information, for each panel where they are relevant:
- a specification of the experimental system investigated (eg cell line, species name).
- the assay(s) and method(s) used to carry out the reported observations and measurements.
- an explicit mention of the biological and chemical entity(ies) that are being measured.
- an explicit mention of the biological and chemical entity(ies) that are altered/varied/perturbed in a controlled manner.
- the exact sample size (n) for each experimental group/condition, given as a number, not a range;
- a description of the sample collection allowing the reader to understand whether the samples represent technical or biological replicates (including how many animals, litters, cultures, etc.).
- a statement of how many times the experiment shown was independently replicated in the laboratory.
- definitions of statistical methods and measures:
  - common tests, such as t-test (please specify whether paired vs. unpaired), simple χ2 tests, Wilcoxon and Mann-Whitney tests, can be unambiguously identified by name only, but more complex techniques should be described in the methods section;
  - are tests one-sided or two-sided?
  - are there adjustments for multiple comparisons?
  - exact statistical test results, e.g., P values = x but not P values < x;
  - definition of 'center values' as median or average;
  - definition of error bars as s.d. or s.e.m.

Please complete ALL of the questions below.
Select "Not Applicable" only when the requested information is not relevant for your study.

Materials

Newly Created Materials	Information included in the manuscript?	In which section is the information available? (Reagents and Tools Table, Materials and Methods, Figures, Data Availability Section)
New materials and reagents need to be available; do any restrictions apply?	Not Applicable	Materials and Methods, and Appendix

Antibodies	Information included in the manuscript?	In which section is the information available? (Reagents and Tools Table, Materials and Methods, Figures, Data Availability Section)
For **antibodies** provide the following information:<br>- Commercial antibodies: RRID (if possible) or supplier name, catalogue number and or/clone number<br>- Non-commercial: RRID or citation	Not Applicable	

DNA and RNA sequences	Information included in the manuscript?	In which section is the information available? (Reagents and Tools Table, Materials and Methods, Figures, Data Availability Section)
**Short novel DNA or RNA including primers, probes**: provide the sequences.	Not Applicable	

Cell materials	Information included in the manuscript?	In which section is the information available? (Reagents and Tools Table, Materials and Methods, Figures, Data Availability Section)
**Cell lines:** Provide species information, strain. Provide accession number in repository **OR** supplier name, catalog number, clone number, and/**OR** RRID.	Not Applicable	
**Primary cultures:** Provide species, strain, sex of origin, genetic modification status.	Not Applicable	
Report if the cell lines were recently **authenticated** (e.g., by STR profiling) and tested for mycoplasma contamination.	Not Applicable	

Experimental animals	Information included in the manuscript?	In which section is the information available? (Reagents and Tools Table, Materials and Methods, Figures, Data Availability Section)
**Laboratory animals or Model organisms:** Provide species, strain, sex, age, genetic modification status. Provide accession number in repository **OR** supplier name, catalog number, clone number, **OR** RRID.	Not Applicable	
**Animal observed in or captured from the field:** Provide species, sex, and age where possible.	Not Applicable	
Please detail **housing and husbandry conditions**.	Not Applicable	

Plants and microbes	Information included in the manuscript?	In which section is the information available? (Reagents and Tools Table, Materials and Methods, Figures, Data Availability Section)
**Plants:** provide species and strain, ecotype and cultivar where relevant, unique accession number if available, and source (including location for collected wild specimens).	Not Applicable	
**Microbes:** provide species and strain, unique accession number if available, and source.	Not Applicable	

Human research participants	Information included in the manuscript?	In which section is the information available? (Reagents and Tools Table, Materials and Methods, Figures, Data Availability Section)
If collected and within the bounds of privacy constraints report on age, sex and gender or ethnicity for all study participants.	Not Applicable	

Core facilities	Information included in the manuscript?	In which section is the information available? (Reagents and Tools Table, Materials and Methods, Figures, Data Availability Section)
If your work benefited from core facilities, was their service mentioned in the acknowledgments section?	Not Applicable	

Design

Study protocol	Information included in the manuscript?	In which section is the information available? (Reagents and Tools Table, Materials and Methods, Figures, Data Availability Section)
If study protocol has been **pre-registered, provide DOI in the manuscript**. For clinical trials, provide the trial registration number **OR** cite DOI.	Not Applicable	
Report the **clinical trial registration number** (at ClinicalTrials.gov or equivalent), where applicable.	Not Applicable	

Laboratory protocol	Information included in the manuscript?	In which section is the information available? (Reagents and Tools Table, Materials and Methods, Figures, Data Availability Section)
Provide DOI OR other citation details if **external detailed step-by-step protocols** are available.	Not Applicable	

Experimental study design and statistics	Information included in the manuscript?	In which section is the information available? (Reagents and Tools Table, Materials and Methods, Figures, Data Availability Section)
Include a statement about **sample size** estimate even if no statistical methods were used.	Yes	Materials and Methods, and Figure Legends
Were any steps taken to minimize the effects of subjective bias when allocating animals/samples to treatment (e.g. **randomization procedure**)? If yes, have they been described?	Not Applicable	
Include a statement about **blinding** even if no blinding was done.	Not Applicable	
Describe **inclusion/exclusion criteria** if samples or animals were excluded from the analysis. Were the criteria pre-established?<br><br>If sample or data points were omitted from analysis, report if this was due to attrition or intentional exclusion and provide justification.	Not Applicable	
For every figure, are **statistical tests** justified as appropriate? Do the data meet the assumptions of the tests (e.g., normal distribution)? Describe any methods used to assess it. Is there an estimate of variation within each group of data? Is the variance similar between the groups that are being statistically compared?	Yes	Materials and Methods, and Figure Legends

Sample definition and in-laboratory replication	Information included in the manuscript?	In which section is the information available? (Reagents and Tools Table, Materials and Methods, Figures, Data Availability Section)
In the figure legends: state number of times the experiment was **replicated** in laboratory.	Yes	
In the figure legends: define whether data describe **technical or biological replicates**.	Yes	

Ethics

Ethics	Information included in the manuscript?	In which section is the information available? (Reagents and Tools Table, Materials and Methods, Figures, Data Availability Section)
Studies involving **human participants**: State details of **authority granting ethics approval** (IRB or equivalent committee(s), provide reference number for approval.	Not Applicable	
Studies involving **human participants**: Include a statement confirming that **informed consent** was obtained from all subjects and that the experiments conformed to the principles set out in the WMA Declaration of Helsinki and the Department of Health and Human Services Belmont Report.	Not Applicable	
Studies involving **human participants:** For publication of **patient photos**, include a statement confirming that consent to publish was obtained.	Not Applicable	
Studies involving experimental **animals**: State details of **authority granting ethics approval** (IRB or equivalent committee(s), provide reference number for approval. Include a statement of compliance with ethical regulations.	Not Applicable	
Studies involving **specimen and field samples:** State if relevant **permits** obtained, provide details of authority approving study; if none were required, explain why.	Not Applicable	

Dual Use Research of Concern (DURC)	Information included in the manuscript?	In which section is the information available? (Reagents and Tools Table, Materials and Methods, Figures, Data Availability Section)
Could your study fall under dual use research restrictions? Please check biosecurity documents and list of **select agents and toxins** (CDC): https://www.selectagents.gov/sat/list.htm	Not Applicable	
If you used a select agent, is the security level of the lab appropriate and reported in the manuscript?	Not Applicable	
If a study is subject to dual use research of concern regulations, is the name of the **authority granting approval and reference number** for the regulatory approval provided in the manuscript?	Not Applicable	

Reporting

The MDAR framework recommends adoption of discipline-specific guidelines, established and endorsed through community initiatives. Journals have their own policy about requiring specific guidelines and recommendations to complement MDAR.

Adherence to community standards	Information included in the manuscript?	In which section is the information available? (Reagents and Tools Table, Materials and Methods, Figures, Data Availability Section)
State if relevant guidelines or checklists (e.g., **ICMJE, MIBBI, ARRIVE, PRISMA**) have been followed or provided.	Not Applicable	
For **tumor marker prognostic studies,** we recommend that you follow the **REMARK** reporting guidelines (see link list at top right). See author guidelines, under 'Reporting Guidelines'. Please confirm you have followed these guidelines.	Not Applicable	
For **phase II and III randomized controlled trials**, please refer to the **CONSORT** flow diagram (see link list at top right) and submit the CONSORT checklist (see link list at top right) with your submission. See author guidelines, under 'Reporting Guidelines'. Please confirm you have submitted this list.	Not Applicable	

Data Availability

Data availability	Information included in the manuscript?	In which section is the information available? (Reagents and Tools Table, Materials and Methods, Figures, Data Availability Section)
Have **primary datasets** been deposited according to the journal's guidelines (see 'Data Deposition' section) and the respective accession numbers provided in the Data Availability Section?	Yes	Results and DATA AND MATERIALS AVAILABILITY
Were **human clinical and genomic datasets** deposited in a public access-controlled repository in accordance to ethical obligations to the patients and to the applicable consent agreement?	Not Applicable	
Are **computational models** that are central and integral to a study available without restrictions in a machine-readable form? Were the relevant accession numbers or links provided?	Not Applicable	
If publicly available data were reused, provide the respective **data citations in the reference list**.	Not Applicable	
